# Microbial communities of Auka hydrothermal sediments shed light on vent biogeography and the evolutionary history of thermophily

**DOI:** 10.1038/s41396-022-01222-x

**Published:** 2022-03-28

**Authors:** Daan R. Speth, Feiqiao B. Yu, Stephanie A. Connon, Sujung Lim, John S. Magyar, Manet E. Peña-Salinas, Stephen R. Quake, Victoria J. Orphan

**Affiliations:** 1 Division of Biology and Biological Engineering, California Institute of Technology, Pasadena, CA USA; 2 Division of Geological and Planetary Sciences, California Institute of Technology, Pasadena, CA USA; 3 Department of Bioengineering, Stanford University, Stanford, CA USA; 4 Chan Zuckerberg Biohub, San Francisco, CA USA; 5 Facultad de Ciencias Marinas, Universidad Autónoma de Baja California, Ensenada, Mexico; 6 Present Address: Max Planck Institute for Marine Microbiology, Bremen, Germany

## Abstract

Hydrothermal vents have been key to our understanding of the limits of life, and the metabolic and phylogenetic diversity of thermophilic organisms. Here we used environmental metagenomics combined with analysis of physicochemical data and 16S rRNA gene amplicons to characterize the sediment-hosted microorganisms at the recently discovered Auka vents in the Gulf of California. We recovered 325 metagenome assembled genomes (MAGs) representing 54 phyla, over 30% of those currently known, showing the microbial community in Auka hydrothermal sediments is highly diverse. 16S rRNA gene amplicon screening of 224 sediment samples across the vent field indicates that the MAGs retrieved from a single site are representative of the microbial community in the vent field sediments. Metabolic reconstruction of a vent-specific, deeply branching clade within the Desulfobacterota suggests these organisms metabolize sulfur using novel octaheme cytochrome-c proteins related to hydroxylamine oxidoreductase. Community-wide comparison between Auka MAGs and MAGs from Guaymas Basin revealed a remarkable 20% species-level overlap, suggestive of long-distance species transfer over 400 km and subsequent sediment colonization. Optimal growth temperature prediction on the Auka MAGs, and thousands of reference genomes, shows that thermophily is a trait that has evolved frequently. Taken together, our Auka vent field results offer new perspectives on our understanding of hydrothermal vent microbiology.

## Introduction

Microbial communities at hydrothermal vents have long been of interest for their impact on localized productivity and nutrient cycling in the deep ocean, as surface expressions of the subsurface biosphere, and as potential analogs for ocean life on icy moons. These communities differ strongly from the communities inhabiting the surrounding seafloor, but the variation between different hydrothermal areas is not well understood. The majority of well-studied high temperature vents are basalt-hosted, with hydrothermal fluid directly discharged from fissures into the overlying seawater [1]. In contrast, sediment-hosted hydrothermal vents such as those found in Guaymas Basin are distinctive for the interaction of the superheated fluid with overlying sediment. This interaction further alters the fluid composition through incorporating thermally-degraded organic compounds during advection to the seafloor, resulting in steep temperature gradients in the sediments and near-surface oil production [2].

This additional complexity makes sediment-hosted vent fields attractive study sites for microbial ecology [3]. Indeed, Guaymas Basin has proven to be a particularly rich source for discovery of novel metabolic capabilities of thermophilic microorganisms. Examples include thermophilic anaerobic oxidation of methane coupled to sulfate reduction by consortia of *Desulfofervidus* sp. bacteria and ANME-1 archaea [4, 5], anaerobic butane degradation by the sulfate-reducing bacterium *Desulfosarcina* BuS5 [6], and anaerobic butane oxidation by consortia of *Synthrophoarchaeum* sp. archaea and *Desulfofervidus* sp. bacteria [7]. In addition, some of the most extreme hyperthermophiles, *Methanopyrus kandleri* and *Pyrodictium abyssi*, with maximum measured growth temperatures of 122 °C and 110 °C, respectively, have been isolated from Guaymas Basin sediments and chimneys [8–10]. The outsized role of Guaymas Basin in discovery of microbial processes makes the recent discovery of the Auka vent field, a second sediment-hosted hydrothermal vent system along the same fault in the Gulf of California [11–13], especially exciting, as it provides a unique opportunity for comparative analyses of sediment-hosted hydrothermal vent systems.

Auka is located at >3650 m water depth in the Southern Pescadero Basin, a pull-apart basin at the southern tip of the Gulf of California, 400 km southeast of Guaymas Basin. The composition of the hydrothermal fluids at both sites is similar. The fluids are slightly acidic (pH 6), with high concentrations of methane (81 and 63.4 mmol kg^−1^), carbon dioxide (49.2 and 61.1 mmol kg^−1^), hydrogen sulfide (10.8 and 6 mmol kg^−1^), and hydrogen gas (2 and 3.4 mmol kg^−1^) at Auka and Guaymas respectively [12, 14]. The temperature of the fluids measured at chimney orifices is close to 300 °C at both locations. Due to these high temperatures, fluids advecting through the sediments at both sites contain thermogenic hydrocarbons, originating from the catagenesis of sediment organic matter.

While the similarities between both sites are striking, there are stark differences as well. At 3650 m Auka is the deepest known hydrothermal vent system in the Pacific Ocean, and more than twice as deep as Guaymas Basin. The thicker sediment cover at Guaymas (700–1000 m) [2, 15], results in higher load of thermogenic hydrocarbons than observed at Pescadero Basin, where the sediment thickness is estimated to be less than 50 m in the areas directly adjacent to the hydrothermal mounds [12]. This combination of overlapping and contrasting conditions between the two sites makes them prime targets for comparative analysis of their microbial communities to elucidate the factors shaping microbial communities at either site.

We analyzed 325 MAGs recovered from metagenomic sequencing of two sediment cores, complemented with a 16S rRNA gene based diversity survey from 29 sediment cores, to characterize the sediment microbial community at the Auka vent field. The diversity and genomic similarity was then compared between Auka MAGs and those recently reported from Guaymas Basin sediments [16–18], providing important insights into shared microbial species and patterns in vent biogeography. A detailed analysis of the genomes of Tharpellota bacteria, a novel lineage of deep-branching Desulfobacterota shared between Auka and Guaymas, revealed that these organisms have the genetic potential for anaerobic hydrocarbon degradation possibly using a novel terminal reductase. Finally, we determined the distribution of mesophily-hyperthermophily across these diverse lineages by adapting a genome-based optimal growth temperature prediction model [19], exploring the role temperature plays in shaping microbial communities in Gulf of California hydrothermal vent sediments and, more broadly, evolutionary patterns of thermophily across the tree of life.

## Methods

### Sample collection and shipboard processing

**Fig. 1:**
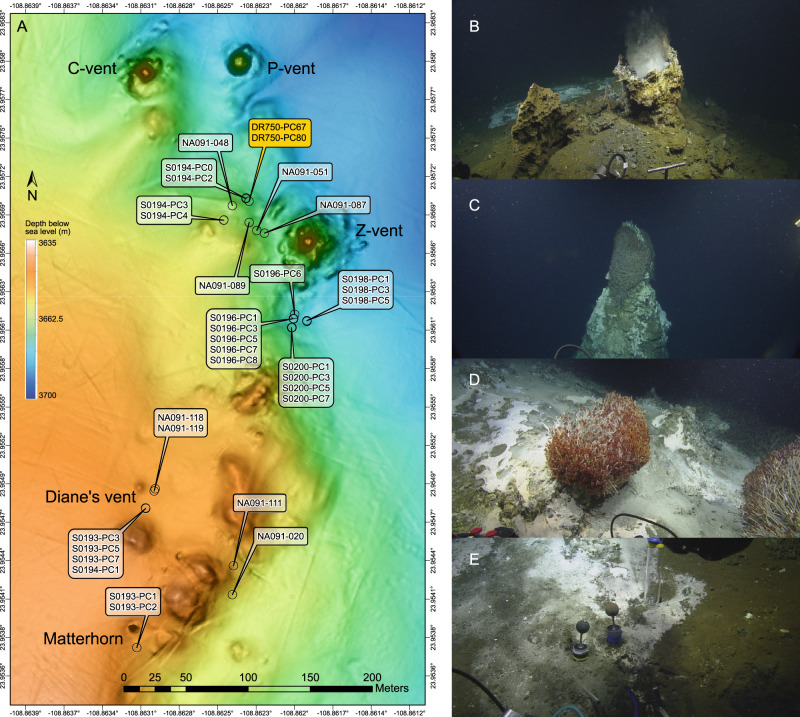
Overview of sampling area and impressions of Auka vent field. **A** Bathymetric map of Auka vent field with the most prominent sites of focused hydrothermal venting labeled by name. The sampling locations of push cores analyzed in this study are indicated, with the cores used for metagenomic sequencing highlighted in orange. DR750 is the dive number from cruise MBARI2015. S0193, S0194, S0196, S0198, and S0200 are dive numbers from cruise FK181031. Samples collected during cruise NA091 are indicated with cruise sample number **B** Diane’s vent, a chimney with distinctive clear hydrothermal fluid discharge clearly visible. **C** Top of the Matterhorn, a ~10 m high free-standing chimney, with the area around the central orifice fully covered in *Oasisia* sp. tubeworms. Microbial mat and shallow flange structures are visible on the sides of the chimney, indicative of diffuse fluid discharge through the chimney wall. **D** Carbonate platform covered in white and gray microbial mat with *Oasisia* sp. tubeworms (~20 cm tall) clustered around a localized spot of hydrothermal fluid discharge. This is a representative example of the unlabeled elevated mounds shown in **A**. **E** Sediment covered in microbial mat (at Diane’s vent) with heterogeneity of colors and textures indicating centimeter scale spatial heterogeneity in fluid diffusion through the sediment. Push core locations in **A** correspond to areas with prevalent microbial mats.

Samples were collected from Auka vent field in Pescadero Basin (Gulf of California, Mexico, 23.954, −108.863) on *R/V Western Flyer* in 2015 (MBARI2015), *E/V Nautilus* in 2017 (NA091), and *R/V Falkor* in 2018 (FK181031); information for the required sampling permits are provided in the funding acknowledgement section. Push core samples were collected using remotely operated vehicle (ROV) *Doc Ricketts* (MBARI2015), ROV *Hercules* (NA091), and ROV *SuBastian* (FK181031) from sediment covered areas with microbial mat cover and/or visible hydrothermal fluid flow (Fig. 1). Sites were deemed suitable for sampling if a 28 cm core could be fully inserted into the sediment. During dive DR750 on MBARI2015, two sediment cores were collected ~10 cm and ~15 cm distance from a site of focused hydrothermal fluid discharge close to Z-vent (Fig. 1, Supplementary Fig. S1). These two cores were split in surface (0–7 cm) and deep (7+ cm) horizons, and stored in heat sealed mylar at 4 °C, under nitrogen gas. During NA091, eight sediment cores were collected from locations across Auka vent field (Fig. 1, Supplementary Fig. S1). Those eight cores were sectioned in 1–3 cm thick horizons (Supplementary Data S1 and S2). 2 mL subsamples of wet sediment from each horizon were frozen at −80 °C for DNA extraction, and 6 mL sediment was centrifuged at 16,000 *g* for 2 min to collect sediment porewater. 0.25 mL filtered porewater was preserved in 0.25 mL 0.5M zinc acetate solution for later sulfide analysis. 0.25 mL filtered porewater was stored at −20 ^o^C for subsequent ion chromatography. During FK181031 (dives S0193, S0194, S0196, S0198, and S0200), 22 sediment cores were collected from locations across Auka vent field (Fig. 1, Supplementary Fig. S1). These 22 cores were sectioned in 1 or 3 cm horizons (Supplementary Data S1 and S2). 2 mL subsamples of wet sediment from each horizon were frozen at −80 °C for DNA extraction, and porewater was extracted from ~15 mL (1 cm horizons) or ~50 mL (3 cm horizons) sediment under nitrogen gas using a pneumatic sediment squeezer (KC Denmark A/S, Silkeborg, Denmark). 0.25 mL filtered porewater was preserved in 0.25 mL 0.5 M zinc acetate solution for sulfide analysis. 0.25 mL filtered porewater was stored at −20 °C for ion chromatography.

### Geochemistry

All filtered water samples were stored at −20 °C until analysis. Major ions were measured with a Dionex ICS-2000 (Dionex, Sunnyvale, CA, USA) ion chromatography system (Environmental Analysis Center, Caltech) with anion and cation columns running in parallel. An autosampler loads samples diluted 1:50 in 18 MΩ water run through an LC-Pak polisher (MilliporeSigma, Burlington, MA, USA) serially to a 10 µL sample loop on the anions channel, then a 10 µL sample loop on the cations channel. Both columns and detectors are maintained at 30 °C. The ion chromatography system was run as described previously [20] with the following modifications. Anions were resolved by a 2 mm Dionex IonPac AS19 analytical column protected by a 2 mm Dionex IonPac AG19 guard column (ThermoFisher, Waltham, MA, USA). A potassium hydroxide eluent generator cartridge generated a hydroxide gradient that was pumped at 0.25 mL min^−1^. The gradient was constant at 10 mM for 5 min, increased linearly to 48.5 mM at 27 min, then increased linearly to 50 mM at 40 min. A Dionex AERS 500 suppressor provided suppressed conductivity detection running on recycle mode with an applied current of 30 mA. Cations were resolved by a 4 mm Dionex IonPac CS16 analytical column protected by a 4 mm Dionex IonPac CG16 guard column. A methanesulfonic acid eluent generator cartridge generated a methanesulfonic acid gradient that was pumped at 0.36 mL min^−1^. The gradient was constant at 10 mM for 5 min, nonlinearly increased to 20 mM at 20 min (Chromeleon curve 7, concave up), and nonlinearly increased to 40 mM at 40 min (Chromeleon curve 1, concave down). A Dionex CERS 2 mm suppressor provided suppressed conductivity detection with an applied current of 32 mA. Chromatographic peaks were integrated by Chromeleon 7.2 using the Cobra algorithm, and were correlated to concentration by running known standards. The threshold of detection was ~10 µM for bromide and thiosulfate, 50 µM for ammonium, 100 µM for calcium, potassium, and sulfate, and 400 µM for magnesium.

Sulfide was determined colorimetrically using a protocol based on Cline [21]. Briefly, 22 μL of a 1:1 mixture of sample and 0.5 M zinc acetate was added to 198 μL milliQ water in a 96-well plate and mixed thoroughly. Immediately after mixing, 20 μL of the diluted sample was transferred to a second 96-well plate containing 180 μL milliQ water, resulting in one 96-well plate containing 10-fold diluted samples, and one 96-well plate containing 100-fold diluted samples with 200 μL final volume in each well. 20 μL of 1:1 mixture of Cline reagents (30 mM FeCl_3_ • 6H_2_O in 6 N HCl, and 11.5 mM N,N-dimethylphenylenediamine dihydrochloride (DPDD) in 6 N HCl) was added to each well and allowed to react for 1 h before measuring on a Tecan Sunrise 4.2 plate reader, using the Magellan software (version 7.3). Duplicates of all samples were run on the same 96-well plate. Sample concentration was determined by comparison to a 12-point standard curve added to each plate in duplicate, and treated identically to the samples.

### DNA extraction and metagenome sequencing

Two cores obtained during MBARI2015 from the same location, DR750-PC67 and DR750-PC80, were used for metagenome sequencing. These cores were split into two horizons (0–7 cm and 7+ cm), and both horizons were mixed and split in two subsamples each, resulting in a total eight samples. Of these eight samples, half were filtered through 10 μm mesh, assisted by sterilized artificial seawater, to deplete sediment-attached organisms or those forming large aggregates, and the other half was untreated. DNA was extracted from 0.25 g wet sediment using a CTAB/phenol/chloroform organic solvent extraction protocol modified from Zhou et al. [22] as previously described [23]. Cell lysis was achieved in a CTAB extraction buffer (10 g L^−1^ CTAB, 100 mM Tris, 100 mM EDTA, 100 mM sodium phosphate, 1.5 M NaCl pH 8) with serial addition of, and incubation with, lysozyme, proteinase K, and sodium dodecyl sulfate SDS. After cell lysis, DNA was recovered using phenol/chloroform/isoamylalcohol (25:24:1) at 65 °C, followed by an additional chloroform/isoamylalcohol (24:1) extraction to remove traces of phenol. After recovery of the aqueous phase, DNA was precipitated using isopropanol at room temperature, and washed with ice-cold (−20 °C) 70% ethanol and resuspended in nuclease-free water. Barcoded Nextera XT V2 libraries (Illumina, San Diego, CA, USA) were made with dual sequencing indices, pooled, and purified with 0.75 volumes of AMpure beads (Agencourt). The resulting libraries were sequenced on an HiSeq 2500 (Illumina, San Diego, CA, USA) with 2 x 125 protocol in high output mode (Elim Biopharmaceuticals, Hayward, CA, USA), resulting in 415 million paired reads.

### Assembly and binning

We adopted an iterative assembly and binning workflow to recover high quality metagenome assembled genomes (MAGs). Each single dataset was subsampled to 10 million reads, assembled using SPAdes (version 3.14.1) [24] and manually binned using Anvi'o [25]. Reads were mapped to the manual bins, using BWA (version 0.7.12-r1039) [26], filtered at >95% identity over >80% of the read length using BamM, (http://ecogenomics.github.io/BamM/) with matching reads removed from the dataset, and the process was repeated for all datasets until no more manual bins could be obtained, resulting in 50 manual bins representing abundant community members (Supplementary Data S3). Subsequently, all unmapped reads of all eight datasets, 194 million paired reads total, were pooled, co-assembled using MEGAHIT (version 1.2.9) [27], and binned using MetaBAT2 (version 2.12.2) [28], and then manually inspected and corrected using Anvi'o (version 6.2) [25]. The manual correction removed >1% of bases in 166 MetaBAT2 bins and >20% of bases from 54 MetaBAT2 bins, highlighting the value of manual inspection and curation. Bin quality was assessed using CheckM (version 1.1.2), and the single-copy marker gene sets included in Anvi’o. Bins with >50% completeness and <10% redundancy as determined by CheckM were retained. 8 additional bins with >10% redundancy were included after a second manual inspection (Supplementary Data S3). This workflow resulted in 331 MAGs. Six of these bins were only supported by reads from the datasets resulting from the sequencing of filtered samples. Those six MAGs were considered contamination and removed from all subsequent analyses. Taxonomic assignment of the MAGs was done using GTDB-tk (version 1.3.0) [29].

### MAG phylogeny and annotation

The two-domain phylogenetic tree of all 325 MAGs was generated using the single-copy marker gene sets “Archaea_76” and “Bacteria_71'' included in Anvi’o, using the 25 genes shared between these two sets of single-copy marker genes. Genes matching the 25 selected markers were extracted, aligned using MUSCLE (version 3.8.1551) [30], and the alignment concatenated using “anvi-get-sequences-for-hmm-hits”. The resulting concatenated alignment was converted to PHYLIP format using the ElConcatenero.py script (https://github.com/ODiogoSilva/ElConcatenero), and a phylogeny was calculated using RAxML (version 8.2.12) [31], with the PROTGAMMALG4X model [32] and the autoMRE bootstopping criterion resulting in 100 bootstrap replicates [33].

Gene calling on the MAGs was done using Prodigal (version 2.6.3) [34], and predicted genes were annotated using the DIAMOND [35] against the NCBI-NR database, PFAM [36], KEGG [37], COG [38], CDD [39], EGGNOG [40], and CATH [41] (Supplementary Data S4 and S5).

A clade we refer to as Tharpellota, comprising eight genomes (five obtained from Auka and three from Guaymas), was chosen for further analysis. The metabolic capabilities of this clade were compared to 419 other Desulfobacterota genomes (GTDB version 89) by functional enrichment analysis with anvi-compute-functional-enrichment using KEGG module assignments from anvi-estimate-metabolism (Anvi’o version 7, https://merenlab.org/software/anvio/help/main/programs/anvi-estimate-metabolism/). Multiheme cytochrome c fold family proteins were obtained from the GTDB using two iterations of sequence recruitment and filtering using a bit score ratio [42] for each of the five constituent protein families. The resulting sequence sets were merged and dereplicated, and the resulting 5855 proteins sequences were classified using ASM-clust with t-distributed stochastic neighborhood embedding (tSNE) perplexity value set to 500 [43].

### MAG reference phylogenies and optimal growth temperature prediction

Species overlap between the Auka vent field MAGs and other hydrothermal vent field MAGs was assessed using FastANI (version 1.3). 666 Guaymas basin MAGs [16–18] and 99 MAGs obtained from Juan de Fuca ridge hydrothermal fluid [44] were downloaded from NCBI. 348 bins from the Cayman Rise hydrothermal vent field [45] were retrieved as Anvi’o databases (version 2.1.0) from Figshare (https://figshare.com/projects/Mid-Cayman_Rise_Metagenome_Assembled_Genomes/20783). Contig fasta files were exported from the Anvi’o databases, and 131 bins over 50% completeness were used in ANI analysis.

Detailed phylogenetic placement of the Auka MAGs was obtained by downloading the genomes comprising the identified phyla, as well as sister phyla, from the genome taxonomy database (GTDB, version 89) [46]. In addition, 666 MAGs recently obtained from Guaymas basin were included in the reference set [16–18]. The genome set was split in 14 subsets, based on taxonomy and the GTDB reference tree, for alignment and tree calculation. Anvi’o databases were generated for all genomes, and the “Archaea_76” and “Bacteria_71” gene sets included in Anvi’o were used to generate concatenated alignments for archaeal and bacterial subsets, respectively, using MUSCLE (version 3.8.1551) [30]. Phylogenies were calculated using FastTree (version 2.1.7) [47].

Optimal growth temperatures (OGT) were predicted for the 325 MAGs and the reference genomes using the method described by Sauer and Wang [19] (https://github.com/DavidBSauer/OGT_prediction). Regression models modified by David Sauer to exclude 16S rRNA gene and genome size, to account for absence of 16S rRNA gene and genome incompleteness, were downloaded from https://github.com/DavidBSauer/OGT_prediction/tree/master/data/calculations/prediction/regression_models. The regression models for “Superkingdom Archaea” and “Superkingdom Bacteria” were chosen because of the diversity of the organisms in the dataset. The prediction_pipeline.py script was run as described in the documentation, with a custom “genomes_retrieved.txt” file (two tab-delimited columns, no header, columns: filename of gzip compressed contig fasta <tab> genome ID) and “species_taxonomic.txt” file (two tab-delimited columns, headers: “species <tab> superkingdom”, columns: “Genome ID <tab> Archaea|Bacteria”).

### 16S rRNA gene analyses

To gain insight in the microbial diversity of sediments underlying microbial mats across the Auka vent field, in addition to those provided by metagenomics at a single site, 216 samples derived from 29 sediment cores, and 8 samples from microbial mats or biofilms, were used for DNA extraction using the Qiagen Dneasy PowerSoil kit (Valencia, CA, USA) following the manufacturer’s protocol, with the exception that cells were lysed using MP Biomedicals FastPrep-24 (Irvine, CA, USA) for 45 s, at 5.5 m s^−1^. The V4-V5 region of the 16S rRNA gene was PCR amplified from the resulting 224 DNA extracts using the 515F/926R primer set [48] modified with Illumina adapters on 5′ end (515F 5′-TCGTCGGCAGCGTCAGATGTGTATAAGAGACAG-GTGYCAGCMGCCGCGGTAA-3′ and 926R 5′-GTCTCGTGGGCTCGGAGATGTGTATAAGAGACAG-CCGYCAATTYMTTTRAGTTT-3′).

Duplicate PCR reactions were set up for each sample with Q5 Hot Start High-Fidelity 2x Master Mix (New England Biolabs, Ipswich, MA, USA) in a 15 μL reaction volume, with annealing at 54 °C, for 28 cycles. The number of cycles was increased if no product was obtained, as detailed in the sample metadata (Supplementary Data S2). Duplicate PCR samples were then pooled and barcoded with Illumina Nextera XT index 2 primers that include unique 8-bp barcodes (P5 5′-AATGATACGGCGACCACCGAGATCTACAC-XXXXXXXX-TCGTCGGCAGCGTC-3′ and P7 5′-CAAGCAGAAGACGGCATACGAGAT-XXXXXXXX-GTCTCGTGGGCTCGG-3′). Amplification with barcoded primers used Q5 Hot Start PCR mixture but used 2.5 μL of product in 25 μL of total reaction volume, annealed at 66 °C, and cycled 10 times. Products were purified using Millipore-Sigma (St. Louis, MO, USA) MultiScreen Plate MSNU03010 with vacuum manifold and quantified using ThermoFisher Scientific (Waltham, MA, USA) QuantIT PicoGreen dsDNA Assay Kit P11496 on the BioRad CFX96 Touch Real-Time PCR Detection System. Barcoded samples were combined in equimolar amounts into single tubes and purified with Qiagen PCR Purification Kit 28104 before sequencing on a MiSeq (Illumina, San Diego, CA, USA) with the addition of 15–20% PhiX and with either a 2 × 250 or a 2 × 300 protocol (Laragen Inc., Culver City, CA, USA). Demultiplexed sequencing data was processed using QIIME2 (version 2020.2) [49], with DADA2 for amplicon sequence variant (ASV) calling [50] and Cutadapt for sequence quality trimming and primer removal [51]. resulted in 18,777 ASVs.

16S rRNA genes were recovered from the MAGs using the HMMs included in Anvi’o [25], and by de novo assembly of the combined metagenomic sequencing reads using phyloFlash [52]. After combining and dereplication of the two sets of sequences at 97% identity using USEARCH (version 11.0.667) [53], the resulting 284 sequences were aligned using MUSCLE (version 3.8.1551) [30], and a phylogenetic tree was calculated using RAxML (v8.2.12) [31], with the GTRGAMMA model and the autoMRE bootstopping criterion resulting in 400 bootstrap replicates [33]. Metagenome abundance of the 284 dereplicated 16S rRNA gene sequences was determined by mapping the combined reads of the shallow (0–7 cm) and deep (7+ cm) horizons of both cores onto the 16S rRNA gene sequences using BBmap, with a 95% identity filter [54]. Approximately 0.06% of the reads mapped on the genes, which is consistent with expectation based on the 16S rRNA gene length, and average genome length. Abundance of the 284 dereplicated 16S rRNA gene sequences in the amplicon sequencing data was determined by aligning all 18,777 ASVs to the 16S rRNA genes using BLAST (version 2.10.1) [55], and summing the number of reads represented by all ASVs with >97% identity to the assembled 16S rRNA genes.

Raw metagenome reads, assembled metagenome bins, and 16S rRNA gene amplicon sequencing data are available at NCBI under BioProject accession number PRJNA713414.

## Results

The Auka vent field is located at the western edge of the Southern Pescadero Basin, and occupies an area of ~200 by 600 m (Fig. 1). There are five prominent sites of vigorous fluid venting referred to as P-vent, C-vent, Z-vent, Diane’s vent, and Matterhorn, each characterized by prominent calcite chimneys (Fig. 1) [12]. Between these vents lie extensive carbonate platforms dotted with centimeter scale sites of hydrothermal fluid discharge supporting localized clumps of chemosynthetic *Oasisia* tubeworms [11]. These hydrothermal features are sediment-hosted, with sediment thickness in the area directly surrounding the chimneys and carbonate platforms estimated to be less than 50 m [12]. Throughout the vent field, but primarily near Diane’s vent and south of Z-vent, dispersed microbial mats covering the sediment surface indicate widespread advective hydrothermal discharge through the sediment (Fig. 1). The microbial mats exhibited a range of colors: pink, gray/white, yellow, and contain localized bright white spots surrounding focused flow of shimmering hydrothermal fluid (Supplementary Fig. S1). Sediment temperatures at 30 cm depth were elevated relative to the bottom seawater 2.4 °C, ranging from 10 °C to 177 °C, with the highest temperatures measured below yellow-colored mats, and beneath the bright white zones associated with visible hydrothermal fluid discharge. The release of oil droplets was observed during sediment coring to the south of Z-vent within a microbial mat, consistent with near-surface petroleum production within the sediment.

We performed shotgun metagenomic sequencing on sediments from two paired cores from a single site, DR750-PC67 and DR750-PC80, collected ~50 m from the main Z vent chimney (Fig. 1, orange label), to investigate the genomic potential of the microbial community in the sediments at this site. We complemented this in-depth analysis of a single site with 16S rRNA gene amplicon sequencing of 29 additional cores, as discussed in detail in the next section. The two cores used for metagenomic sequencing were collected within sediment covered by patchy yellow microbial mat, 5 cm from visible discharge of hydrothermal fluid and associated bright white spot on the sediment. DR750-PC80 was inserted closest to the fluid discharge, with DR750-PC67 directly adjacent (<2 cm apart), in a straight line away from the white spot (Supplementary Fig. S1). Each core was sectioned into two horizons (0–7 cm and 7+cm), resulting in four samples. To assess whether there were differences in the microbial community attached to, or forming, larger aggregates in the sediments, < 10 µm filtrate of a subsample of each horizon was processed for DNA extraction; doubling the total number of metagenomic samples to eight.

**Fig. 2:**
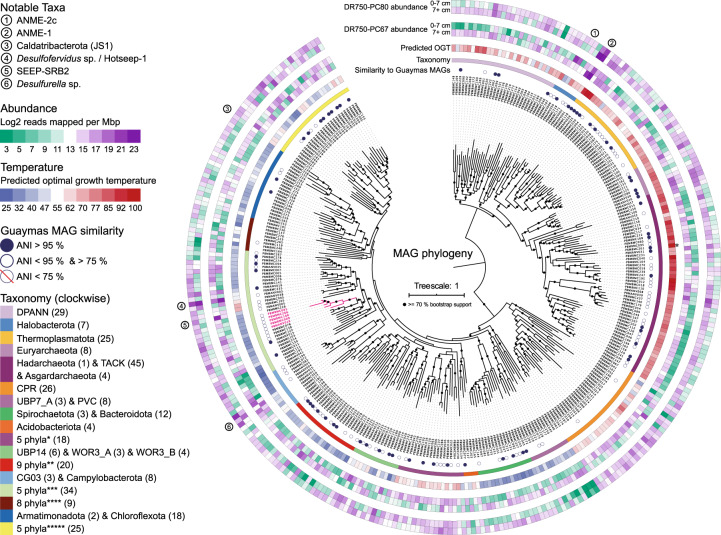
Concatenated marker gene phylogeny of the 325 Auka MAGs. Phylogeny of the 325 MAGs recovered from Auka vent field sediments, based on 25 concatenated marker genes. The scale bar indicates 1 substitution per site. From inside to outside, the concentric circles around the phylogeny indicate: the MAG ID, the average nucleotide identity (ANI) with MAGs previously retrieved from Guaymas Basin, phylum level taxonomy, MAG predicted optimal growth temperature (OGT), MAG abundance in the surface (0–7 cm) and deep (7+ cm) section of core DR750-PC67, and MAG abundance in the abundance in the surface (0–7 cm) and deep (7+ cm) section of core DR750-PC80. Numbers in parentheses indicate the number of MAGs belonging to that lineage in the dataset (All MAGs are shown in the figure). The Tharpellota branch (see text) is highlighted in pink. The predicted OGT of PB_MBMC_261 (111 °C) was outside the scale, and is indicated with an asterisk. Phyla corresponding to abbreviated groups in the taxonomy legend, with number of MAGs in parentheses: *1 UBP7 (1), Ratteibacteria (2), Omnitrophota (6), Calescibacterota (2), Aerophobota (7); **Fermentibacterota (1), Krumholzibacteriota (1), Cloacimonadota (4), Latescibacterota (3), Zixibacteria (2) KSB1 (1) SM23-31 (1), Calditrichota (1), Marinisomatota (6); ***Proteobacteria (2), Myxococcota (1), Desulfuromonadota (3), Desulfobacterota_A (5), Desulfobacterota (23); ****UBP3 (1), Sumerlaeota (1), RBG-13-66-14 (1), Poribacteria (1), Hydrogenedentota (1), Eremiobacterota (1), Firmicutes (1), Firmicutes_A (2); *****Caldatribacteriota (2), Synergistota (3), Caldisericota (3), Bipolaricaulota (6), Thermotogota (11).

After sequencing, assembly, and binning, we retrieved 331 MAGs with estimated completeness over 50%. Recovered MAGs were highly diverse, including 212 bacteria and 119 archaea, and representing 54 different phyla based on the genome taxonomy database (GTDB) assignment [29] (Fig. 2, Supplementary Data S3). 105 of these MAGs were estimated to be >90% complete with less than 5% contamination, and 111 MAGs contained (fragments of) a 16S rRNA gene (Supplementary Data S3) allowing direct comparison with the more extensive 16S rRNA gene amplicon survey data from Auka.

15 MAGs had more than twofold lower read coverage in the datasets resulting from sequencing the <10 μm filtered fraction than in the datasets resulting from sequencing the untreated samples (Supplementary Data S3). The most depleted MAG in this fraction was a dominant ANME-2c archaeon, which is consistent with members of this methanotrophic clade frequently forming multi-celled consortia >10 μm in association with syntrophic sulfate-reducing bacteria (SRB). Notably, the putative sulfate-reducing partner of ANME-2c (e.g. Desulfobacterota SEEP-SRB2), was also depleted (1.88 fold) in the filtered fraction. Another clade depleted in the filtered fraction were the heterotrophic bacteria *Izimaplasma* originally described from methane cold seeps [56, 57]. While anaerobic enrichments of this clade consisted of free-living organisms [56], their depletion in the filtered fraction could be due to association with larger organic particles for heterotrophic growth on DNA [57]. Notably, all four recovered MAGs belonging to the *Odinarchaeota* clade of Asgard archaea were also strongly depleted in the filtered fraction. The morphology and eco-physiology of *Odinarchaeota* is poorly characterized, and these findings may suggest an association with sediment particles or other microorganisms, or perhaps linked to an increased effective cell size due to unusual morphology, as observed for *Prometheoarchaeum* MK-D1, the only cultured member of the Asgard archaea [58]. None of the four ANME-1 or three *Desulfofervidales* MAGs were strongly depleted in the filtered sample, consistent with previous observations that sediment-hosted ANME-1 often occur as single cells, and tend to form less well-structured aggregates with SRB relative to ANME-2 consortia [4, 59].

Conversely, 14 MAGs were >2-fold enriched in the filtered fraction, suggesting limited association with the sediment matrix relative to other community members. These 14 MAGs represented 12 bacteria (seven Proteobacteria, two Synergistota, one Desulfobacterota, one Poribacteria, and one Thermotogota), and two Thermoplasmatota archaea (Supplementary Data S3). Six of these MAGs (three Alphaproteobacteria and three Gammaproteobacteria) were not supported by reads derived from unfiltered samples, and were therefore considered contamination and removed from further analyses, leaving 325 MAGs representing 54 phyla (Fig. 2). Combined, these 325 MAGs accounted for 40–60% of the reads in the datasets from unfiltered samples.

The 325 MAGs recovered from 2 sediment cores describe the microbial community in the sediments of Auka vent field at a single location. We conducted an area-wide survey using 16S rRNA gene amplicon sequencing of 29 additional sediment push cores to place our genomic data in broader context, and to better understand the sediment microbial community structure and distribution patterns in the total vent area in relation to the physicochemical parameters. These additional push cores, representing 224 total samples, were collected by remotely operated vehicle during expeditions in 2017 and 2018 (Fig. 1, Supplementary Fig. S1). Porewater geochemical profiles for 22 of these cores showed a variable degree of mixing of hydrothermal fluids with seawater, as evidenced by a steep drop in magnesium concentration with depth [60], and concurrent increases in calcium and potassium concentration (Supplementary Figs. S2 and S3, Supplementary Data S1). Consistent with a variable degree of mixing, temperature measured at 30 cm depth in the sediments varied greatly, ranging from 10 °C to 177 °C, a range similar to reports from Guaymas basin sediments [61–64]. Concentrations of oxidized nitrogen species were low, with nitrite below the detection limit in all measured porefluids. Nitrate was below the detection limit in 132 of 179 measured samples, and only consistently detected in three cores (S0193 PC7, S0194 PC0, and S0200 PC5), while ammonium was detected in all but three measured samples and reached concentrations as high as 16 mM, likely as a result of thermal degradation of organic matter [65]. Porewater sulfide profiles frequently showed maxima up to 12 mM between 5 and 15 cm below the sediment surface. Sulfate concentrations dropped below the detection limit in the top 20 cm below the seabed in 14 cores, attributed to a combination of microbial sulfate reduction and seawater mixing with sulfate-depleted hydrothermal fluid. In cores with the highest inferred flux of hydrothermal fluid (e.g. NA091-119 and S200-PC1), porewater sulfate concentrations were consistently below 10 mM at all depths (Supplementary Figs. S2 and S3).

**Fig. 3:**
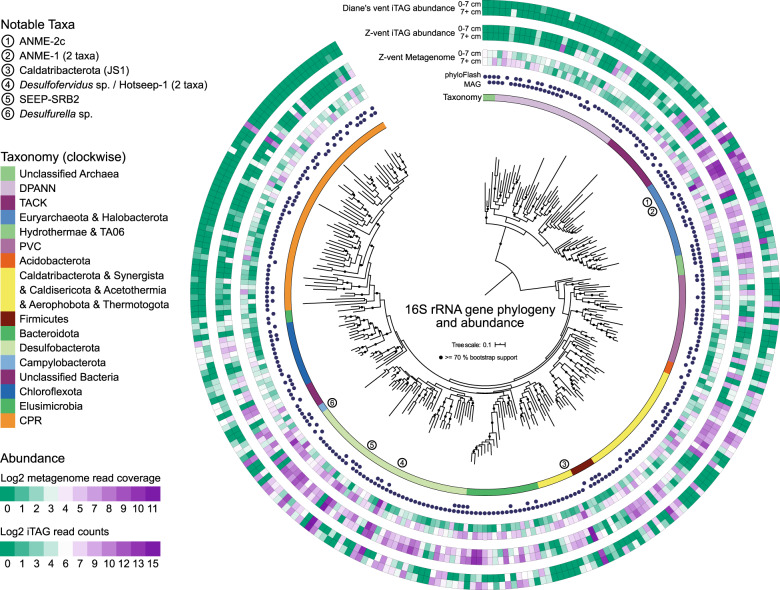
16 rRNA gene phylogeny and abundance in metagenome and amplicon data. Phylogeny of 284 16S rRNA genes reconstructed from the metagenomic data. From inside to outside the concentric circles around the phylogenetic tree indicate: the taxonomy of the major clades in the phylogeny, as assigned by Silva138, the recovery of the sequence through phyloFlash and/or annotation of the retrieved MAGs, the abundance in the surface (0–7 cm) and deep (7+ cm) section of the cores used for metagenomic sequencing, the abundance in the surface (0–7 cm) and deep (7+ cm) sections of the cores retrieved from Z-vent through amplicon sequencing, and the abundance in the shallow (0–7 cm) and deep (7+ cm) sections of the cores retrieved from Diane’s vent through amplicon sequencing. Circled numbers highlight abundant taxa discussed in the main text.

The taxa detected in our 16S rRNA gene amplicon survey were broadly similar in the 29 cores (Supplementary Figs. S4–S32). A comparison of the amplicon sequences with 16S rRNA genes retrieved from the metagenome using PhyloFlash [52], and assembled 16S rRNA genes from the MAGs, showed congruence between the most abundant taxa in all 29 cores and dominant taxa recovered from metagenome sequencing (Fig. 3; Supplementary Fig. S33). We obtained 284 16S rRNA gene sequences from the metagenomes, and compared all ASVs obtained in the amplicon analysis to these sequences. 1909 of the 18,777 ASV (10%) had > = 97% sequence identity with a metagenome-derived 16S rRNA gene sequence. These 1909 ASV were among the most abundant sequences in the dataset, accounting for 946,230 of the 2,057,639 counts (46%). In addition, for the majority of samples more than 50% of the reads had > = 97% identity to the metagenome-derived 16S rRNA gene sequences, irrespective of their location in the vent field (Supplementary Fig. S33). This shows that the MAGs assembled from a single location (2 adjacent cores) provides representative insight of the microbial community within the greater vent field. We note that several of the samples with the least matches to the metagenome-derived 16S rRNA gene sequences are the deepest horizons of the hottest cores. Additional amplification cycles were required to retrieve product from those samples (Supplementary data S2), and they deviate sharply from the community in the overlying horizons (e.g. Supplementary Figs. S16 and S26), thus may not represent the in situ community.

The overall distribution pattern in Auka sediments indicates widespread distribution of sulfide/sulfur-oxidizing Campylobacterota (formerly Epsilonproteobacteria) and putatively heterotrophic Bacteroidota as part of the surface microbial mat assemblage, with limited recovery of sulfur-oxidizing *Beggiatoa* (Supplementary text). Within the underlying sediment, ANME-2c archaea and Seep-SRB2 sulfate-reducing bacteria (SRB) of the *Dissulfuribacteraceae*, linked to the sulfate-dependent anaerobic oxidation of methane were dominant near the sediment surface, with consortia of ANME-1 archaea and *Desulfofervidus* sp. SRB were highly abundant in deeper sediment horizons. This ANME niche separation along temperature and geochemical gradients has been reported previously from Guaymas Basin, with ANME-2c observed in lower temperature cores, and ANME-1 in higher temperature cores [4, 61, 66]. Besides temperature, sulfate concentration may also contribute to niche separation at Auka, with ANME-1 phylotypes dominant in horizons corresponding to lower sulfate concentrations, a pattern that has also been described from cold seeps [67]. In several cores (e.g. NA091-118 and 119) ASV distribution showed further depth stratification of ANME-1 phylotypes, with ANME-1a ASVs co-occurring with Seep-SRB2 phylotypes in shallower horizons, again consistent with observations from methane cold seeps [68]. The 16S rRNA gene amplicon sequences in the deeper horizons annotated as ANME-1 are identical to sequences previously identified as the ANME-1 Guaymas group [61, 62] (Supplementary Figs. S11 and S12). Other clades present in high abundance are *Desulfobacteraceae* in the shallow sediments, with Caldatribacterota (JS1), Thermoplasmatota (DHVEG-2), and Thermotogota ASV’s common in the deeper horizons (Fig. 3). Consistent with the variation in porewater ion concentrations from hydrothermal fluid advection, the depth distribution of taxa varied between sediment cores, with higher inferred hydrothermal fluid input corresponding to limited detection of ANME-2c, higher abundance of ANME-1 in the shallow horizons, and appearance of hyperthermophilic lineages (e.g. family Methanopyraceae, class Thermoproteia) in the deeper horizons. A full summary of sediment 16S rRNA gene diversity from the 29-core survey is provided in Supplementary Figs. S4–S32 and Supplementary Data S2, S6 and S7.

**Fig. 4:**
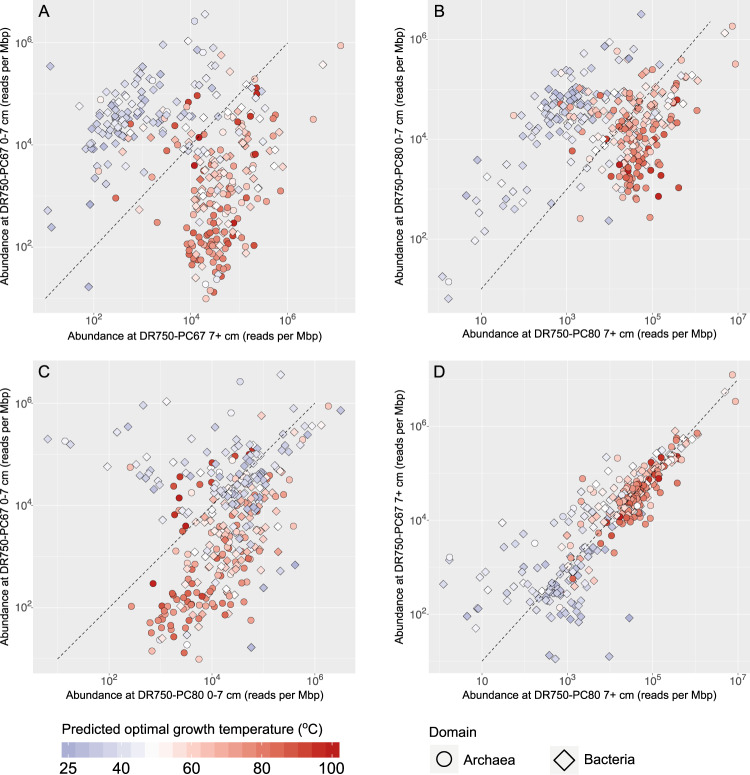
Abundance and predicted optimal growth temperature of the 325 Auka MAGs. Scatter plots showing reads per million base pairs of the Auka MAGs, as a proxy for organism abundance, in the sampled cores with point color corresponding to predicted optimal growth temperature (OGT) of each MAG. The dashed line represents equal abundance between both samples. **A** Comparison of MAG abundance between the DR750-PC67 surface horizon (0–7 cm) and DR750-PC67 deep horizon (7+ cm). **B** Comparison of MAG abundance between the DR750-PC80 surface horizon (0–7 cm) and DR750-PC80 deep horizon (7+ cm). **C** Comparison of MAG abundance between the surface horizons of both cores. **D** Comparison of MAG abundance between the deep horizons of both cores.

Both the 16S rRNA gene diversity analyses and the metagenomic sequencing indicate community stratification by depth in the sediment. Reducing conditions are likely prevalent throughout the advection dominated sediments underlying microbial mats at Auka (below the upper millimeters), as observed for Guaymas Basin [64]. We expect temperature to play a major role in shaping community structure, because of the high degree of hydrothermal fluid mixing and observed steep thermal gradients (up to 7 °C cm^−1^). While broad trends in relative abundance of sediment-hosted microbial community members along a temperature gradient have been shown for Guaymas Basin and other hydrothermal sites [69–71], the relationship of these trends to optimal growth temperature (OGT) is lacking for the majority of taxa due to the lack of cultured representatives. Genomic data is shedding new light on physiological characteristics like OGT, with strong correlations observed between select genomic features and OGT for cultured bacteria and archaea (ref. [19]. and references therein). This knowledge was recently synthesized and incorporated into an OGT prediction model, and used to accurately predict OGTs of phylogenetically diverse cultured microorganisms [19]. Here we apply a modified version of this model (see methods), and used this to estimate OGT for the environmental MAGs recovered from the Auka vents, Guaymas Basin, and a selected set of over 4000 archaeal and bacterial genomes from the GTDB (Fig. 2, Supplementary Data S3). For several microorganisms with known OGT or enrichment temperature, these genome-based predictions were largely consistent with reported values. For example, the predicted OGTs of the three Auka *Desulfofervidales* MAGs were 52 °C, 53 °C, and 60 °C, close to the experimentally determined OGT of 60 °C for *Desulfofervidus auxili* in pure culture [72] and the predicted OGT of the two ANME-1 MAGs in the GB60 clade were 63 °C and 65 °C, also close to the 60 °C enrichment temperature of the GB60 enrichment culture [4] (Fig. 2, Supplementary Data S3). Predicted OGT of the MAGs correlated with their abundance (approximated by read coverage) as a function of sediment depth, with most MAGs with OGTs over 50 °C showing higher abundance in the >7 cm horizon in both cores. Notably, this OGT depth trend was less pronounced in core DR750-PC80, collected directly adjacent to localized hydrothermal fluid discharge, compared to core DR750-PC67 which was collected on the far side of DR750-PC80, with the closest edge ~10 cm further away from the fluid source (Fig. 4, Supplementary Fig. 1). While sediment temperatures were not measured for these cores, the visible fluid discharge at the seabed indicates that core DR750-PC80 likely was exposed to a greater flux of hydrothermal fluids and steeper temperature gradients relative to PC67, potentially explaining the higher abundance of MAGs with predicted OGT values over 50 °C in the 0–7 cm horizon.

In addition to the correlation between predicted OGT and increasing abundance in lower horizons, there also is a clear correlation between predicted OGT and phylogeny (Fig. 2). Using our MAGs and reference data from the genome taxonomy database (GTDB) [46], we assessed the phylogenetic signal of predicted OGT in more detail. After taxonomic assignment [29], Auka MAGs, recent Guaymas MAGs [16–18], and reference genomes from the GTDB (version 89) were used to construct 14 clade-specific phylogenies with corresponding OGT predictions, representing a total of 5111 genomes, including all archaea and the majority of bacterial phyla included in GTDB version 89 (Supplementary Figs. S34–S47). Consistent with surveys of cultured organisms [20], the maximum predicted OGT in the 5111 genomes is considerably higher for the Archaea (117 °C) than for the Bacteria (89 °C); (Fig. 2). In addition to the higher maximum predicted OGT for archaea, we also observe that archaea are more abundant in the deeper horizons of both DR750-PC67 and DR750-PC80, possibly linked to their thermophilic adaptations.

Within the 14 phylogenies, organisms with predicted (hyper)thermophilic OGT values grouped together in distinct lineages, often emerging from lineages with predicted mesophilic OGT values, suggesting thermophilic adaptation is common throughout both the bacterial and archaeal domains of the tree of life, and frequently persists in a lineage once acquired (Supplementary Figs. S34–S47). On the other hand, there are several examples of mesophilic lineages evolving from (hyper)thermophilic ancestors, such as the Nitrososphaeria (formerly Thaumarchaea, Supplementary Fig. S34), and the Ferroplasmaceae (Supplementary Fig. S38), suggesting that thermophily is a reversible trait. Archaea have higher predicted OGTs than bacteria at Auka, and are more abundant in the deeper, hotter sediment horizons. Whether the Archaea are ancestrally thermophilic, as previously proposed [73], is unclear from our analyses. The OGTs predicted for MAGs retrieved from Auka and for previously published MAGs from Guaymas Basin indicate the occurrence of hyperthermophilic microorganisms across many phyla at these sites, with one MAG from an uncultured member of the Thermoproteia class from Guaymas Basin having the highest predicted OGT of all organisms analyzed, at 117 °C. Among the Auka MAGs, a member from the same clade within the Thermoproteia class had a predicted OGT of 111 °C (Fig. 3, Supplementary Fig. S34, Supplementary Data S3). These OGT values are higher than any experimentally determined OGT, and should thus be treated with care, as discussed in more detail below.

**Fig. 5:**
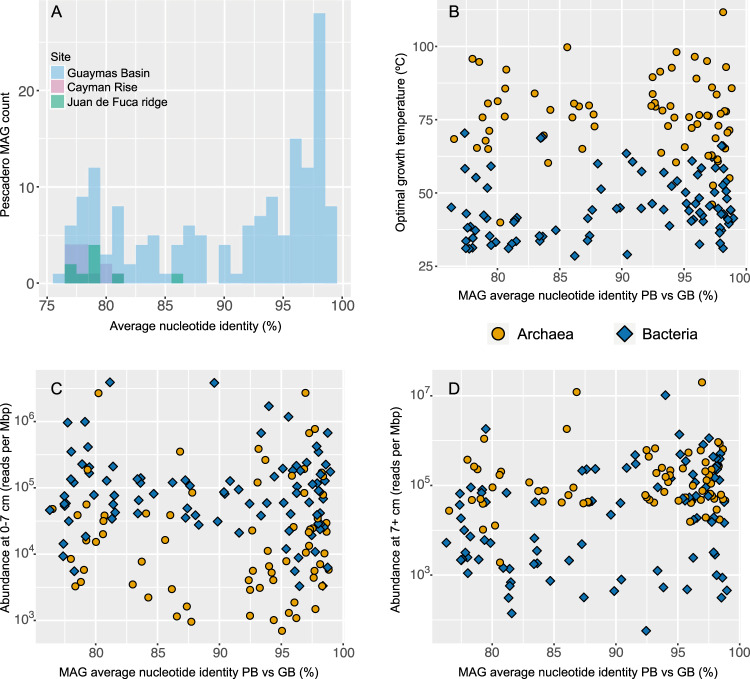
Community similarity between Pescadero Basin and other hydrothermal sites. **A** Histograms of the Auka MAGs with average nucleotide identity (ANI) greater than 75% with MAGs obtained from Guaymas Basin (161), Cayman Rise (12), and Juan de Fuca ridge (9), indicating a high community similarity between Auka and Guaymas Basin. **B** Scatterplot of Auka vs Guaymas Basin (GB) MAG ANI and predicted optimal growth temperature (OGT), showing no correlation between OGT and ANI. **C** Scatterplot of Auka vs GB MAG ANI and MAG abundance in the surface horizons, indicating no correlation between surface abundance and ANI. **D** Scatterplot of Auka vs GB MAG ANI and MAG abundance in the deep horizons, indicating no correlation between abundance in deeper horizons and ANI.

In addition to clade-specific adaptation to high temperature, the phylogenetic analysis showed that Guaymas Basin and Auka vent fields have substantial community overlap. We calculated average nucleotide identity (ANI) between all MAGs for both the Auka and Guaymas datasets to further quantify this overlap in microbial communities between these sites. This analysis revealed that 68 Auka MAGs, representing 23 bacterial and archaeal phyla, have ANI values that are >95% to MAGs from Guaymas Basin sediments [16–18], corresponding to nearly 20% species overlap between these geographically distant vent fields at substantially different depths in the Gulf of California [74, 75] (Figs. 2 and 5, Supplementary Data S3). Another 93 Auka MAGs had ANI values between 75 and 95% with MAGs from Guaymas, further showing broad community similarity. Inspecting 132 low completeness bins (0–49% completeness; Supplementary Data S8) that we did not include in our analyses, showed that 10 of these bins had ANI values >95% with bins from Guaymas Basin, and a further 38 bins had ANI values between 75 and 95% with MAGs from Guaymas Basin. Previous work has shown that the community at Guaymas Basin is distinct from those at basalt-hosted and ultramafic systems [76]. Indeed, a comparison with the MAGs retrieved from sampling deep hydrothermal fluids at Juan de Fuca ridge in the north Pacific [44], and hydrothermal fluids from mafic and ultramafic vent sites at Cayman rise in the Caribbean [45] showed only 9 and 12 MAGs with ANI above the 75% threshold value to the MAGs from Auka respectively, and no MAGs with >85% ANI in either dataset (Fig. 5). It has to be noted that the distance between either Juan de Fuca ridge or Cayman rise and the Gulf of California is much larger than the distance between Auka and Guaymas basin.

We hypothesize that the observed 20% species overlap is due to continuous transfer of microbial populations between Auka and Guaymas Basin. The exchange over the ~400 km between both sites may be facilitated by hydrothermal plumes, which have been shown to rise at least 900 m upwards from the vents at Guaymas Basin [77] and harbor distinct microbial communities from the surrounding seawater [1, 78, 79]. In addition, long distance transfer of thermophilic microorganisms through ocean currents has been documented [80, 81], and the open ocean has been shown to contain a “seed bank” of hydrothermal vent taxa [82]. Several organisms in this seed bank were also shown to be culturable from seawater particulates [83], indicating that plume mediated microbial population transfer throughout the Gulf of California is highly likely. Although the most prominent direction of transfer is not discernible from the microbial community structure, the prevailing deepwater current in the Gulf of California flows to the north [84].

Considering that hydrothermal plumes likely facilitate a constant flux of organisms between both basins, the species overlap of 20% indicates selection at dispersal, transfer, colonization, or a combination of these. As there is a strong correlation between temperature and community composition at Auka, we investigated whether the predicted OGT of MAGs correlated with ANI values between Auka and Guaymas Basin, but found no correlation, with both mesophilic and thermophilic groups represented among the 20% (Fig. 5). In addition, there was no correlation with ANI values and MAG abundance in surface sediments (Fig. 5), or MAG abundance in deeper horizons, with both abundant and rare taxa represented (Fig. 5). The latter observation contrasts previous work showing that abundant taxa were more likely to be cosmopolitan [85]. The sediment MAGs with species-level overlap between the Gulf of California vent sites are phylogenetically and physiologically diverse, suggesting there is not a single determinant of transfer and colonization success.

Based on these results, we hypothesize that the species overlap between Auka and Guaymas is primarily driven by the niches created from similar environmental conditions in the hydrothermal vent sediments and deeply sourced fluids [12], selecting for colonization by a subset of the transferred microbial community. In addition, the distribution of ANI values may indicate ongoing speciation between populations within the communities at the two sites. Previous studies have shown a gap in pairwise ANI values in the range 85–95% which was used to support 95% ANI as the species cutoff [74, 75]. While there is a clear peak of ANI values above 95% between Auka and Guaymas MAGs, an additional 25 MAGs (8% of the community) share ANI values between 90 and 95% showing a less pronounced gap in ANI values than previously observed (Fig. 5). These MAGs may represent lineages undergoing speciation after immigration and colonization of the new vent site.

Thus far, the genomic determinants for colonization success have not been established, potentially because such factors are likely to be lineage specific. For example, it is striking that strains of the same archaeal ANME-1 species are the most abundant organism in sediments from both the Auka vent field and the Guaymas basin, but that the other six ANME-1 MAGs (three at each site) belong to distinct lineages, suggesting niche differentiation (Supplementary Fig. S37). 16S rRNA gene amplicon sequencing, which was done at higher sediment depth resolution, showed differential distribution of ANME-1 lineages in multiple sediment cores at Auka, further supporting niche differentiation between them (Supplementary Figs. S4–S32).

Beyond implications for biogeography, the lineages largely or fully consisting of organisms found at Auka and Guaymas are of interest for future comparative genomics work. For example, several Aerophobota with OGT’s between 50 °C and 68 °C were detected in both Auka and Guaymas Basin (Supplementary Fig. S39). MAGs from this phylum were recently also retrieved from other hydrocarbon-rich environments, including methane cold seeps in the South China Sea [86] and areas of petroleum seepage in the Gulf of Mexico [87], making this clade a promising target for genome guided metabolic predictions, and enrichment cultivation focused on anaerobic hydrocarbon degradation. Another example is a deep-branching clade either within, or sister to, the Desulfobacterota phylum (Supplementary Fig. S44), that was originally discovered in Guaymas Basin [17] and is well represented in the surface layers of Auka vent field sediments. The Guaymas basin MAGs representing this group (indicated as DQWO01) were also included in a recent large-scale analysis of Desulfobacterota metabolic potential [88]. We recovered five MAGs from this clade from the Auka sediments, ranging from 55 to 92% estimated completeness, with estimated contamination ranging from 0 to 4.3%. Combined with three MAGs from Guaymas Basin (completeness 55–70%, contamination 1.6–7.1%) these eight MAGs formed a deep-branching monophyletic clade related to the Desulfobacterota that we selected for further analysis. The size of the eight MAGs ranged from 1.3 to 3.1 Mbp and, using read mapping as a proxy for abundance, these organisms are enriched in the surface horizons of cores taken at both Auka and Guaymas. Their predicted OGT values (35–41 °C) are consistent with a niche in a mesophilic environment. In accordance with the recent proposal to use genomic information as type material for naming microbial taxa [89], we propose “*Candidatus* Tharpella aukensis*”* for the organism represented by the PB_MBMC_085 MAG (82% estimated completeness, 0% estimated contamination). As these organisms likely represent a novel phylum, we will refer to the clade containing these eight genomes as Tharpellota (Supplementary Data S3). The genus name *Tharpella* was chosen in honor of Marie Tharp, for her work on ocean floor mapping and plate tectonics that is key to our understanding of hydrothermal vents, where these organisms are found. The species name *aukensis* represents Auka, the location the MAG for which the name is proposed was recovered. Auka is in turn named after the shared word for “Hello” in several languages (Cochimi, Ipai, Paipai, and Tipai) of Indigenous people of the Baja California peninsula.

**Fig. 6:**
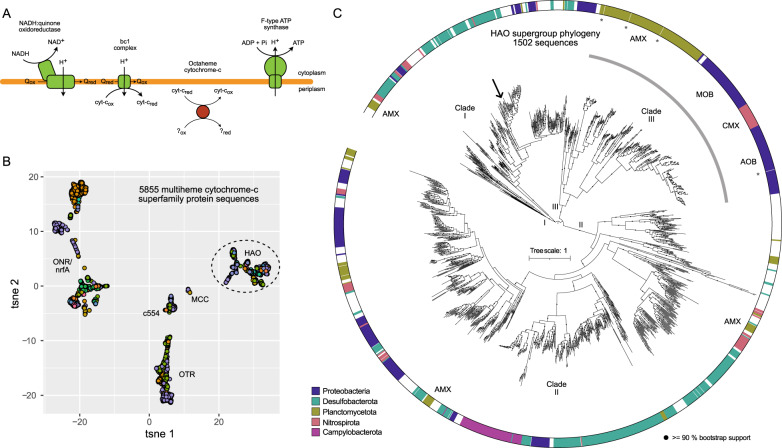
HAO family protein as proposed candidates for reduction of the terminal electron acceptor in Tharpellota clade. **A** Schematic overview of components of the electron transport chain detected in Tharpellota genomes, with the octaheme cytochrome c proposed to be involved in terminal electron reduction in Tharpellota indicated in red. **B** t-distributed stochastic neighborhood embedding (tSNE) representation of alignment score matrix of 5855 proteins of the HAO/OTR/ONR/nrfA/MCC/c554 structural fold family. Each point represents a protein sequence, colored by taxonomic affiliation at the phylum level. Dashed ellipse indicates sequences included in the phylogeny in **C**. HAO hydroxylamine oxidoreductase, OTR octaheme tetrathionate reductase, ONR octaheme nitrite reductase, nrfA pentaheme nitrite reductase, MCC octaheme sulfite reductase, c554 tetraheme cytochrome c554. **C** Approximate maximum likelihood phylogeny of 1502 HAO family protein sequences. The outer ring indicates taxonomic affiliation of the 5 most represented phyla, white space indicates other phyla. Clade designation (I, II, III) on the inside and outside of the tree reflects the clades identified by Klotz et al., 2008 (see text). Sequences known to be involved in nitrogen cycling are indicated with a gray arc. Discussed Tharpellota clade sequences are indicated with an arrow, sequences of structures included in the promals3D alignment are indicated with asterisks, known clades involved in nitrogen cycling are highlighted with 3 letter abbreviations. The function of several HAO family members in anammox Planctomycetota is unknown. AOB ammonia oxidizing bacteria, MOB methane oxidizing bacteria, CMX comammox Nitrospirota, AMX anammox Planctomycetota.

We performed functional enrichment analysis [90] on the 427 genomes within the Desulfobacterota obtained from Auka, Guaymas Basin and GTDB version 89 to investigate the metabolic potential of the Tharpellota (Supplementary Fig. S44). This analysis indicated the Tharpellota MAGs encode the potential for beta oxidation of long chain fatty acids and degradation of benzoyl-CoA, indicating a possible role in aromatic hydrocarbon degradation [91]. Furthermore, Tharpellota are likely capable of degradation of butyrate, as recently reported for *Ca. Phosphitivorax* sp. in the UBA1062 order [92], another deep-branching group within the Desulfobacterota. Unlike the UBA1062 genomes, Tharpellota MAGs encode NADH dehydrogenase (complex I), bc_1_-complex (complex III), and a heme biosynthesis pathway. We propose that Tharpellota MAGs use the electrons obtained from the oxidation of fatty acids and other hydrocarbons, and subsequently directed into the quinone pool via complex I to reduce a periplasmic electron acceptor (Fig. 6). However, the nature of this electron acceptor was not directly obvious from the genomic potential of the Tharpellota MAGs. Unlike many Desulfobacterota, the Tharpellota MAGs do not encode the capability to use sulfate as electron acceptor, and neither the functional enrichment analysis nor a follow up manual investigation of the genomes revealed terminal reductases for utilization of common electron acceptors.

While it is possible that genes for common terminal reductases are missing from the MAGs due to assembly and binning errors, their absence from all eight genomes is striking. Therefore, we screened for other potential terminal reductases among the Tharpellota genomes by analyzing genes containing heme binding motifs (CxxCH), as hemes can be involved in both electron transfer and catalytic centers. A protein affiliated with the multiheme cytochrome-*c* (MCC) fold family, harboring well-characterized proteins involved in nitrogen and sulfur cycling [93], was present in 7 of the 8 MAGs. An exploratory analysis of all 5855 MCC family proteins retrieved from the GTDB genomes (version 95) indicated this protein of interest was a member of the hydroxylamine oxidoreductase (HAO) family (Fig. 6). Proteins of the HAO family are known to play key roles in the nitrogen cycle, first identified as an essential protein in aerobic ammonia oxidizing bacteria (AOB) [94], and later shown to be essential for anaerobic ammonium oxidation (anammox) as well [95]. HAO family proteins from Campylobacterota have been shown to catalyze nitrite reduction to ammonium in vitro, although their physiological role remains unclear [96]. Notably, none of the HAO family proteins have suggested roles outside of the nitrogen cycle. However, our analysis found members of the HAO protein family in the genomes of many organisms not associated with nitrogen cycling, spanning 65 phyla (GTDB version 95), and most commonly in the Desulfobacterota phylum. Phylogenetic analysis of the 1502 HAO family proteins showed that those with a known function in aerobic and anaerobic ammonium oxidation form a monophyletic clade (Fig. 6). This branching pattern fits with previous work by Klotz et al., who divided the HAO family in three clades (I, II, and III), and showed that all HAOs with a confirmed function fall in clade III [97]. Our analysis includes a greater number of sequences, revealing intra clade diversity that was not previously shown. The Campylobacterota HAOs form a distinct lineage within clade II, while the remainder of clades I and II are composed of proteins of unknown function The Tharpellota proteins cluster within clade I within a group consisting of proteins from Desulfobacterota (Fig. 6). Based on this distribution, and the scarcity of oxidized nitrogen species in the Auka sediments (Supplementary Data S1), we hypothesize this protein catalyzes the reduction of sulfur species, rather than nitrogen. There is precedence for this as sulfur cycling has been shown for other members of the MCC fold family such as sulfite reductase mccA [98], and octaheme tetrathionate reductase [99]. We propose that this HAO family protein catalyzes the reduction of the terminal electron acceptor in the Tharpellota clade, based on its prevalence across the MAGs. We recognize this is a speculative hypothesis, and further investigations on the HAO family proteins outside clade III would be needed to substantiate it. Such experiments could include studies on cultured Desulfobacterota, heterologous expression of the Tharpellota sequences, or in vitro activity studies on Auka or Guaymas sediments.

It is also important to consider the possibility that individual Tharpellota MAGs use different electron acceptors, as there are several other protein complexes representing potential candidates as the site of the final reduction in the electron flow. Most prominently, several Tharpellota MAGs encode molybdopterin oxidoreductases, key complexes of many anaerobic metabolisms [100]. However, none of these molybdopterin oxidoreductase complexes have a known function, or are conserved in more than three of the eight genomes. In addition, there are several other cytochrome-containing proteins that could be candidate sites for terminal electron acceptor reduction in specific MAGs. Further research is needed to resolve the metabolism of this deep-branching Desulfobacterota clade, but this metagenomic analysis offers an entry point for further characterization of the closely related HAO family proteins from isolated organisms as well as designing targeted enrichments, transcriptomic analyses, or stable isotope probing experiments using vent samples harboring Tharpellota.

## Discussion

Genome-resolved metagenomics is rapidly yielding genomic information of organisms across the tree of life, which is valuable for hypotheses about the ecology and physiology of organisms in uncultured lineages, and to provide targets for subsequent experimental study. Our analyses of the Auka sediments revealed a highly diverse microbial community, comprising many understudied lineages affiliated with sediment-hosted hydrothermal vents. An in-depth look at one of these lineages, which we designated Tharpellota, revealed respiratory fatty acid degradation coupled to an unidentified electron acceptor, for which we propose reduction of a sulfur compound catalyzed by a MCC fold family protein. This provides a clear target for experimental verification, and confirmation of our hypothesis would have implications for interpretation of the biological sulfur cycle well beyond the Tharpellota.

In addition to gene function, investigating the genomic factors involved in colonization success at Auka and Guaymas is an exciting direction for future work, and a distinct advantage of using MAGs over the 16S rRNA gene amplicon analyses more frequently used for biogeography studies [85, 101, 102]. The geography and geological setting of the Gulf of California makes this region exceptionally well suited as a model system for hydrothermal vent biogeography. The narrow gulf constrains the currents and thus the direction of hydrothermal plumes, and the increasing sediment thickness with distance from the mouth of the gulf differentiates conditions between the two known vent sites. The spreading centers on the Carmen and Farallon segments, located between the Guaymas and Pescadero segments [15], could also harbor as yet undiscovered hydrothermal vents that act as stepping stones between the two sites.

Furthermore, the sediment-hosted hydrothermal vents of the Gulf of California provide an excellent study site for further discoveries of (hyper)thermophilic organisms. The maximum predicted OGT for organisms at Auka (111 °C) and Guaymas (117 °C) were substantially higher than the highest experimentally determined OGT values of 106 °C for *Pyrolobus fumarii* [103], and 105 °C for *Methanopyrus kandleri* grown at high pressure [10]. Maximum growth temperatures above 117 °C have been observed, but it should be noted that the model attempts to predict optimum growth temperatures. Interestingly, the predicted OGT for *M. kandleri* is 98 °C, identical to its observed optimum at ambient pressure [9], while the OGT prediction for *Pyrolobus fumarii* is 102 °C, slightly lower than the reported OGT. Strain 121, with an experimentally determined optimum of 103 °C [104], does not have a publicly available genome sequence; but the predicted OGT of 98 °C for *Pyrodictium abyssii*, the most closely related organism with a sequenced genome, is consistent with experimental observation [8]. The MAGs with predicted OGTs higher than the highest experimentally observed OGTs are not restricted to a single lineage, but rather distributed over several clades in the Thermoproteota (formerly Crenarchaeota). We note that the values discussed above are predictions, and should be treated as such. However, the Thermoproteota are severely undersampled, with the MAGs from Auka and Guaymas combined representing 40% of the Thermoproteota genomes analyzed in this study. This indicates further sampling will likely yield organisms with even higher predicted OGTs than those predicted here, and suggests the upper temperature limit of life could be considerably higher than currently known. Both in situ and in vitro experiments on the sediments of the Gulf of California hydrothermal vent sites could push our knowledge of the upper temperature boundary of life.

Finally, OGT prediction and genome-resolved metagenomics provide a powerful combination to examine the evolutionary history of thermophily. Our analysis shows that thermophily has evolved frequently in both the Bacteria and the Archaea (Supplementary Figs. S34–S47), suggesting adaptation of mesophiles to the colonization of high temperature environments. The rapidly increasing genomic representation of lineages in the tree of life makes phylogenomics combined with optimal growth temperature predictions an exciting angle on the debate about the conditions in which life on earth arose and diversified.

## Etymology

### Description of “*Candidatus* Tharpella” gen. nov

“*Candidatus* Tharpella” (N. L. fem. n. Tharpella, named after Marie Tharp (1920–2006), American geologist who pioneered systematic mapping seafloor and whose work was instrumental for the understanding of plate tectonics, and ultimately, the discovery of hydrothermal vents). A bacterial genus identified by metagenomic analyses and delineated according to Relative Evolutionary Distance by the Genome Taxonomy Database (GTDB). The type species of the genus is “*Candidatus* Tharpella aukensis”.

### Description of “*Candidatus* Tharpella aukensis” sp. nov

“*Candidatus* Tharpella aukensis” (L. fem. adj. aukensis, pertaining to the Auka vent field, where this species was discovered). A bacterial species identified by metagenomic analyses. This species includes all bacteria with genomes that show ≥95% average nucleotide identity to the type genome for the species to which we have assigned the MAG ID PB_MBMC_085 and which is available via NCBI BioSample SAMN18353627 and NCBI GenBank accession GCA_021163445.1.

### Description of “*Candidatus* Tharpellaceae” fam. nov

“*Candidatus* Tharpellaceae” (Tharp.el.la.ce'ae. N.L. fem. n. Tharpella. type genus of the family; N.L. suff. –aceae to denote a family; N.L. fem. pl. n. Tharpellaceae, the family of the genus Tharpella).

The description of the family “*Candidatus* Tharpellaceae” is the same as that of the genus “*Candidatus* Tharpella”. The type genus is “*Candidatus* Tharpella”.

### Description of “*Candidatus* Tharpellales” ord. nov

“*Candidatus* Tharpellales” (Tharp.el.la.les. N.L. fem. n. Tharpella. type genus of the order; N.L. suff. –ales to denote an order; N.L. fem. pl. n. Tharpellales, the order of the genus Tharpella)

The description of the order “*Candidatus* Tharpellales” is the same as that of the genus “*Candidatus* Tharpella”. The type family is “*Candidatus* Tharpellaceae”.

### Description of “*Candidatus* Tharpellia” class. nov

“*Candidatus* Tharpellia” (Tharp.el.li.a. N.L. fem. n. Tharpella. type genus of the class; N.L. suff. –ia to denote a class; N.L. fem. pl. n. Tharpellia, the class of the genus Tharpella).

The description of the class “*Candidatus* Tharpellia” is the same as that of the genus “*Candidatus* Tharpella”. The type order is “*Candidatus* Tharpellales”.

### Description of “*Candidatus* Tharpellota” phylum. nov

“*Candidatus* Tharpellota” (Tharp.el.lo’ta N.L. fem. n. Tharpella. type genus of the phylum; N.L. suff. –ota to denote a phylum; N.L. fem. pl. n. Tharpellota, the phylum of the genus Tharpella).

The description of the phylum “*Candidatus* Tharpellota” is the same as that of the genus “*Candidatus* Tharpella”. The type class is “*Candidatus* Tharpellia”.

## Supplementary information

Supplemental Information

Supplemental Data S1

Supplemental Data S2

Supplemental Data S3

Supplemental Data S4

Supplemental Data S5

Supplemental Data S6

Supplemental Data S7

Supplemental Data S8
