# Supplementary material for: Microbial communities of Auka hydrothermal sediments shed light on vent biogeography and the evolutionary history of thermophily
Source: ISME J. 2022 Mar 28;16(7):1750–64. doi: 10.1038/s41396-022-01222-x (PMC9213671; doi:10.1038/s41396-022-01222-x)
Supplement: Supplementary file 1 — Supplemental Information [file 41396_2022_1222_MOESM1_ESM.pdf]

Supplemental information

This file contains supplemental information to "Microbial communities of Auka hydrothermal sediments shed light on vent biogeography and the evolutionary history of thermophily", by Speth *et al.* It contains a list of supplemental data files, a list of supplemental figures, 47 supplemental figures, and supplemental Text.

List of Supplemental data files:

Supplemental Data S1 - Processed major ion data for the Auka sediment cores
Supplemental Data S2 - Metadata for the 16S rRNA gene amplicon sequencing
Supplemental Data S3 - ASV abundance data in Auka sediment cores
Supplemental Data S4 - Fasta file with sequences for the 18777 ASVs
Supplemental Data S5 - Table with MAG metadata
Supplemental Data S6 - Annotations for all proteins in all MAGs
Supplemental Data S7 - Fasta file with all proteins in all MAGs
Supplemental Data S8 - Table with low completeness bin statistics

List of Supplemental Figures:

Supplemental Figure S1 - Environmental context of sediment pushcore samples
Supplemental Figure S2 - Major ions of eight sediment cores retrieved on NA091
Supplemental Figure S3 - Major ions of fourteen sediment cores retrieved on FK181031
Supplemental Figure S4 - ASV abundance by depth in core020
Supplemental Figure S5 - ASV abundance by depth in core048
Supplemental Figure S6 - ASV abundance by depth in core051
Supplemental Figure S7 - ASV abundance by depth in core087
Supplemental Figure S8 - ASV abundance by depth in core089
Supplemental Figure S9 - ASV abundance by depth in core111
Supplemental Figure S10 - ASV abundance by depth in core118
Supplemental Figure S11 - ASV abundance by depth in core119
Supplemental Figure S12 - ASV abundance by depth in S0193 PC1
Supplemental Figure S13 - ASV abundance by depth in S0193 PC2
Supplemental Figure S14 - ASV abundance by depth in S0193 PC3
Supplemental Figure S15 - ASV abundance by depth in S0193 PC5
Supplemental Figure S16 - ASV abundance by depth in S0193 PC7
Supplemental Figure S17 - ASV abundance by depth in S0194 PC0
Supplemental Figure S18 - ASV abundance by depth in S0194 PC1
Supplemental Figure S19 - ASV abundance by depth in S0194 PC2
Supplemental Figure S20 - ASV abundance by depth in S0194 PC3
Supplemental Figure S21 - ASV abundance by depth in S0194 PC4
Supplemental Figure S22 - ASV abundance by depth in S0196 PC1
Supplemental Figure S23 - ASV abundance by depth in S0196 PC5
Supplemental Figure S24 - ASV abundance by depth in S0196 PC6
Supplemental Figure S25 - ASV abundance by depth in S0196 PC7
Supplemental Figure S26 - ASV abundance by depth in S0196 PC8

Supplemental Figure S27 - ASV abundance by depth in S0198 PC1
Supplemental Figure S28 - ASV abundance by depth in S0198 PC3
Supplemental Figure S29 - ASV abundance by depth in S0198 PC5
Supplemental Figure S30 - ASV abundance by depth in S0200 PC1
Supplemental Figure S31 - ASV abundance by depth in S0200 PC5
Supplemental Figure S32 - ASV abundance by depth in S0200 PC7
Supplemental Figure S33 - 16S rRNA gene amplicon reads matching the metagenome
Supplemental Figure S34 - Phylogeny and OGT prediction of Crenarchaeota
Supplemental Figure S35 - Phylogeny and OGT prediction of DPANN superphylum
Supplemental Figure S36 - Phylogeny and OGT prediction of Euryarchaeota
Supplemental Figure S37 - Phylogeny and OGT prediction of Halobacterota
Supplemental Figure S38 - Phylogeny and OGT prediction of Thermoplasmatota
Supplemental Figure S39 - Phylogeny and OGT prediction of Acidobacteriota
Supplemental Figure S40 - Phylogeny and OGT prediction of phyla basal to Bacteroidota
Supplemental Figure S41 - Phylogeny and OGT prediction of Campylobacterota
Supplemental Figure S42 - Phylogeny and OGT prediction of Chloroflexota
Supplemental Figure S43 - Phylogeny and OGT prediction of CPR superphylum
Supplemental Figure S44 - Phylogeny and OGT prediction of Desulfobacterota
Supplemental Figure S45 - Phylogeny and OGT prediction of PVC superphylum
Supplemental Figure S46 - Phylogeny and OGT prediction of Spirochaetota
Supplemental Figure S47 - Phylogeny and OGT prediction of Thermoplasmatota

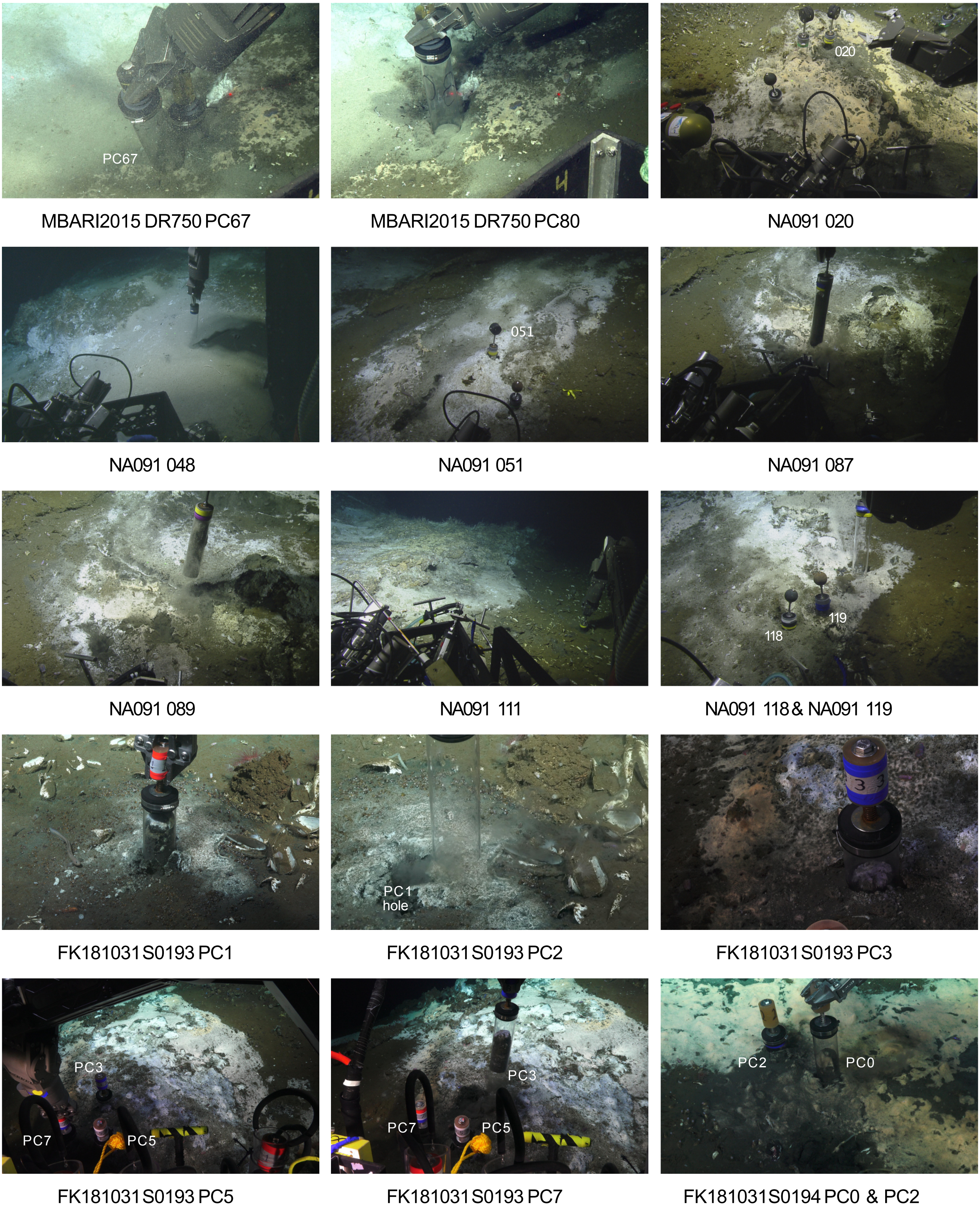

Supplemental figure S1. Overview of sediment environments sampled using push cores
Photos of sampling of the sediment push cores used in this study, sampled on RV Western Flyer using ROV Doc Ricketts (MBARI2015), EV Nautilus using ROV Hercules (NA091), and RV Falkor using ROV SuBastian (FK181031). In photos showing multiple cores, cores are labeled. Figure continues on the next page.

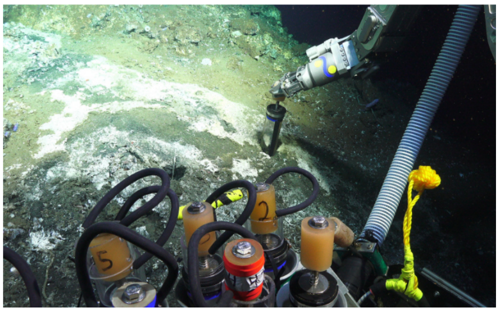

FK181031S0194 PC1

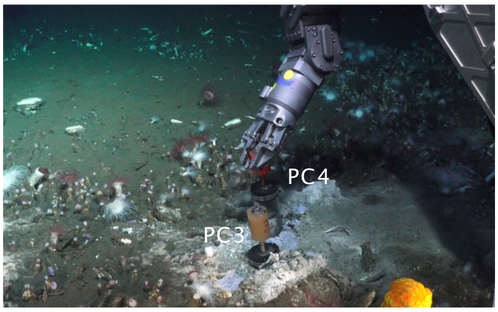

FK181031S0194 PC3 & PC4

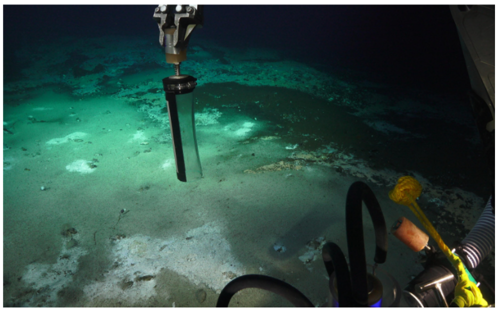

FK181031S0196 PC1

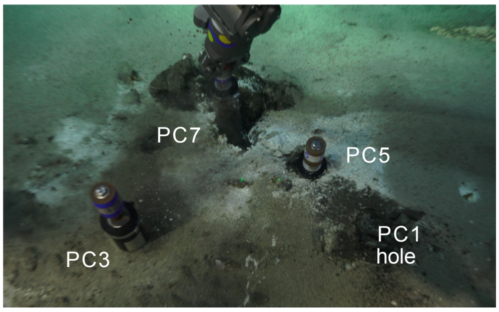

FK181031S0196 PC5 & PC7

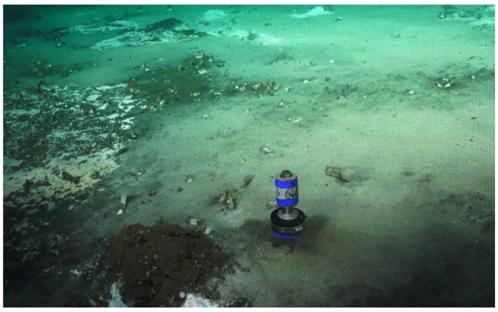

FK181031S0196 PC6

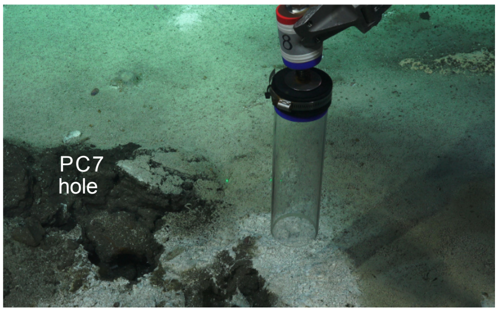

FK181031S0196 PC8

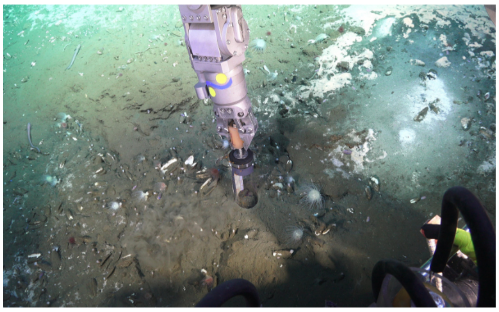

FK181031S0198 PC1

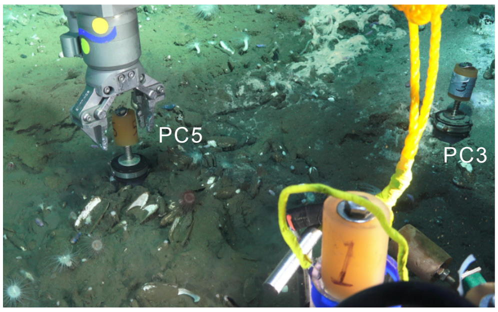

FK181031S0198 PC3 & PC5

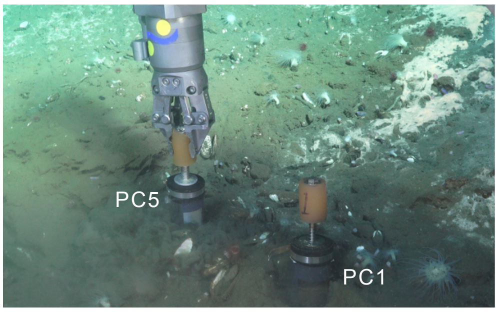

FK181031S0198 PC1 & PC5

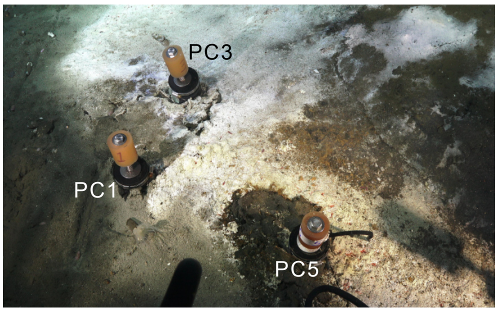

FK181031S0200 PC1, PC3, PC5

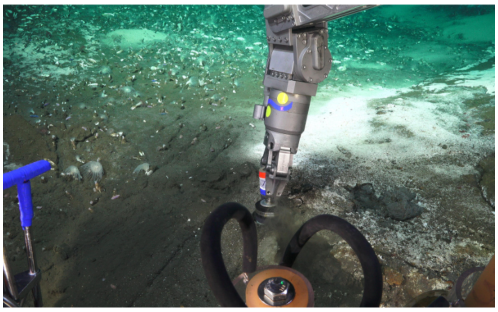

FK181031S0200 PC7

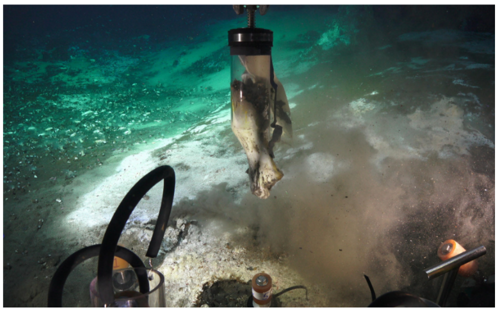

FK181031S0200 PC3

Supplemental figure S1. Overview of sediment environments sampled using push cores (cont.) Photos of sampling of the sediment push cores used in this study, sampled on RV Western Flyer using ROV Doc Ricketts (MBARI2015), EV Nautilus using ROV Hercules (NA091), and RV Falkor using ROV SuBastian (FK181031). In photos showing multiple cores, cores are labeled. Page 2 of 2.

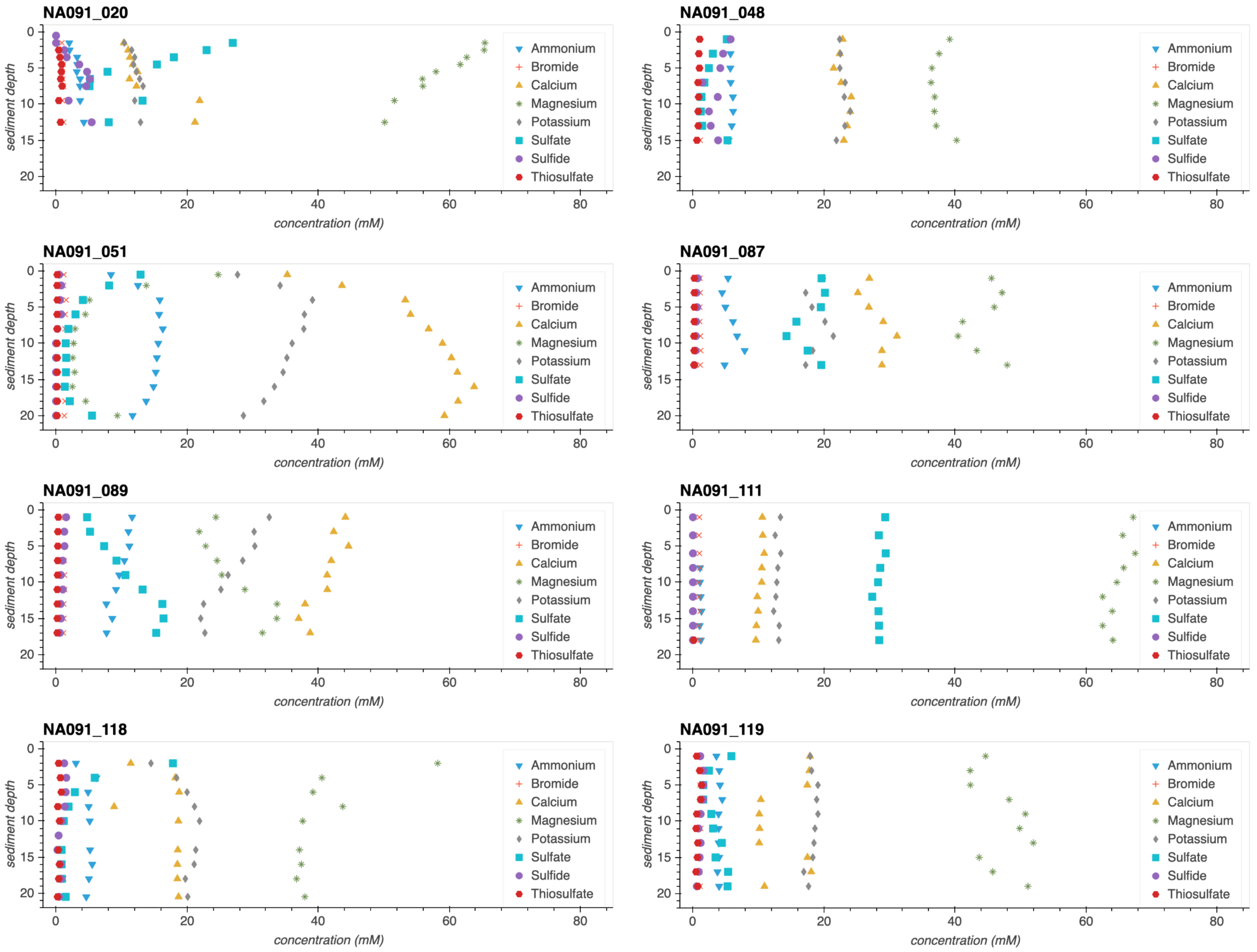

Supplemental figure S2. Major ions in the pushcore samples from cruise NA091
Concentrations of major ions as a function of depth in the sediments. Each point represents the porewater extracted from a sediment core horizon, plotted at the average depth of the horizon depth interval. All ions except for sulfide were measured using ion chromatography, and sulfide was measured colorimetrically.

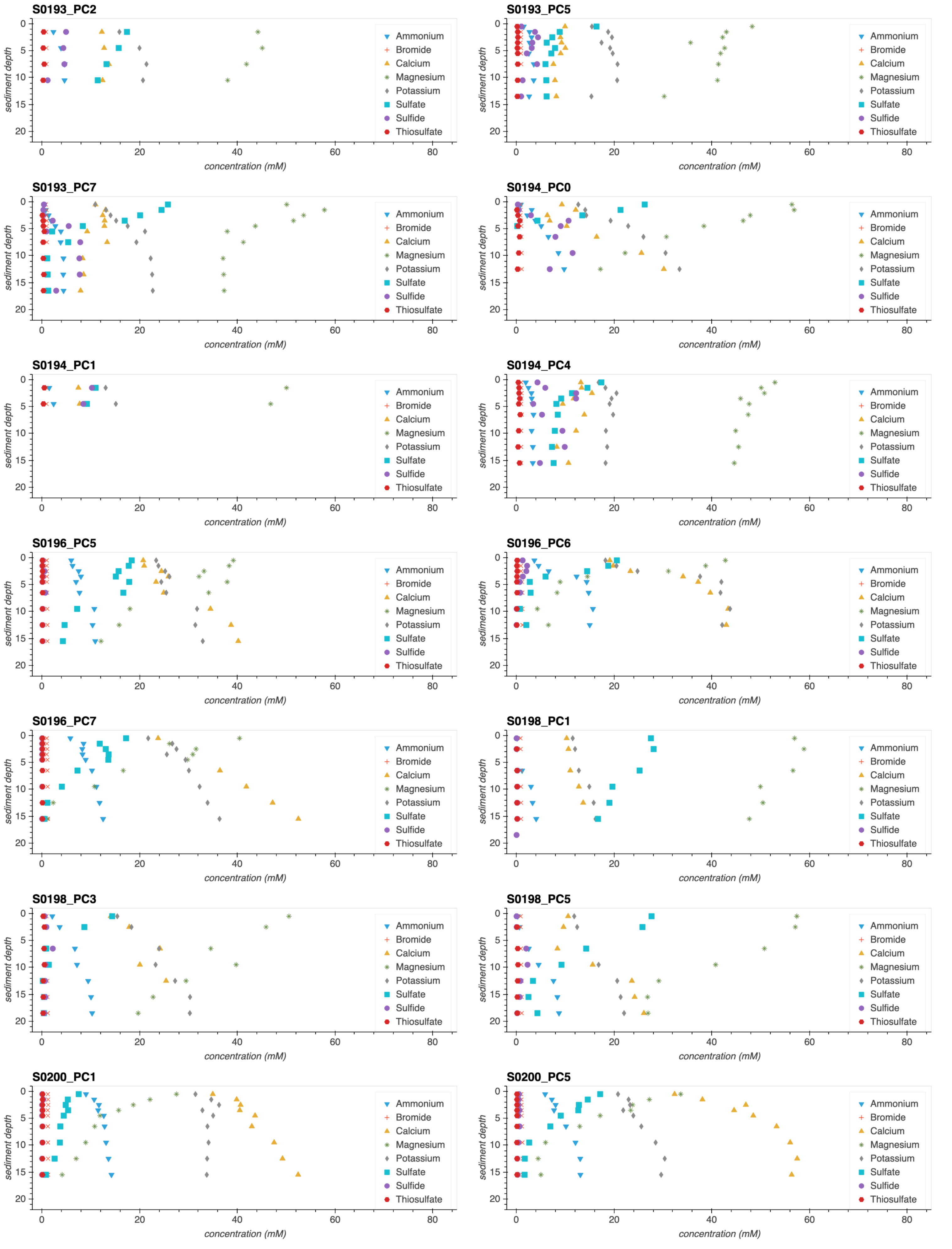

Supplemental figure S3. Major ions in the pushcore samples from cruise FK181031
Concentrations of major ions as a function of depth in the sediments. Each point represents the porewater extracted from a sediment core horizon, plotted at the average depth of the horizon depth interval. All ions except for sulfide were measured using ion chromatography, and sulfide was measured colorimetrically.

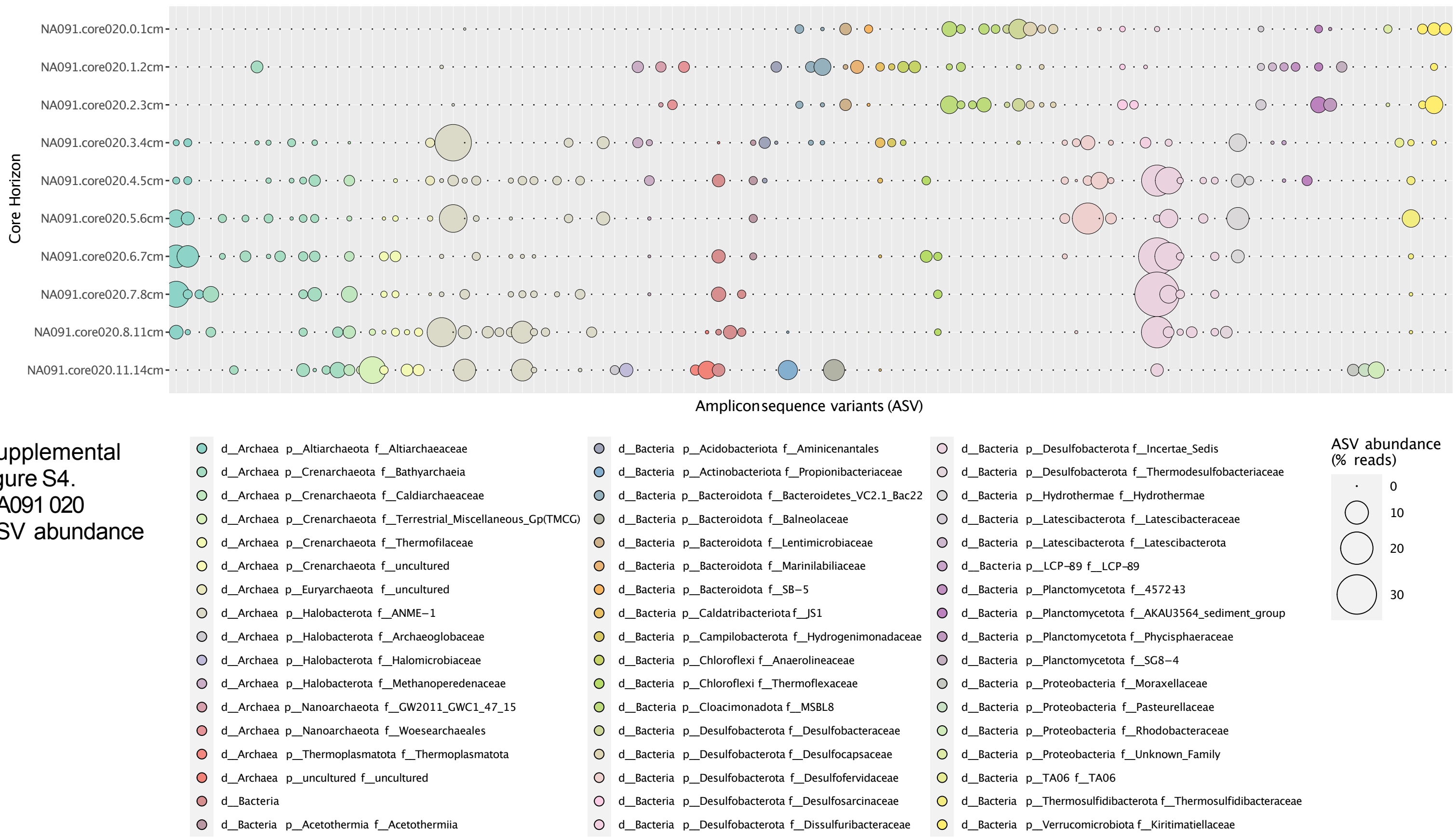

Supplemental figure S4. NA091 020 ASV abundance

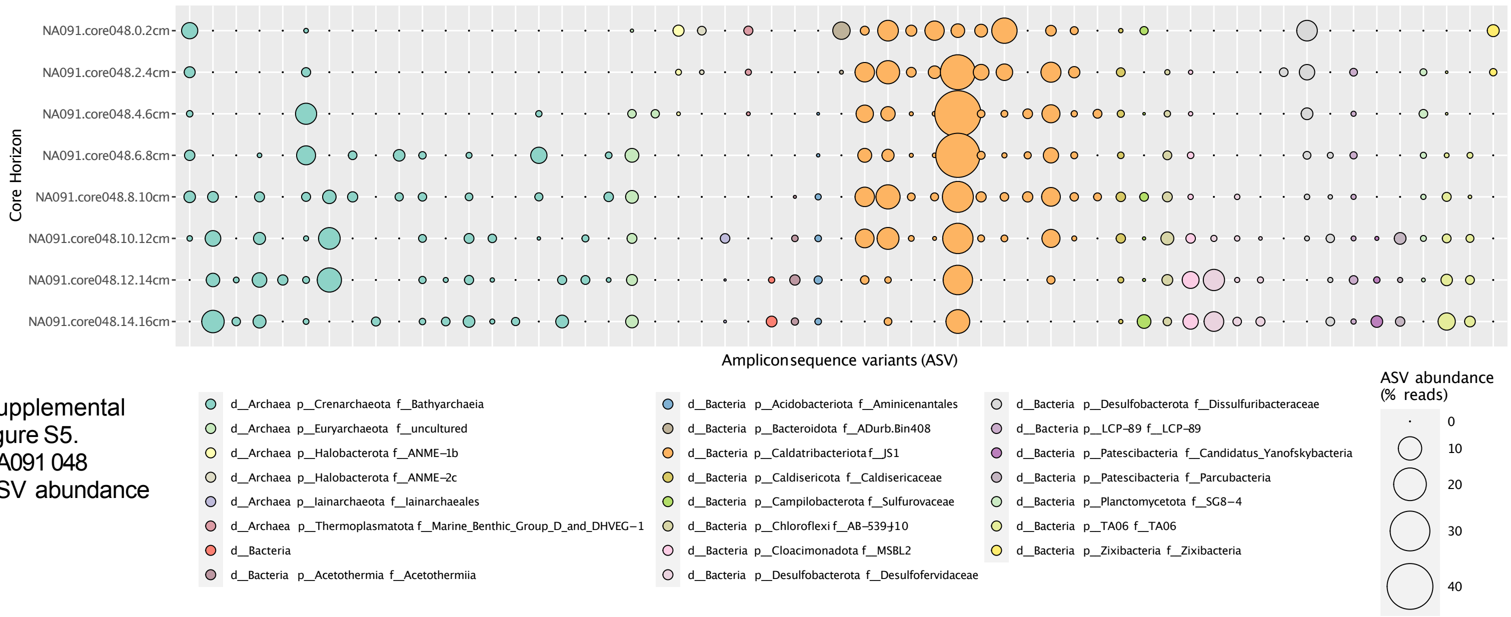

Supplemental figure S5. NA091 048 ASV abundance

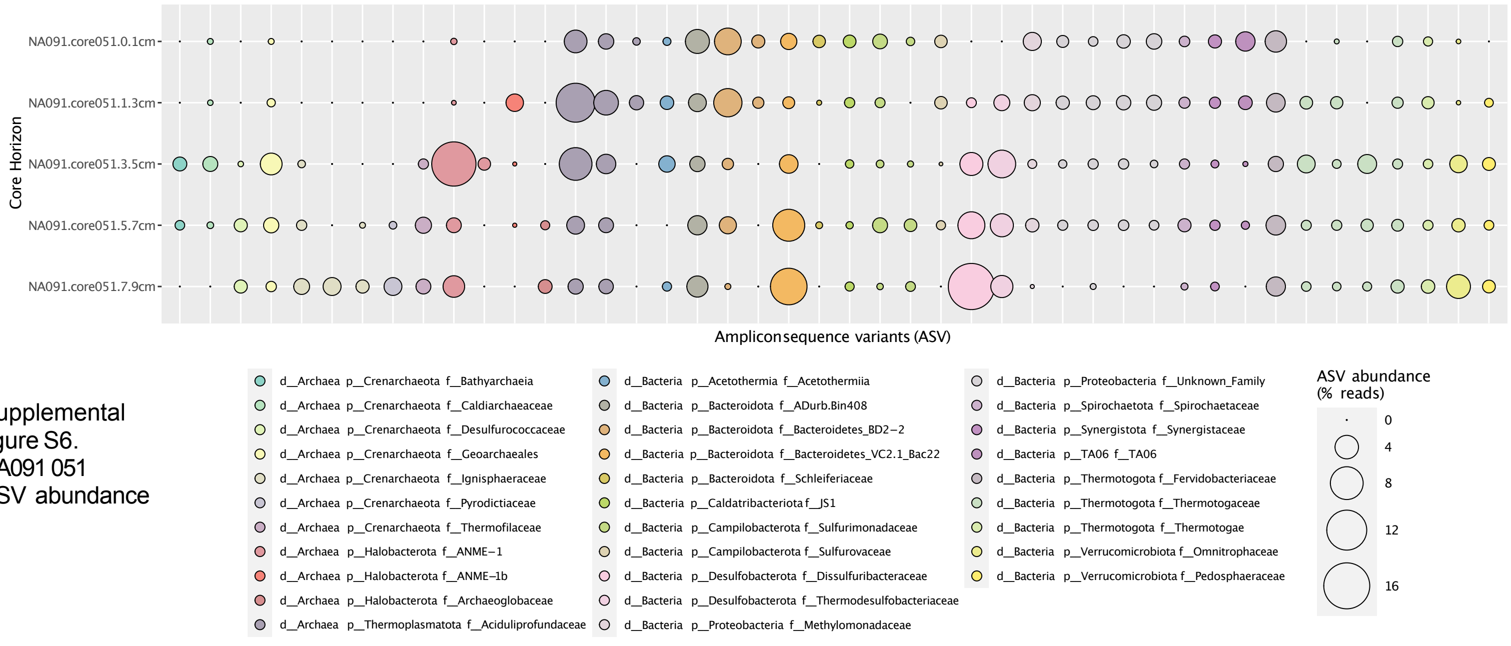

Supplemental figure S6. NA091 051 ASV abundance

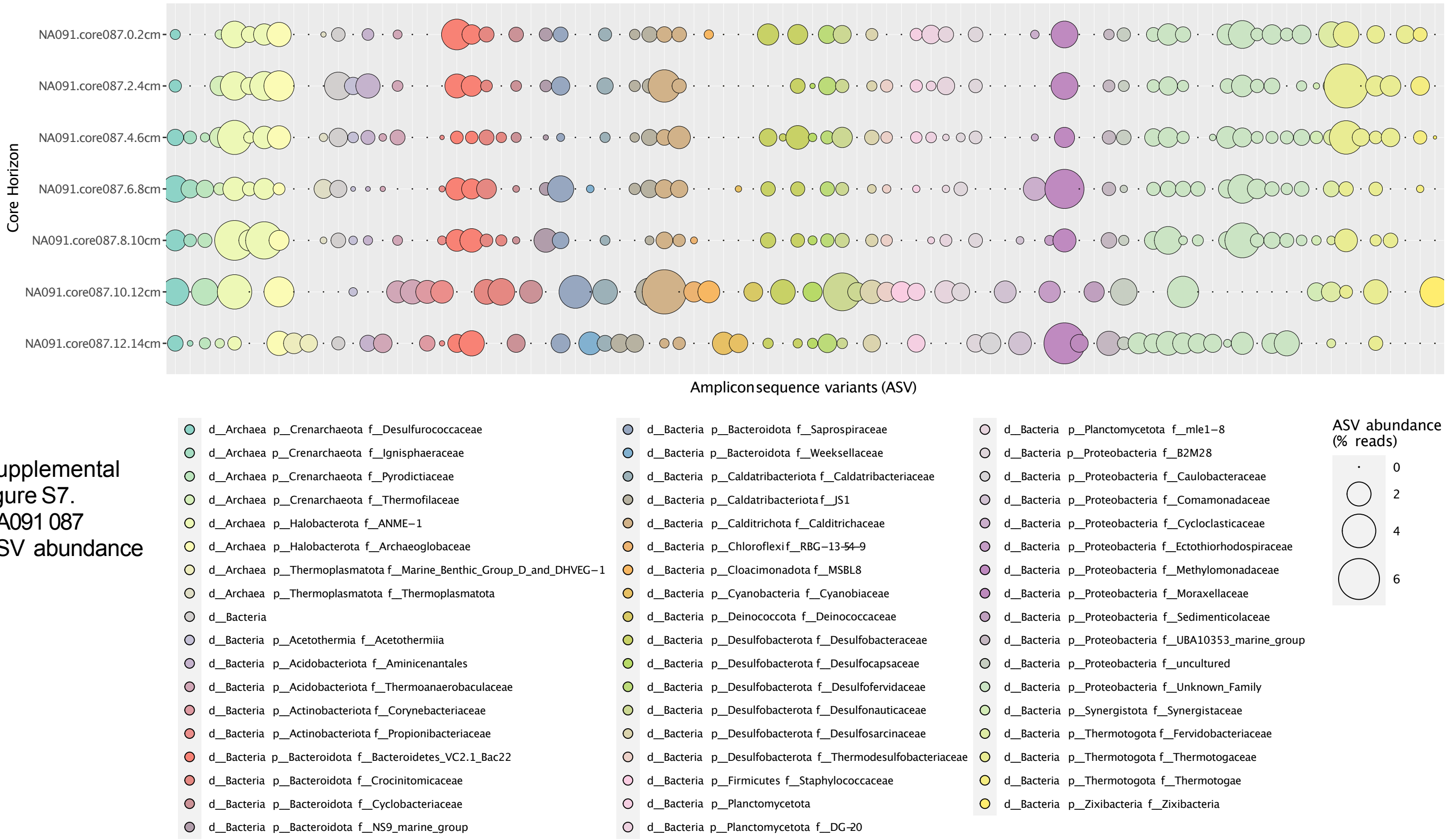

Supplemental figure S7. NA091 087 ASV abundance

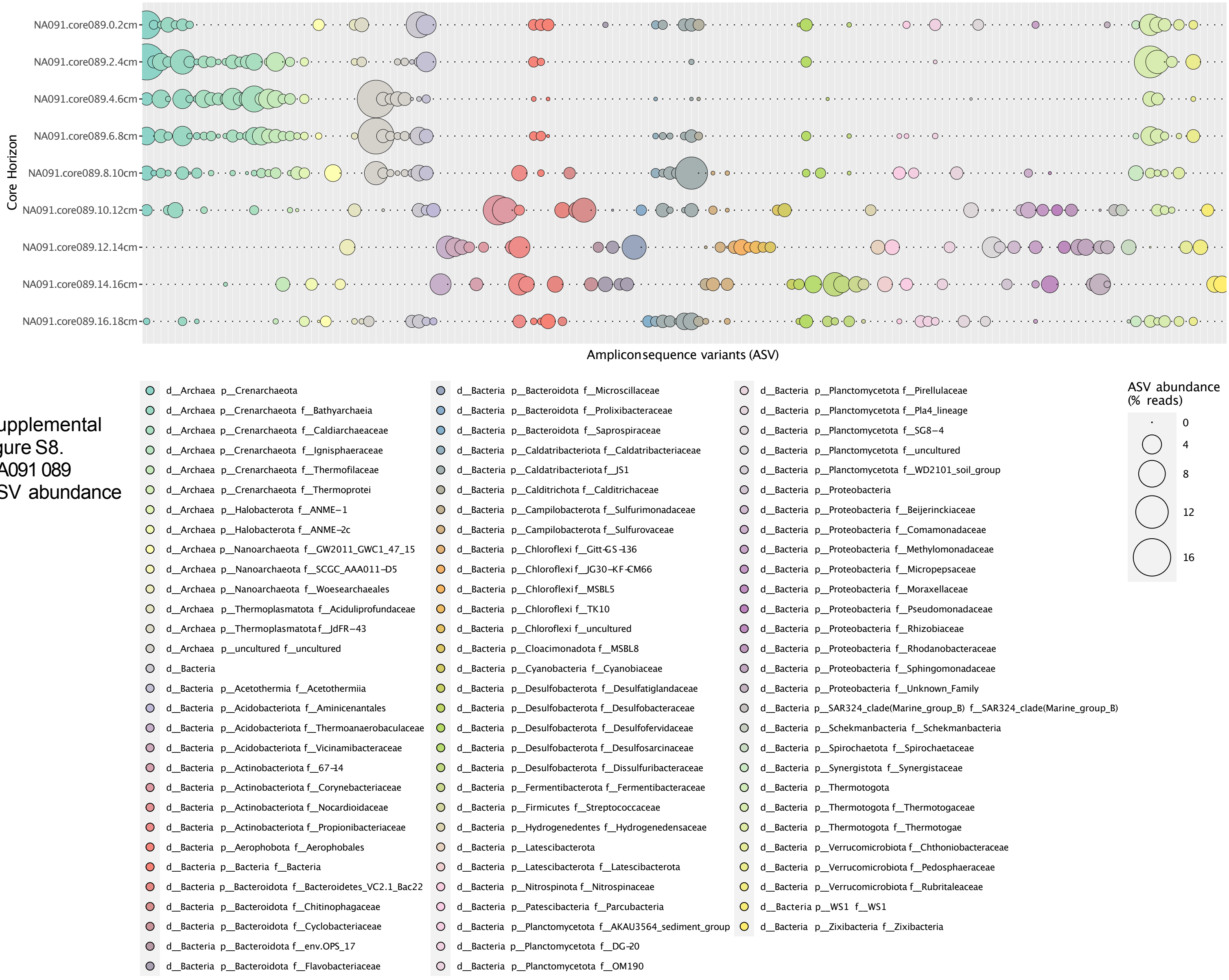

Supplemental figure S8. NA091 089 ASV abundance

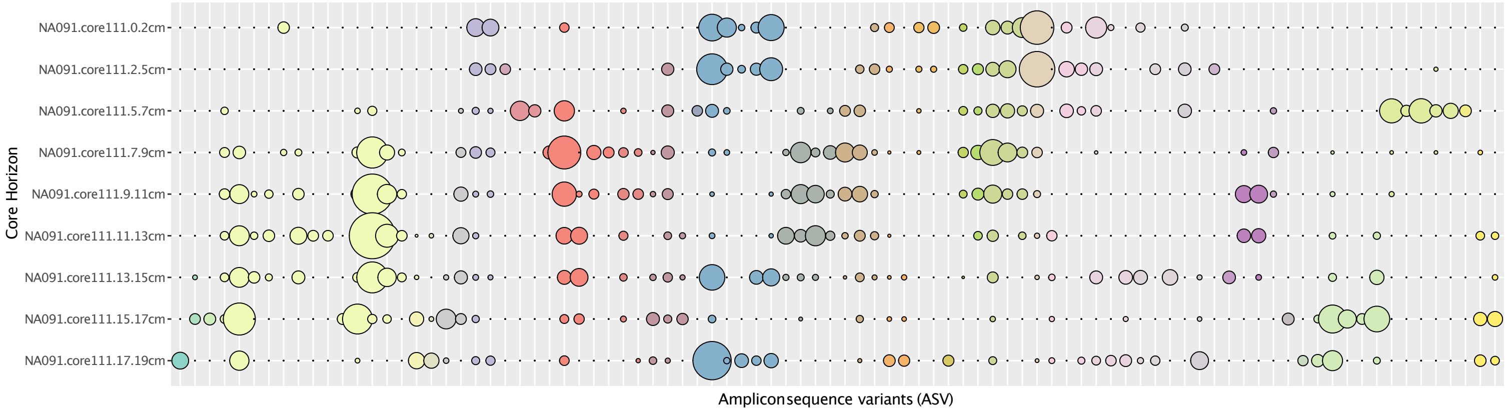

Supplemental figure S9. NA091 111 ASV abundance

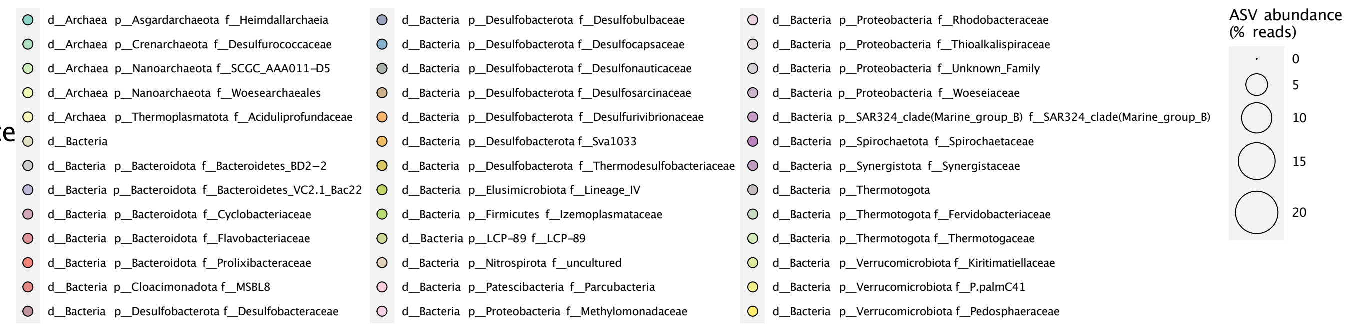

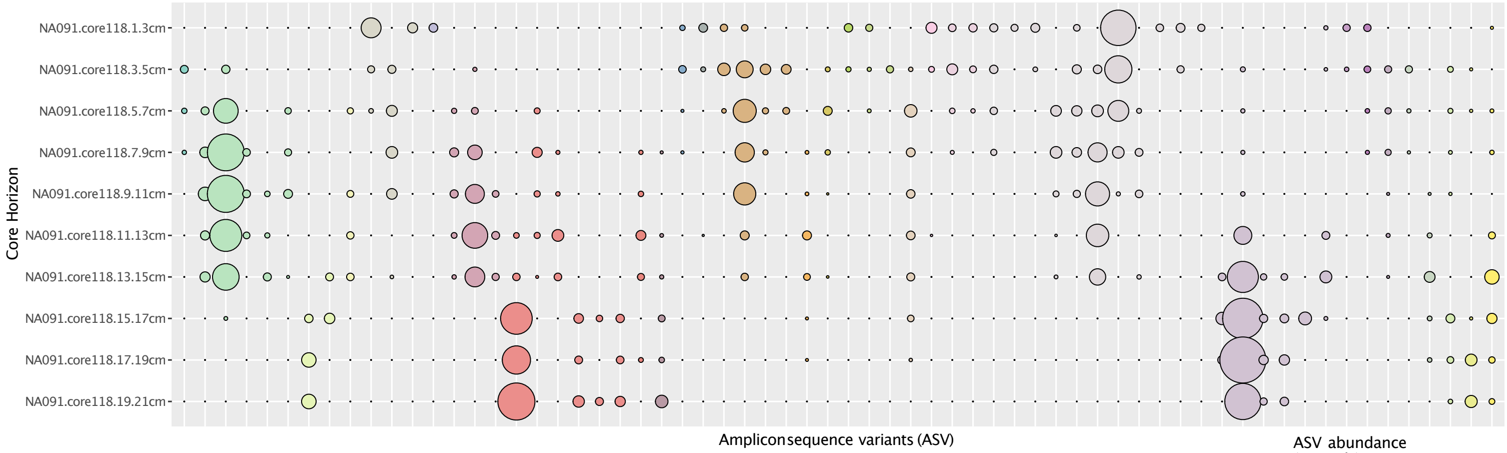

Supplemental figure S10. NA091 118 ASV abundance

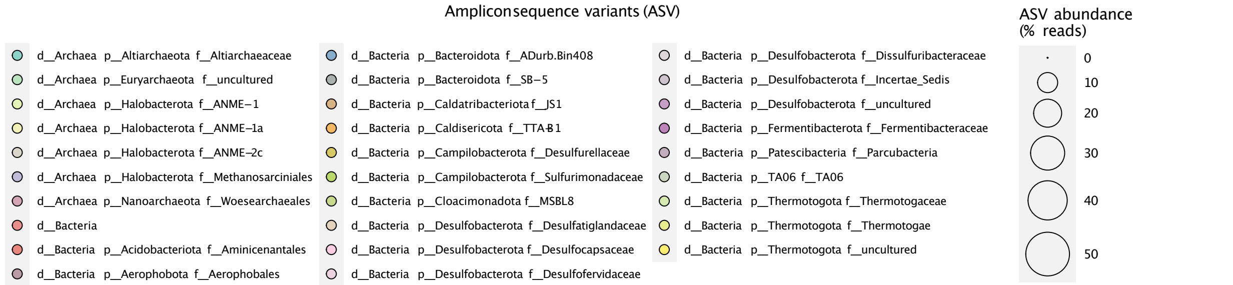

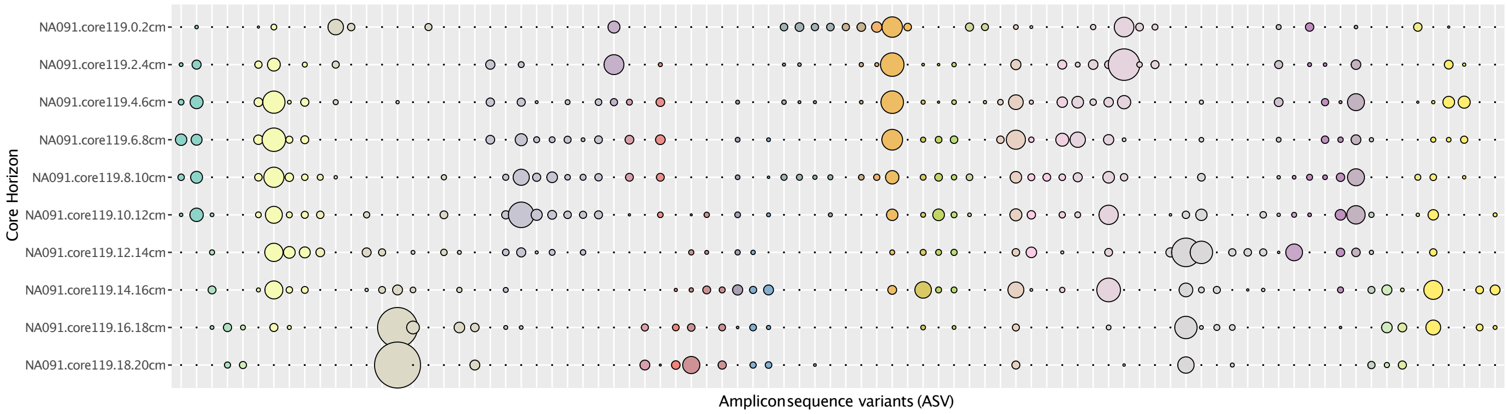

Supplemental figure S11. NA091 119 ASV abundance

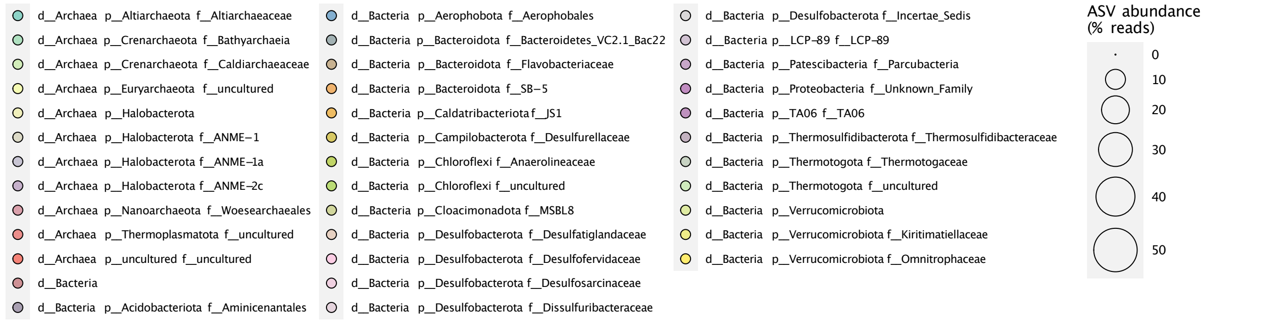

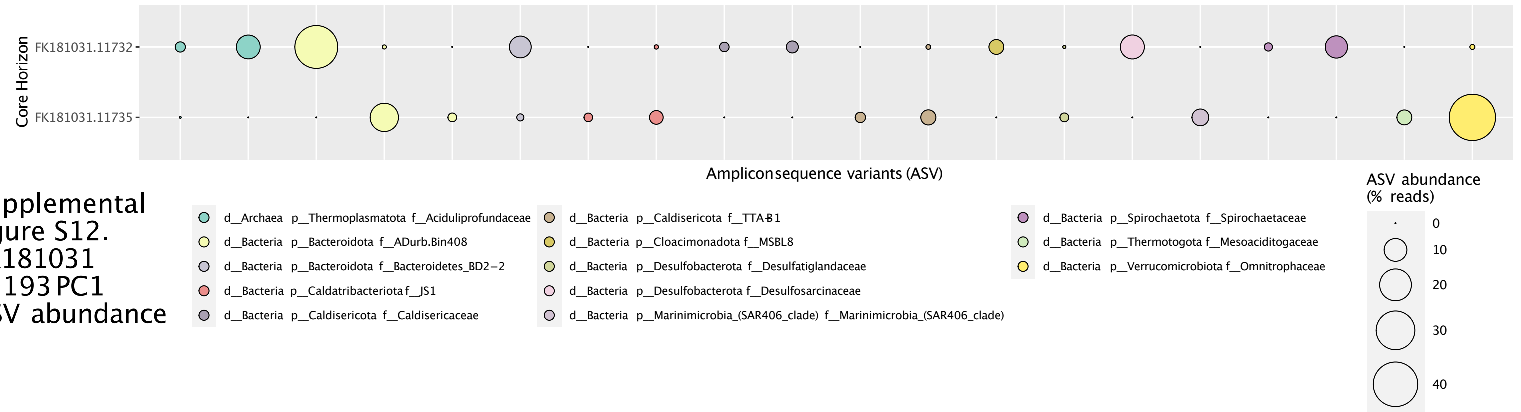

Supplemental figure S12. FK181031 S0193 PC1 ASV abundance

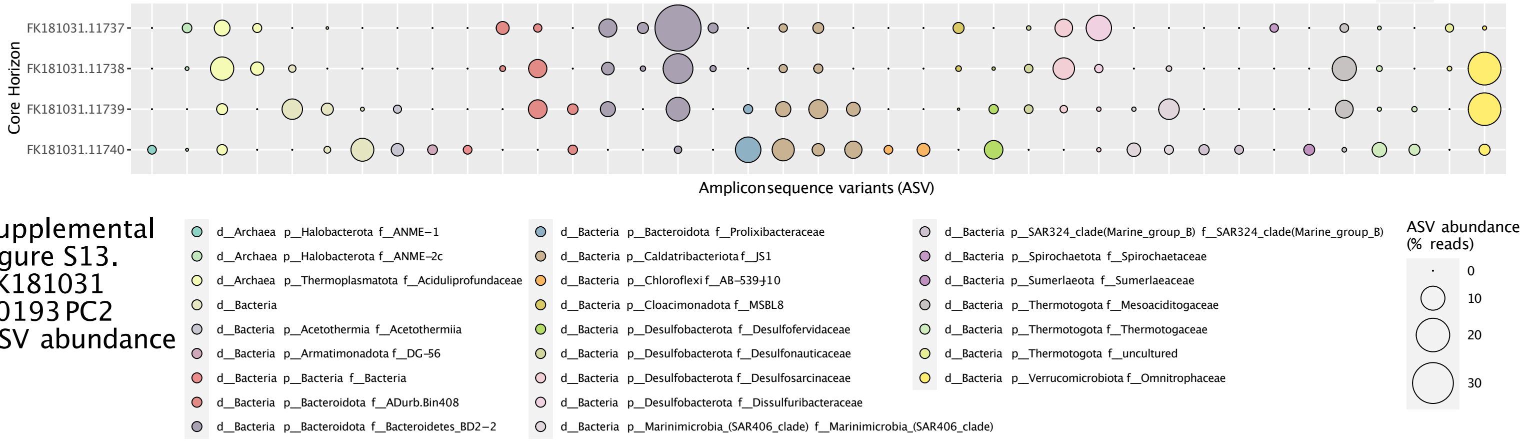

Supplemental figure S13. FK181031 S0193 PC2 ASV abundance

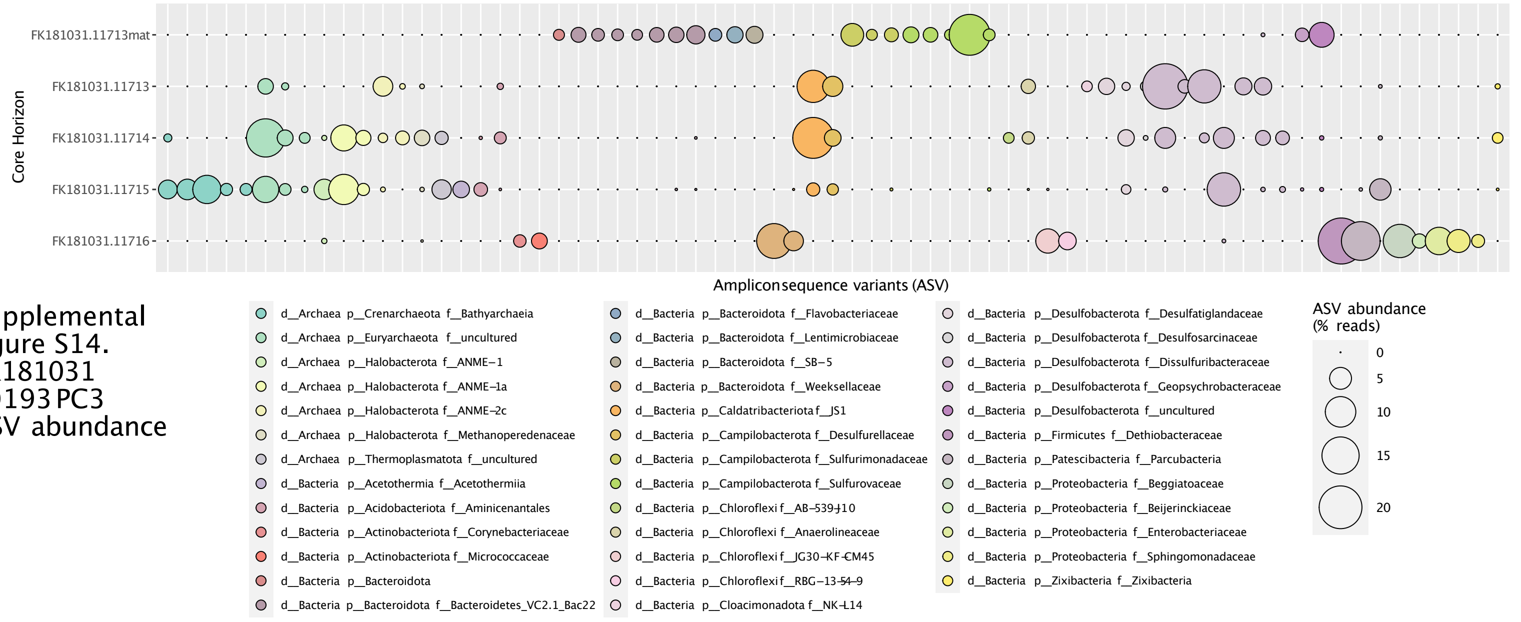

Supplemental figure S14. FK181031 S0193 PC3 ASV abundance

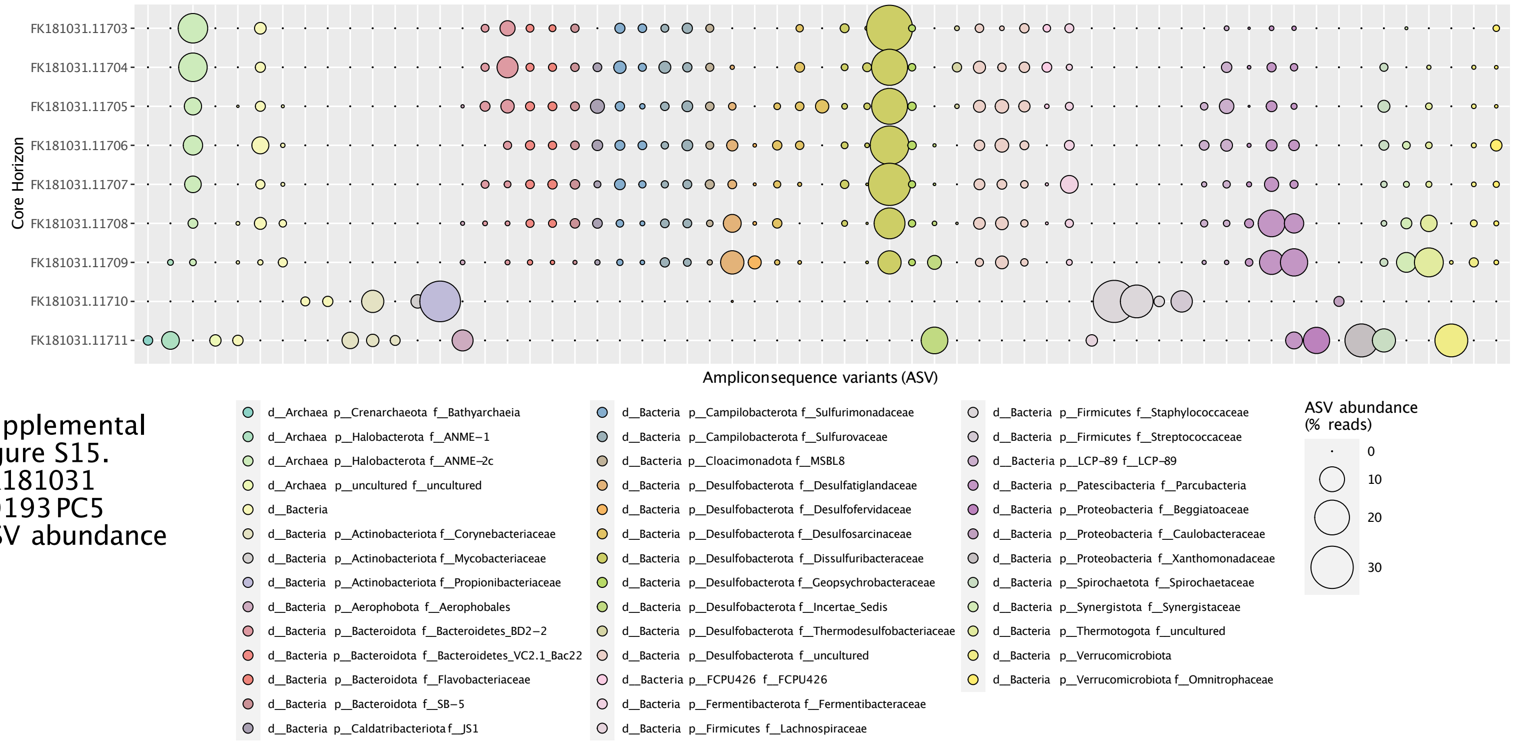

Supplemental figure S15. FK181031 S0193 PC5 ASV abundance

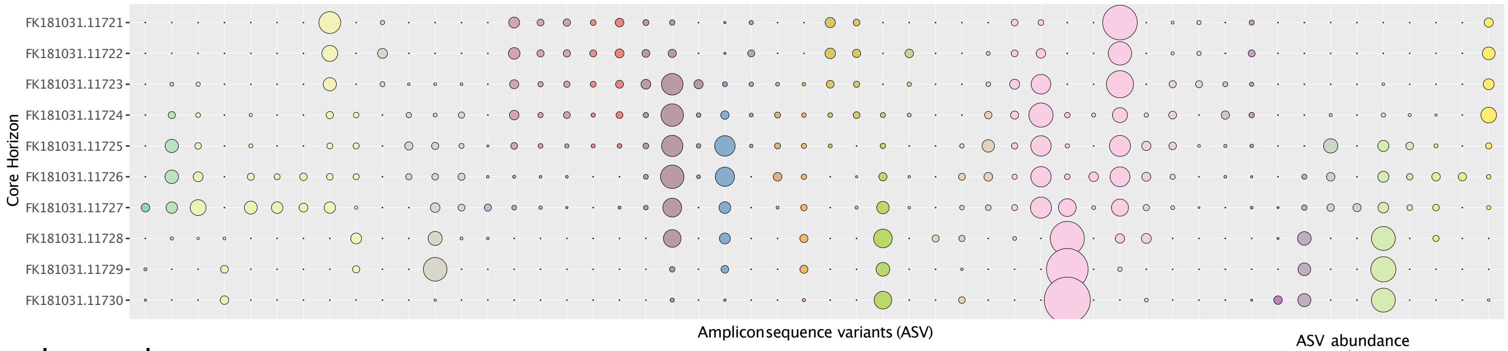

Supplemental figure S16. FK181031 S0193 PC7 ASV abundance

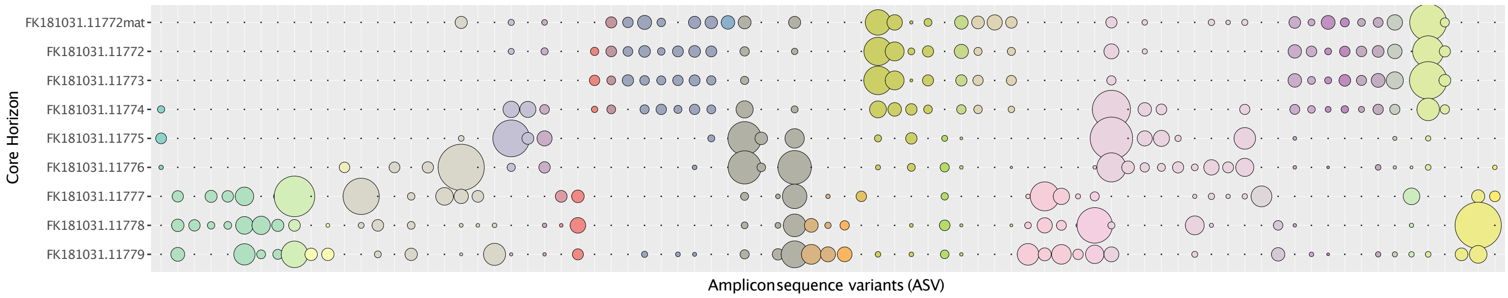

Supplemental figure S17. FK181031 S0194 PC0 ASV abundance

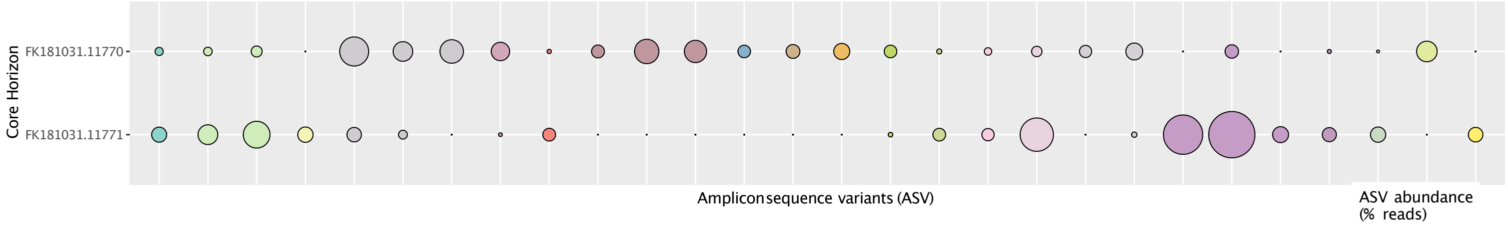

Supplemental figure S18. FK181031 S0194 PC1 ASV abundance

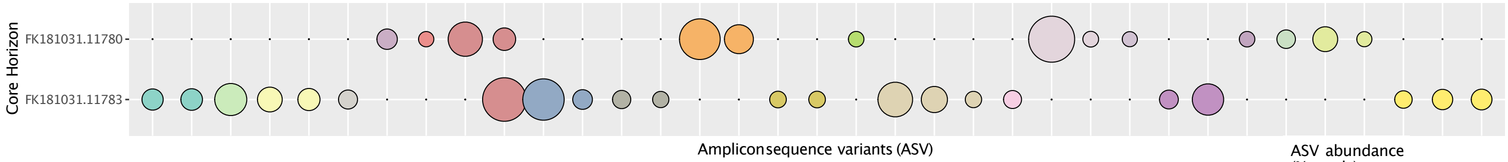

Supplemental figure S19. FK181031 S0194 PC2 ASV abundance

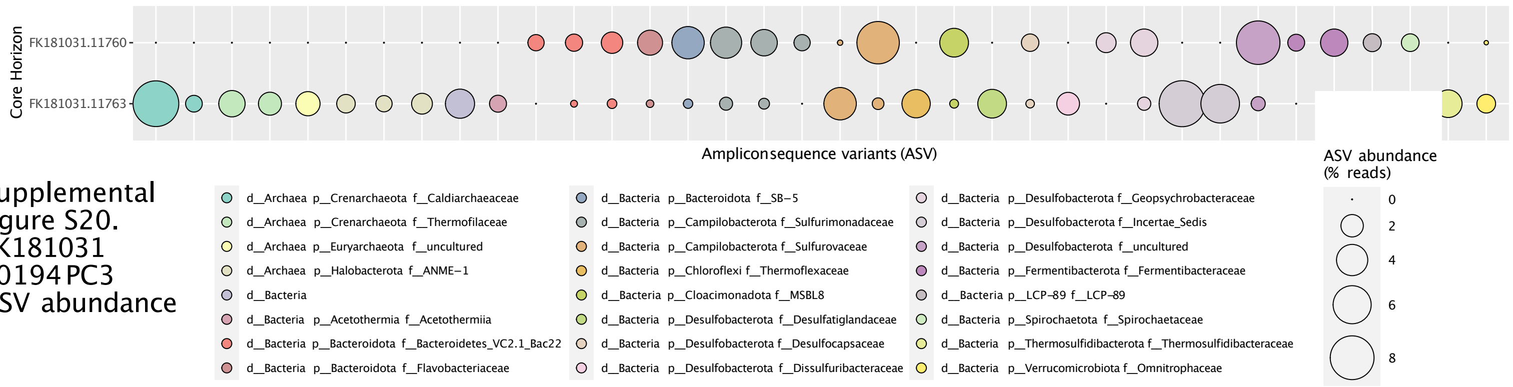

Supplemental figure S20. FK181031 S0194 PC3 ASV abundance

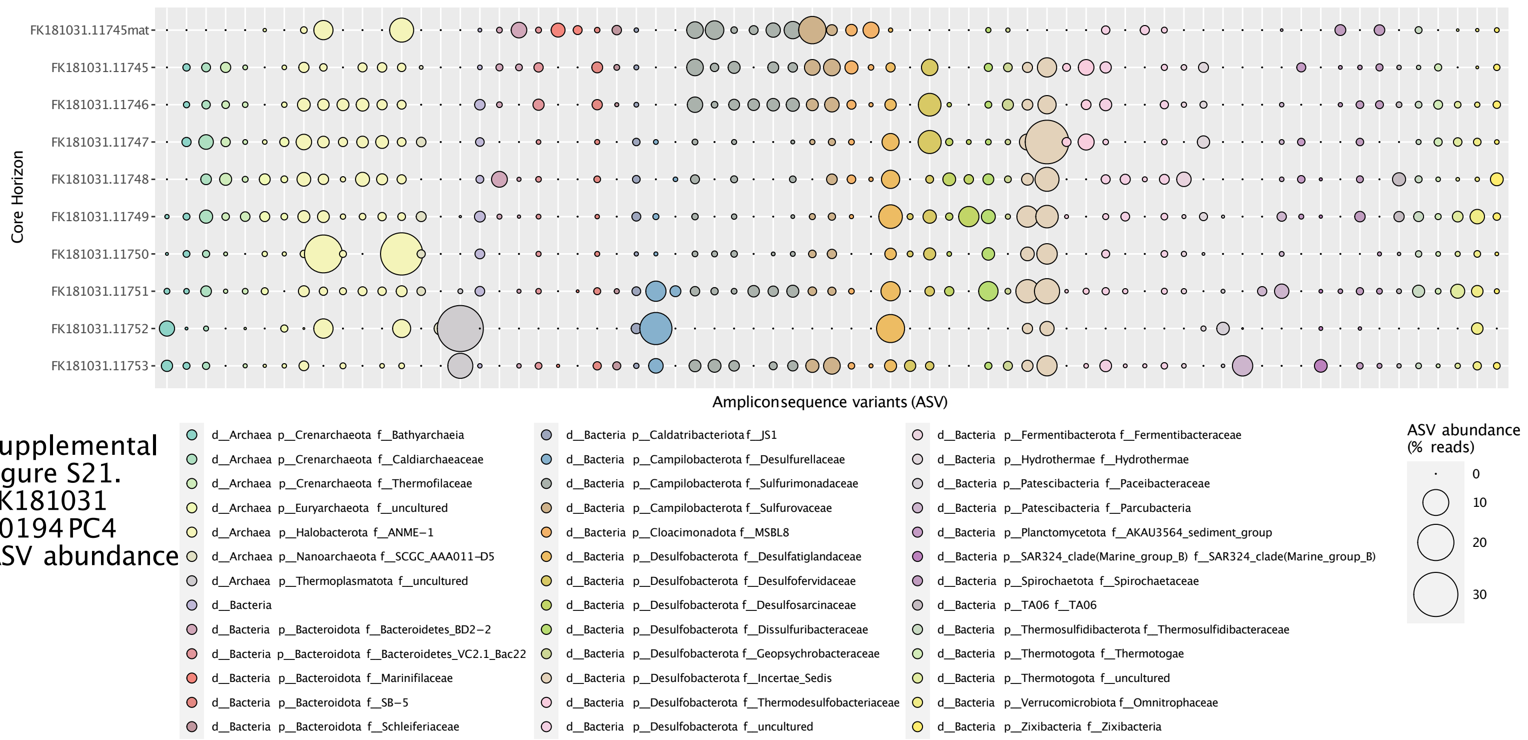

Supplemental figure S21. FK181031 S0194 PC4 ASV abundance

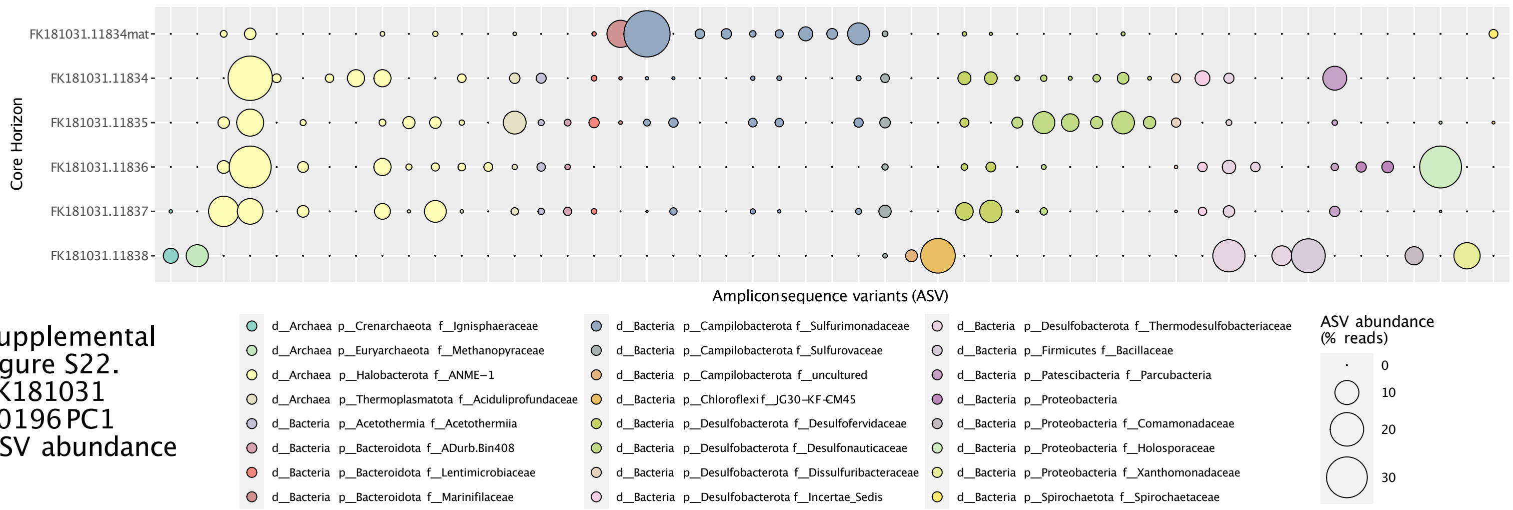

Supplemental figure S22. FK181031 S0196 PC1 ASV abundance

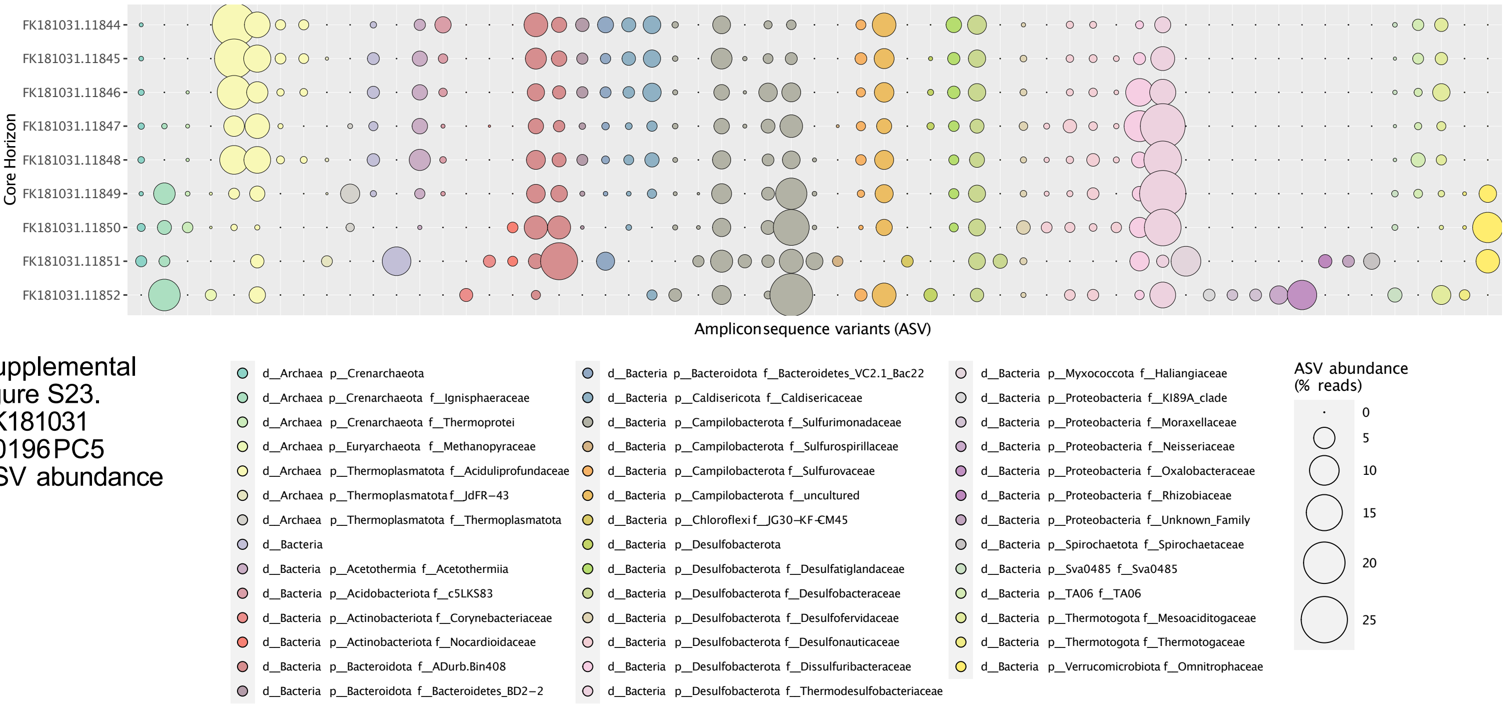

Supplemental figure S23. FK181031 S0196PC5 ASV abundance

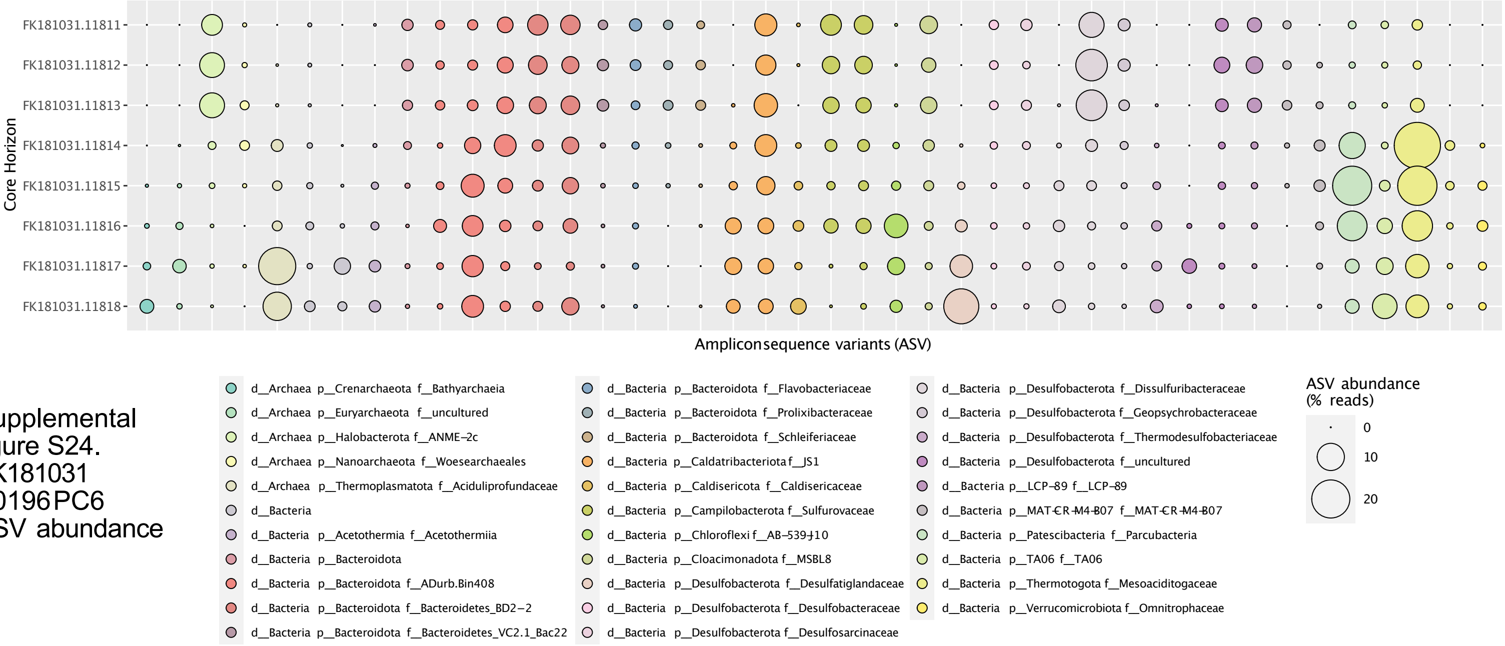

Supplemental figure S24. FK181031 S0196PC6 ASV abundance

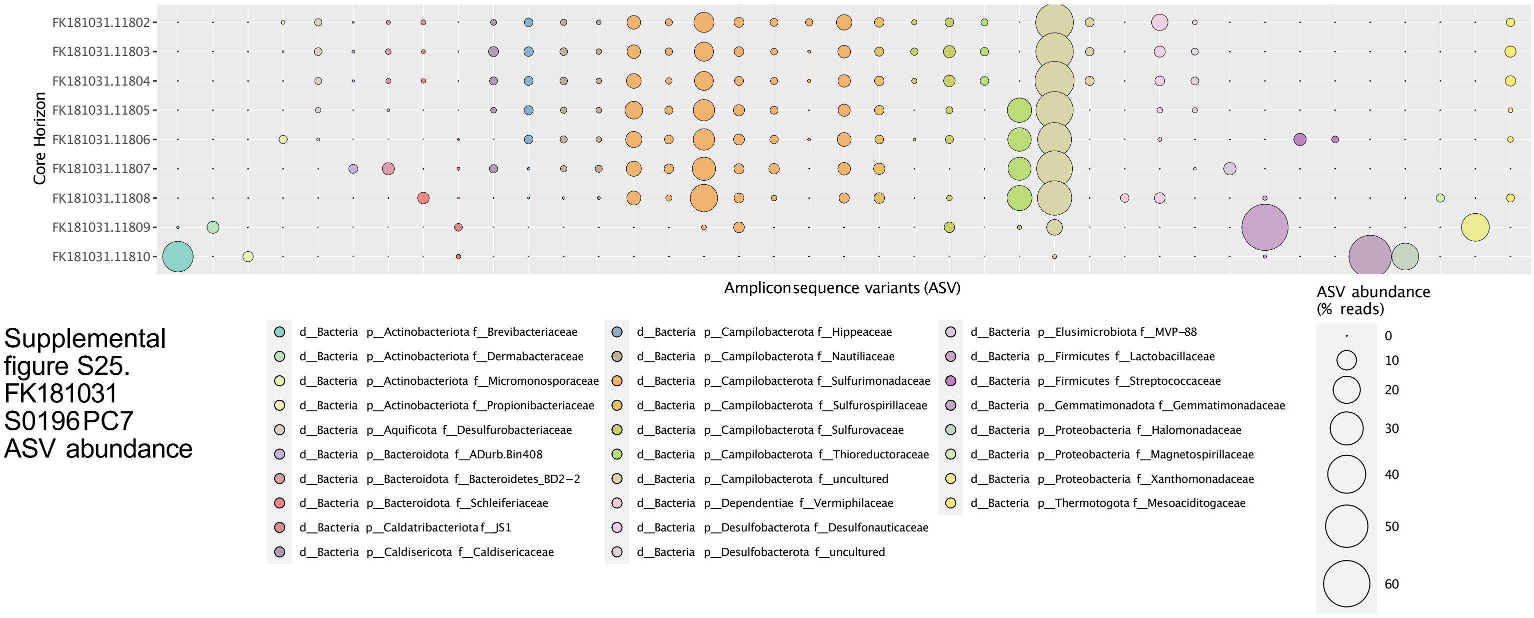

Supplemental figure S25. FK181031 S0196PC7 ASV abundance

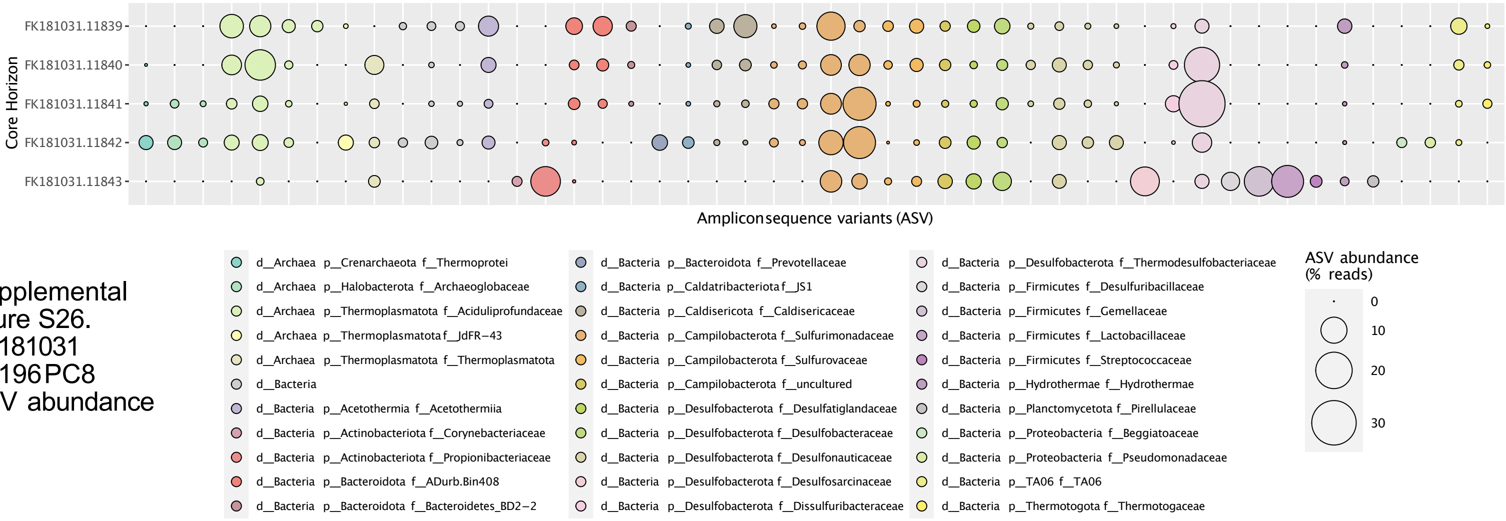

Supplemental figure S26. FK181031 S0196PC8 ASV abundance

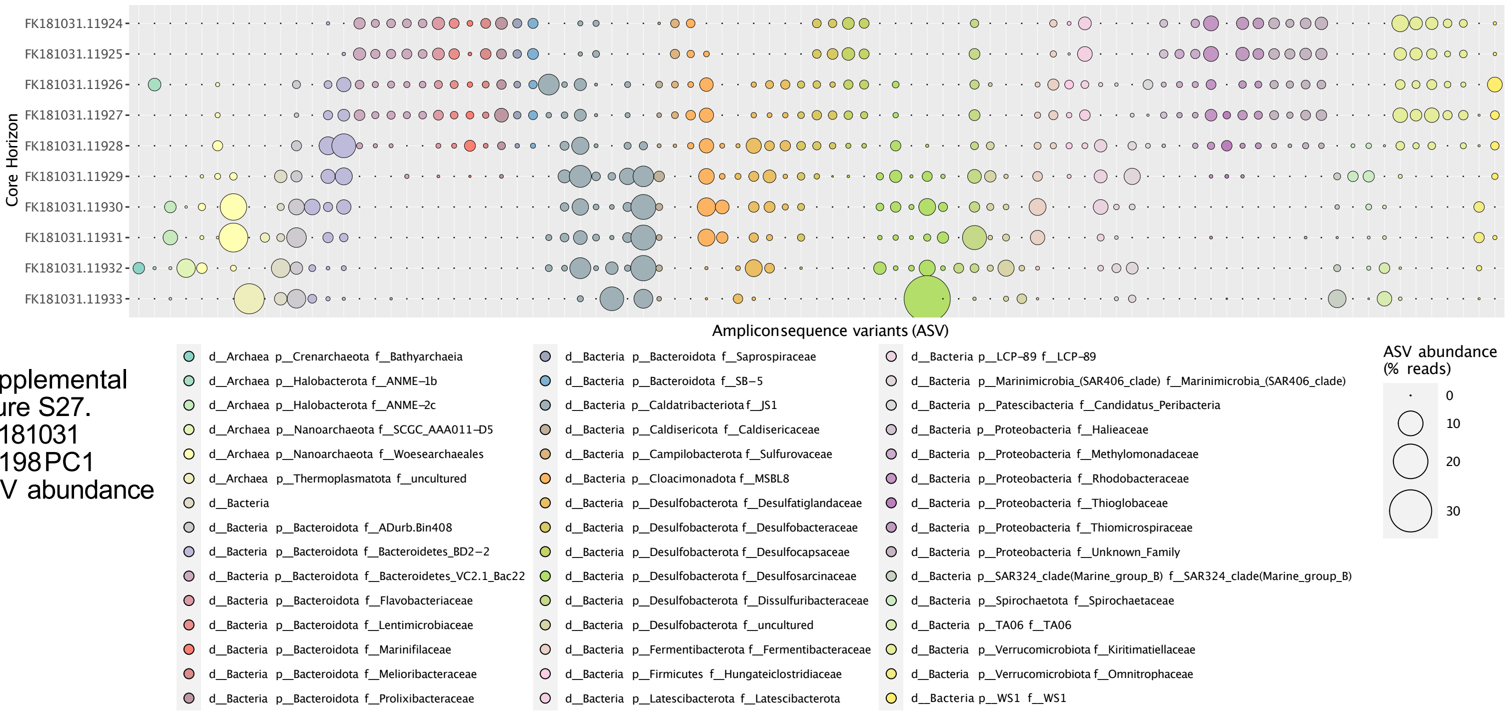

Supplemental figure S27. FK181031 S0198PC1 ASV abundance

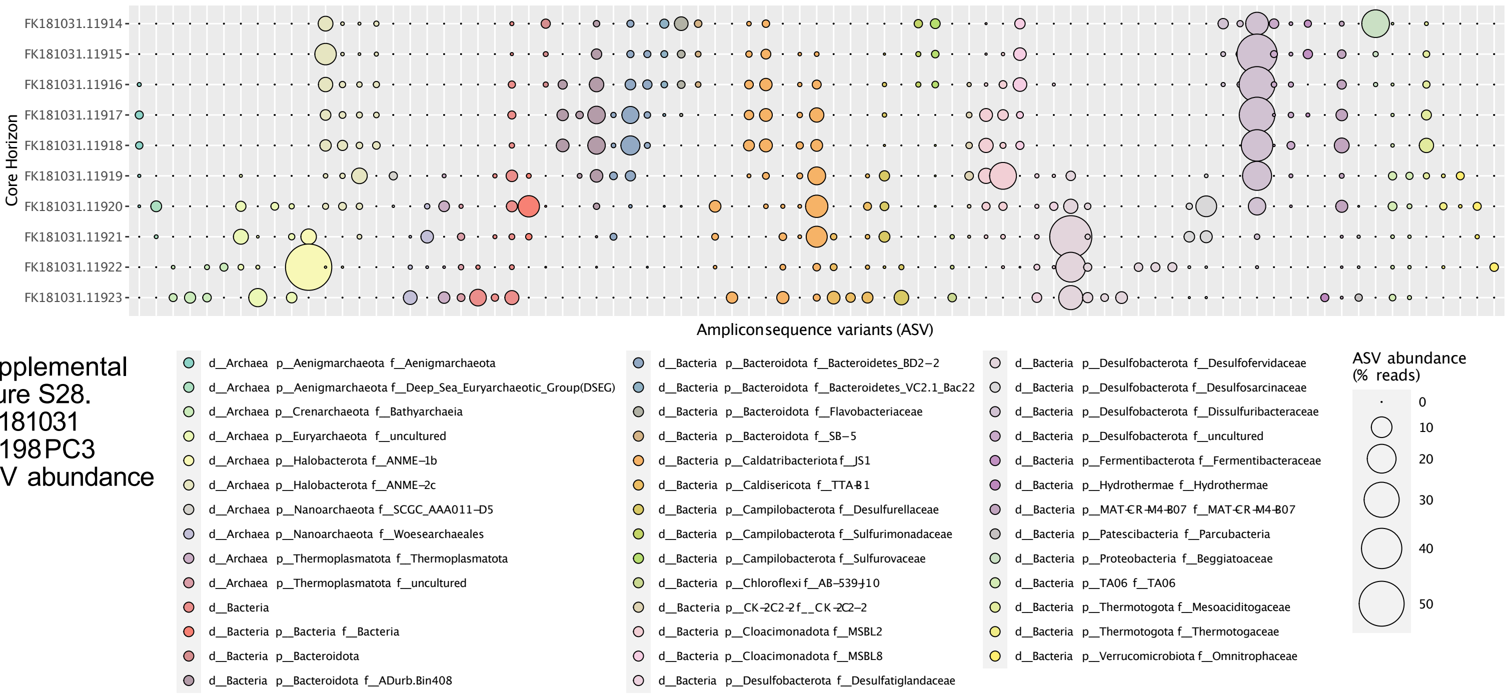

Supplemental figure S28. FK181031 S0198PC3 ASV abundance

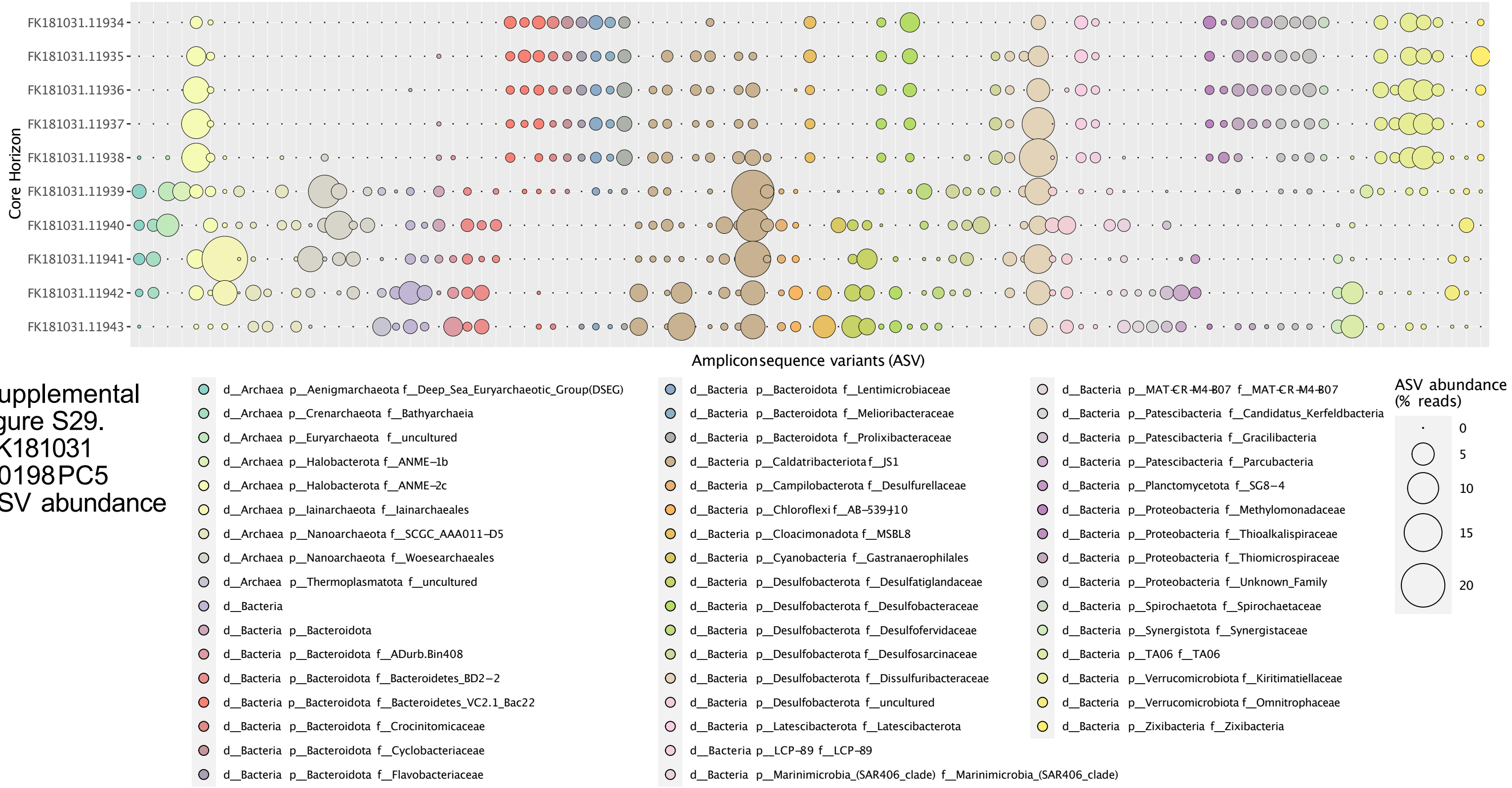

Supplemental figure S29. FK181031 S0198PC5 ASV abundance

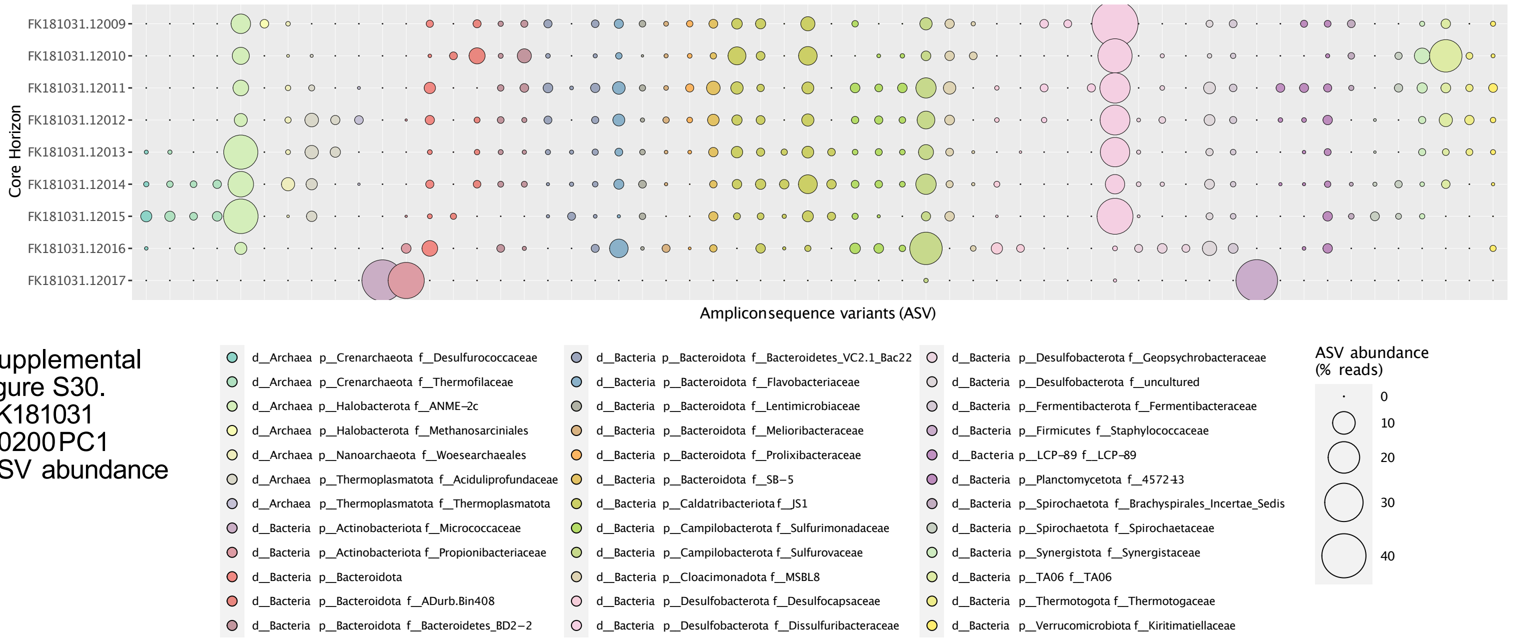

Supplemental figure S30. FK181031 S0200PC1 ASV abundance

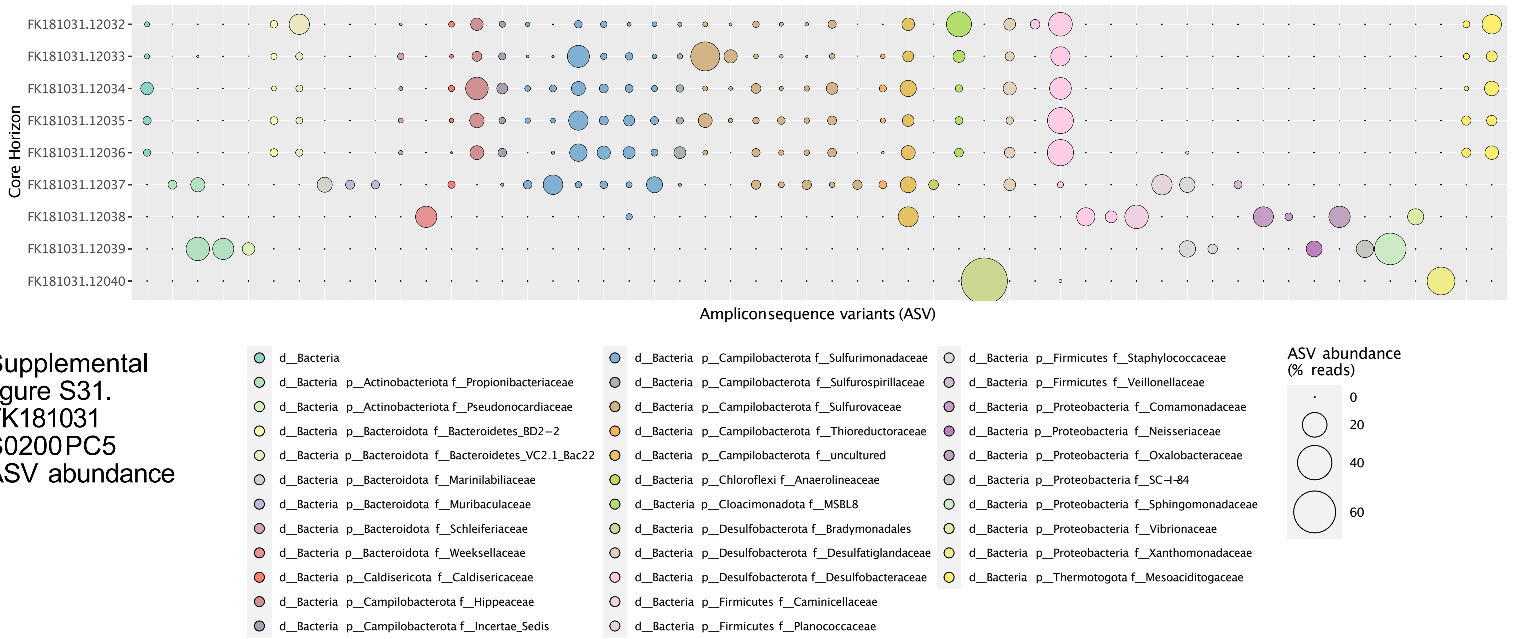

Supplemental figure S31. FK181031 S0200PC5 ASV abundance

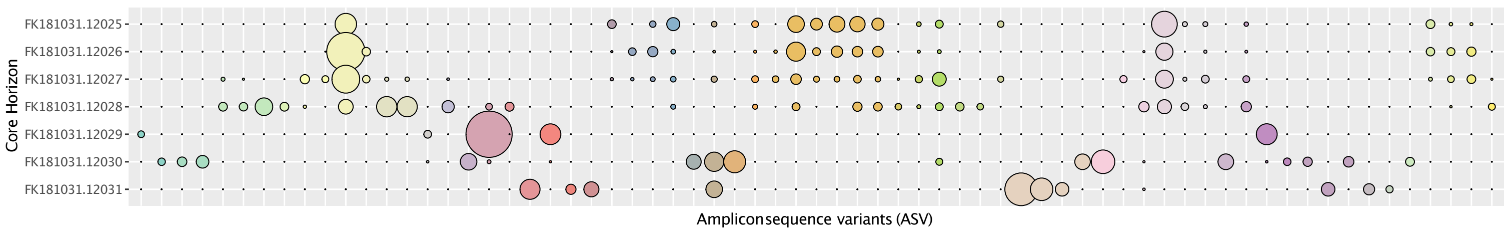

Supplemental figure S32. FK181031 S0200 PC7 ASV abundance

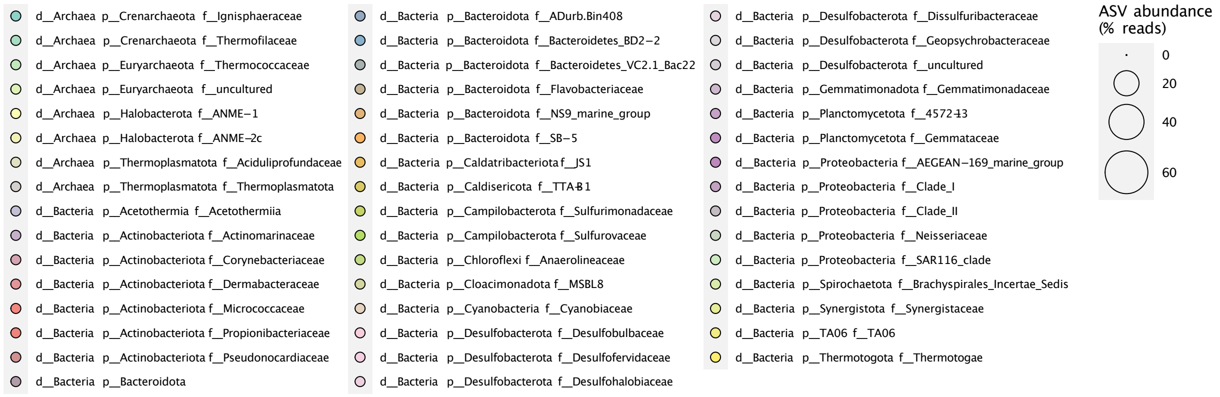

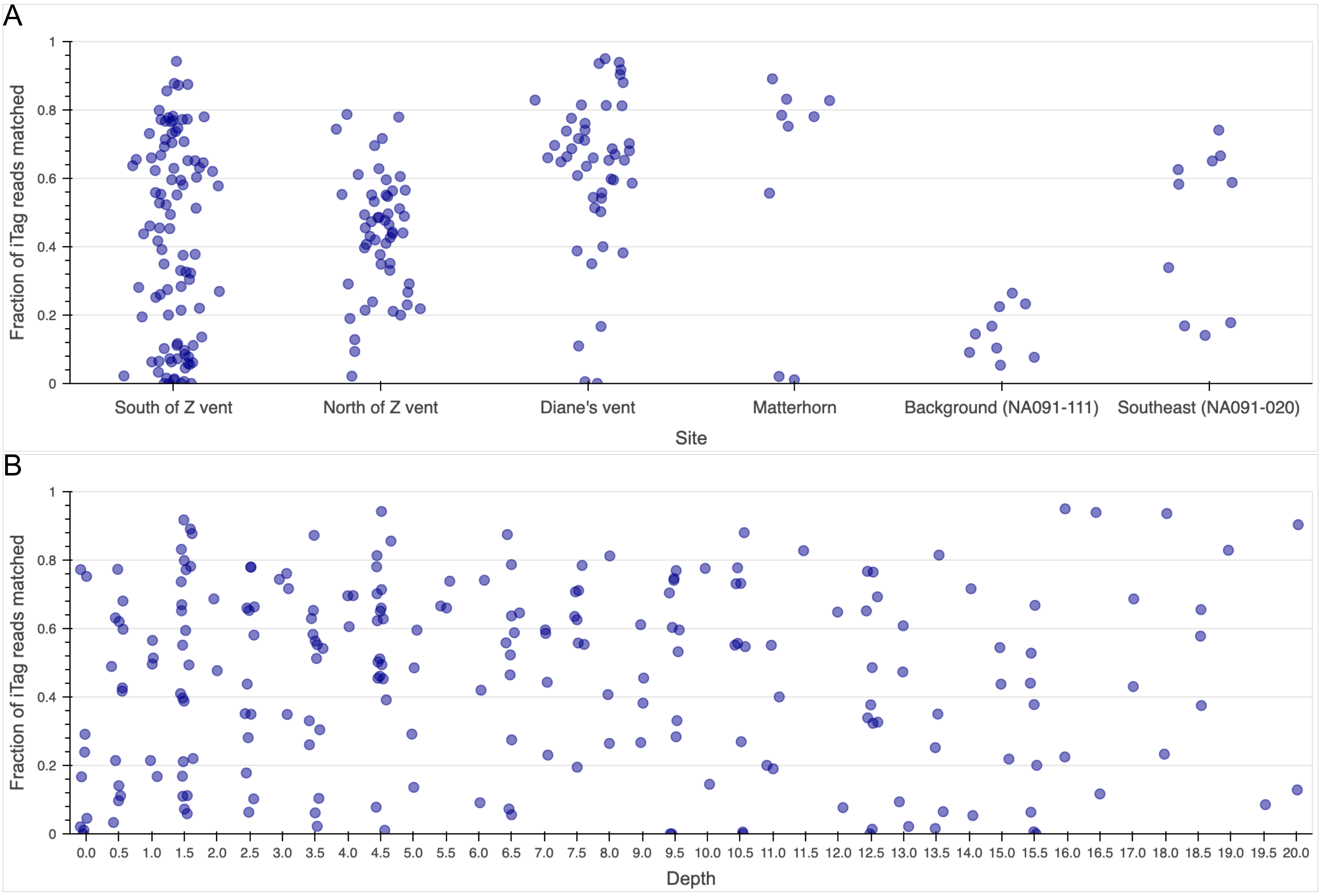

Supplemental figure S33. 16S rRNA gene amplicon reads matching the metagenome-derived 16S rRNA gene sequences.
Each dot in the strip plots represents a sediment horizon or mat sample, and indicates the fraction of amplicon reads matching the metagenome-derived 16S rRNA gene sequences with sequence identity >= 97%. Horizontal scatter was added for visual clarity, but has no meaning. Samples are shown by approximate location in the vent field (A) or by average depth of the sediment horizon (B).

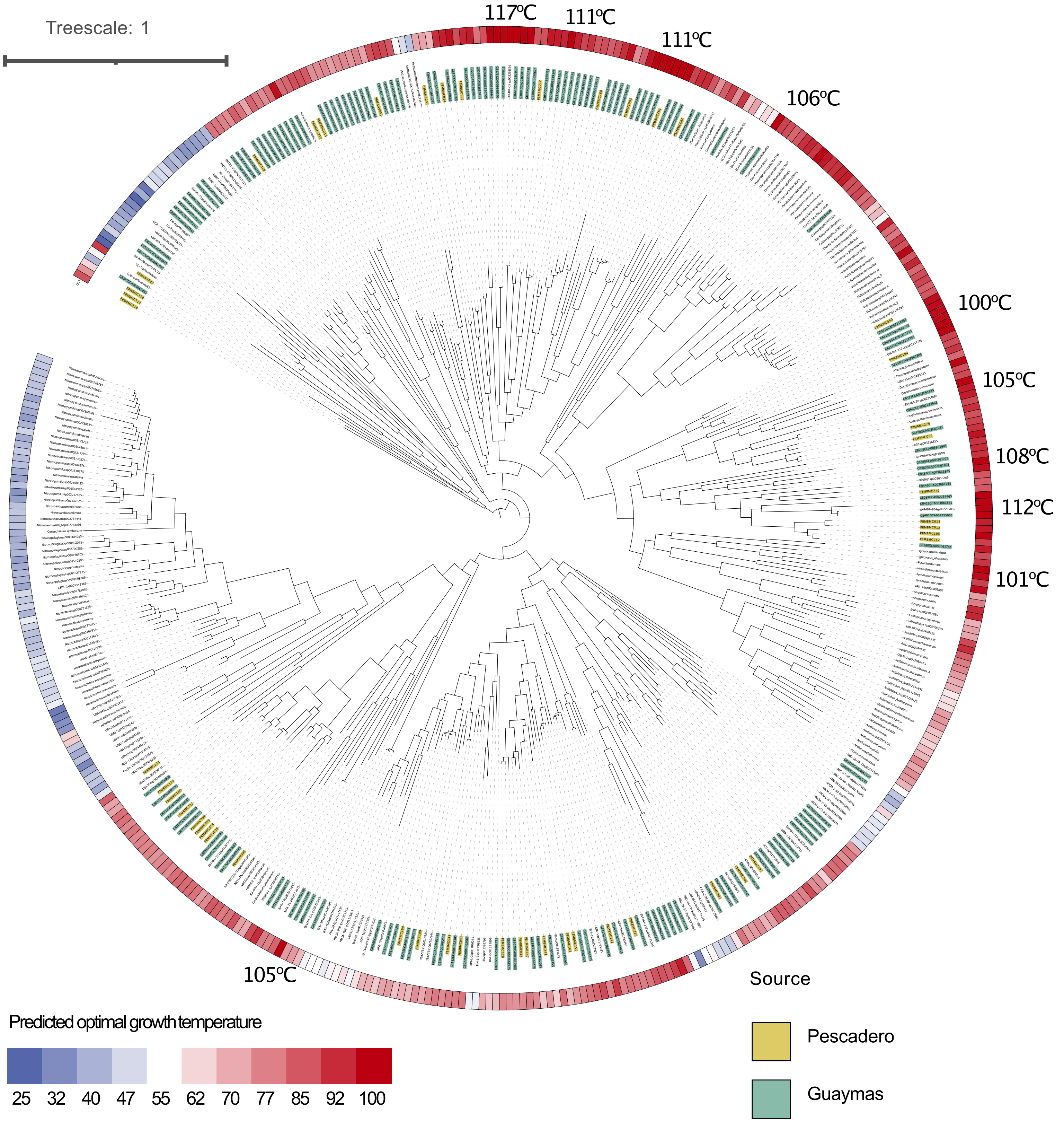

Supplemental Figure S34. TACK phylogeny and predicted optimal growth temperature

Concatenated marker gene phylogeny of all Asgardarchaeota and Crenarchaeota genomes from the genome taxonomydatabase (GTDB, v89), Guaymas basin (PRJNA362212) and Pescadero Basin (Auka, this study). The phylogeny was calculated using FastTree, on a concatenated alignment based on 76 Archaeal marker genes retrieved from the genomes using Anvi'o and aligned using Muscle. Optimal growth temperature (OGT) was predicted using the OGT prediction algorithm by Sauer and Wang (https://doi.org/10.1093/bioinformatics/btz059).PredictedOGTs above the scale maximumof 100°Careindicated.

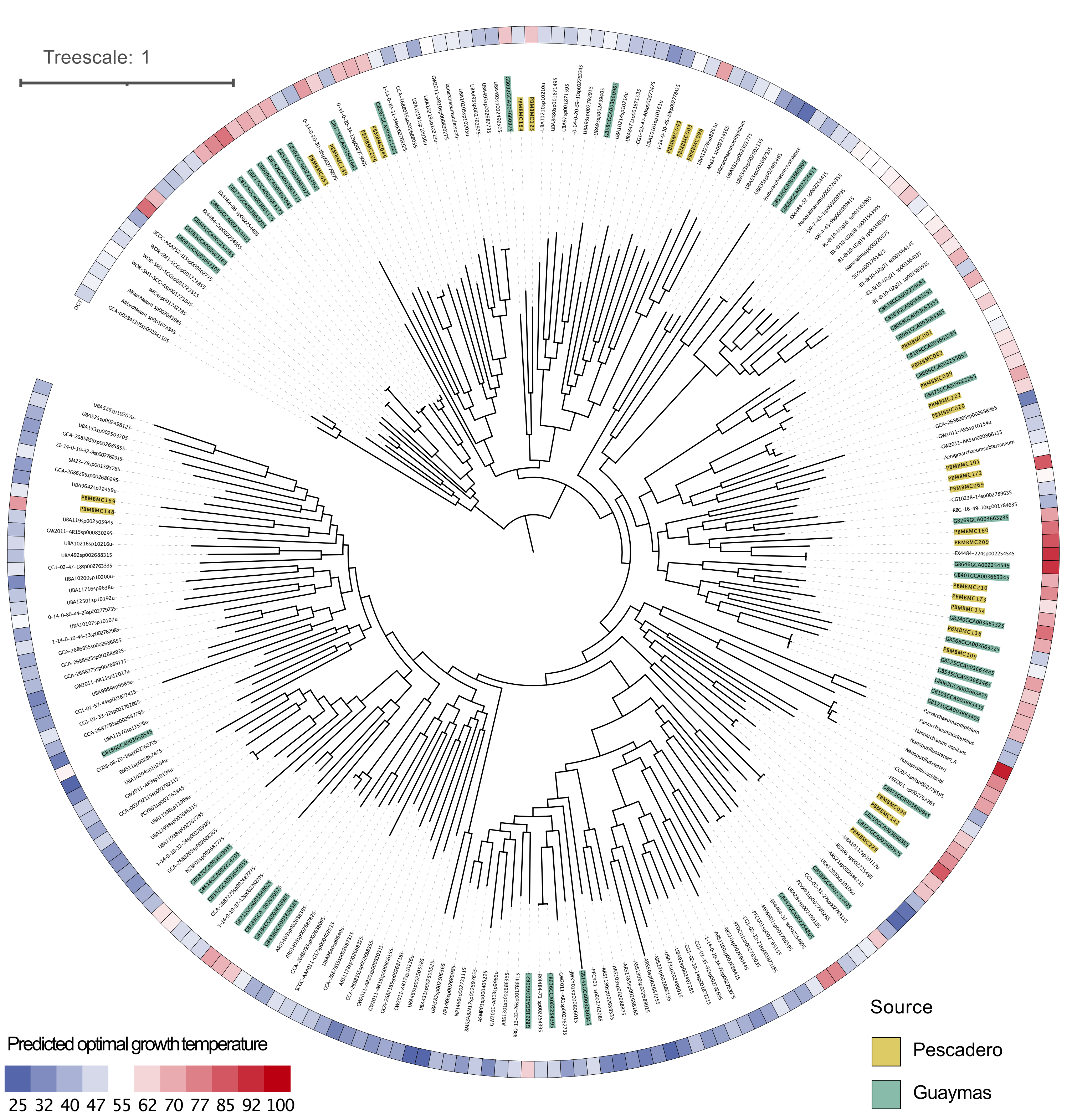

Supplemental Figure S35. DPANN phylogeny and predicted optimal growth temperature

Concatenated marker gene phylogeny of all Aenigmarchaeota, Altiarchaeota, EX4484_52, Huberarchaeaota, Iainarchaeota, Micrarchaeota, Nanoarchaeota, Nanohaloarchaeota, and UAP2 genomes from the genome taxonomy database (GTDB, v89), Guaymas basin (PRJNA362212) and Pescadero Basin (Auka, this study). The phylogeny was calculated using FastTree, on a concatenated alignment based on 76 Archaeal marker genes retrieved from the genomes using Anvi'o and aligned using Muscle. Optimal growth temperature was predicted using the OGT prediction by Sauer and Wang (https://doi.org/10.1093/bioinformatics/btz059).

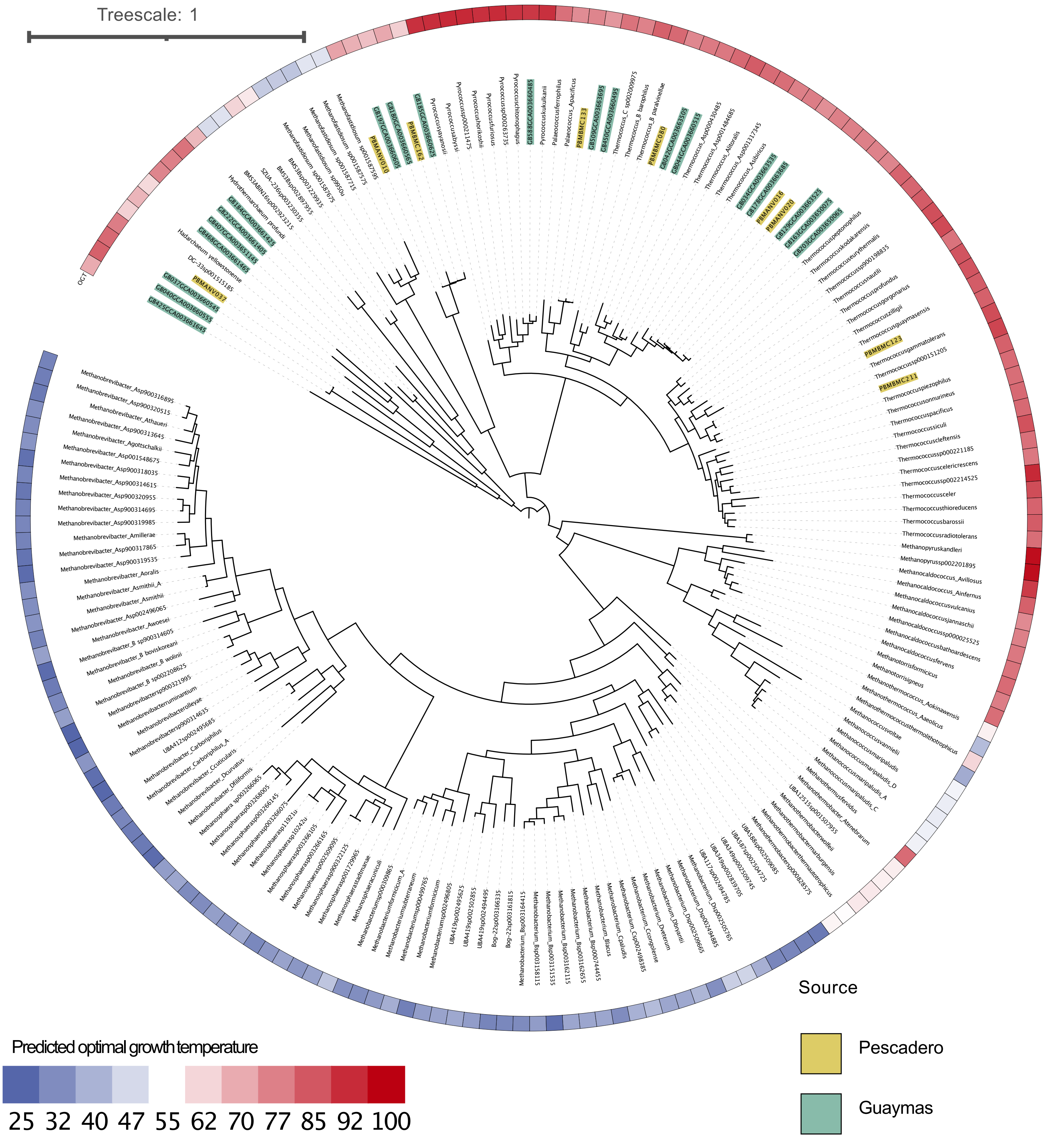

Supplemental Figure S36. Euryarchaeota phylogeny and predicted optimal growth temperature

Concatenated marker gene phylogeny of all Euryarchaeota, Hadarchaeota, and Hydrothermarchaeota genomes from the genome taxonomy database (GTDB, v89), Guaymas basin (PRJNA362212) and Pescadero Basin (Auka, this study). The phylogeny was calculated using FastTree, on a concatenated alignment based on 76 Archaeal marker genes retrieved from the genomes using Anvi'o and aligned using Muscle. Optimal growth temperature was predicted using the OGT prediction by Sauer and Wang (https://doi.org/10.1093/bioinformatics/btz059).

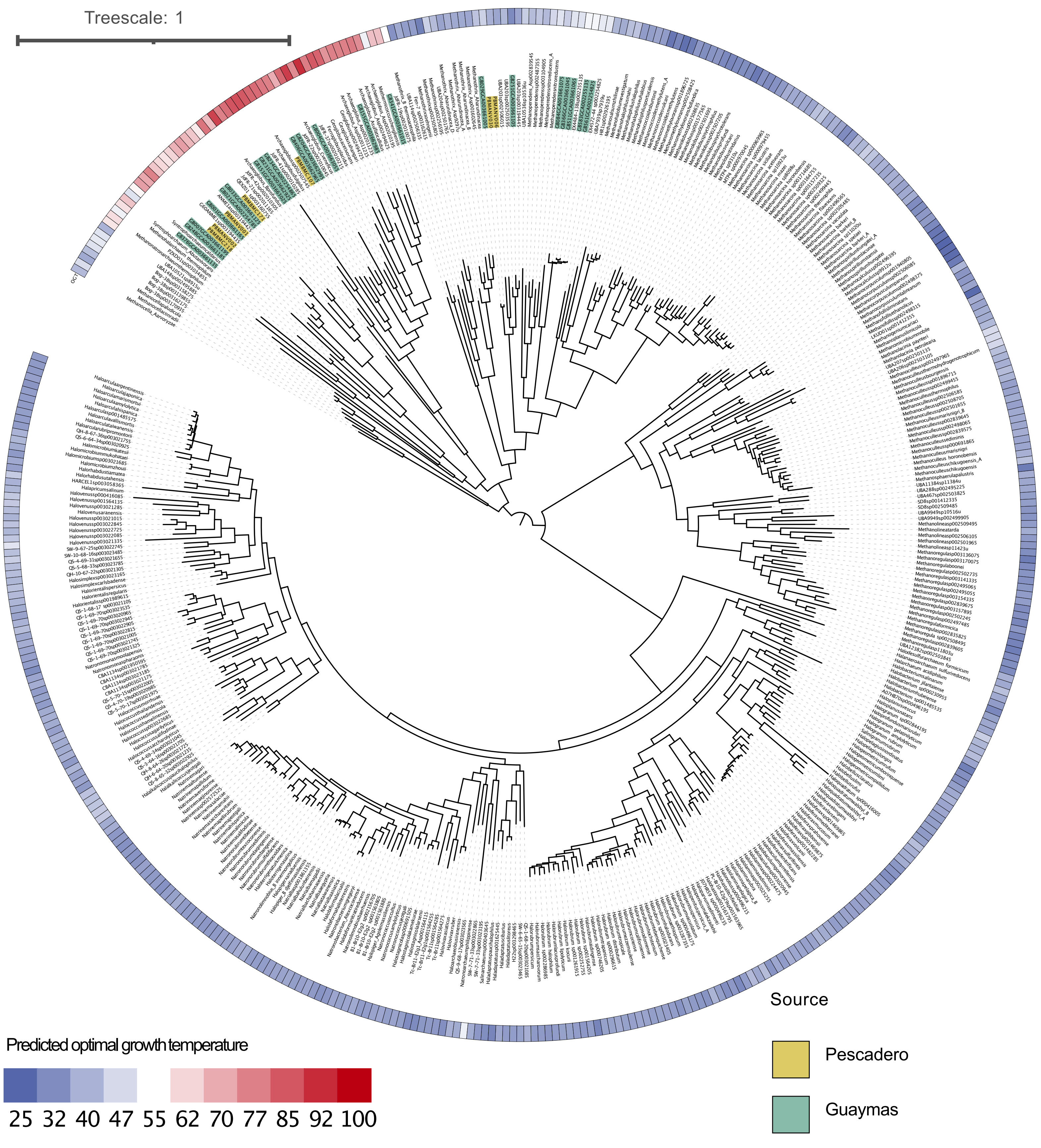

Supplemental Figure S37. Halobacterota phylogeny and predicted optimal growth temperature

Concatenated marker gene phylogeny of all Halobacterota genomes from the genome taxonomy database (GTDB, v89), Guaymas basin (PRJNA362212) and Pescadero Basin (Auka, this study). The phylogenywas calculated using FastTree, on a concatenated alignment based on 76 Archaeal marker genes retrieved from the genomes using Anvi'o and aligned using Muscle. Optimal growth temperature was predicted using the OGT prediction by Sauer and Wang (https://doi.org/10.1093/bioinformatics/btz059).

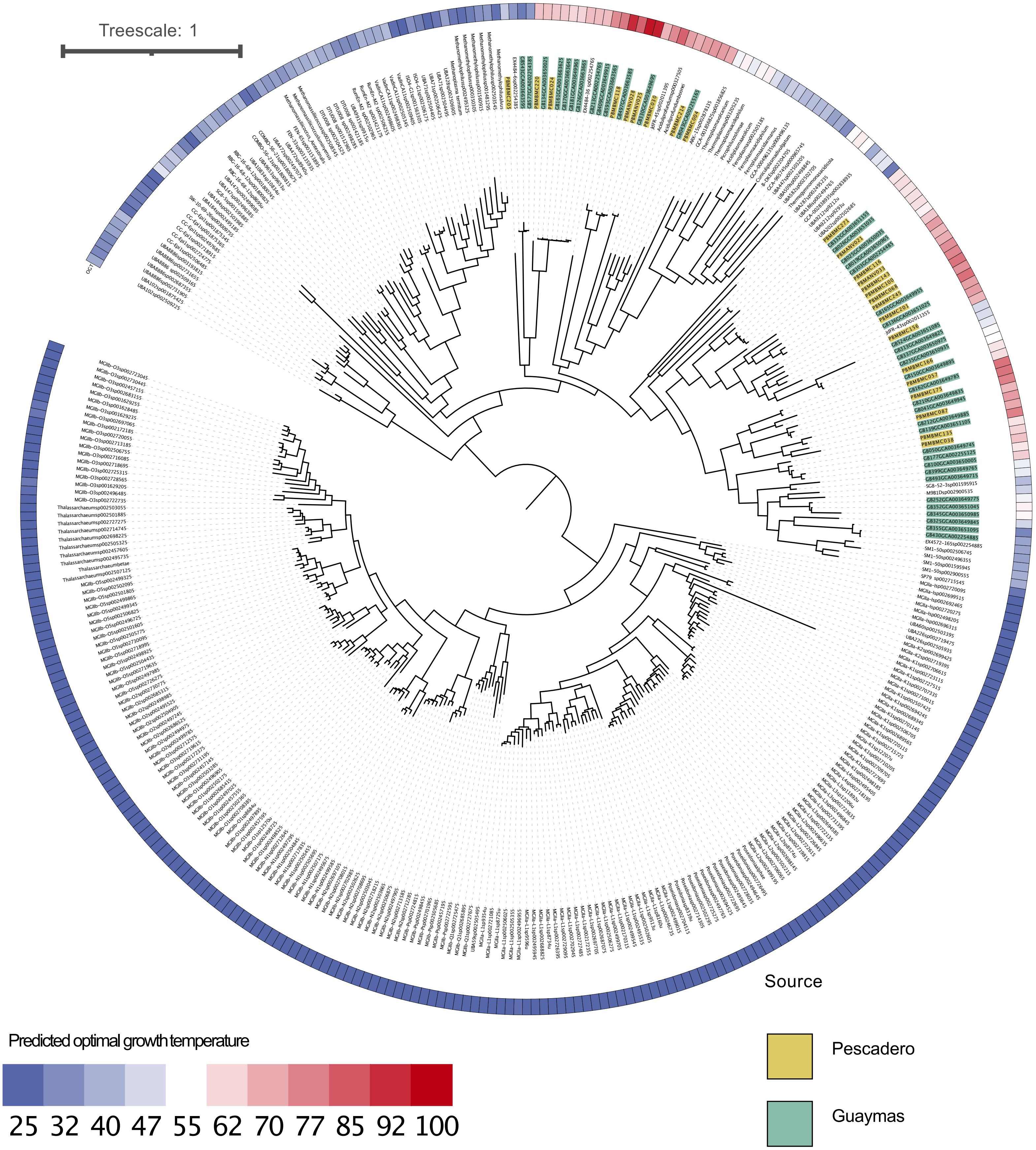

Supplemental Figure S38. Thermoplasmatota phylogeny and predicted optimal growth temperature

Concatenated marker gene phylogeny of all Thermoplasmatota genomes from the genome taxonomy database (GTDB, v89), Guaymas basin (PRJNA362212) and Pescadero Basin (Auka, this study). The phylogeny was calculated using FastTree, on a concatenated alignment based on 76 Archaeal marker genes retrieved from the genomes using Anvi'o and aligned using Muscle. Optimal growth temperature was predicted using the OGT prediction by Sauer and Wang (https://doi.org/10.1093/bioinformatics/btz059).

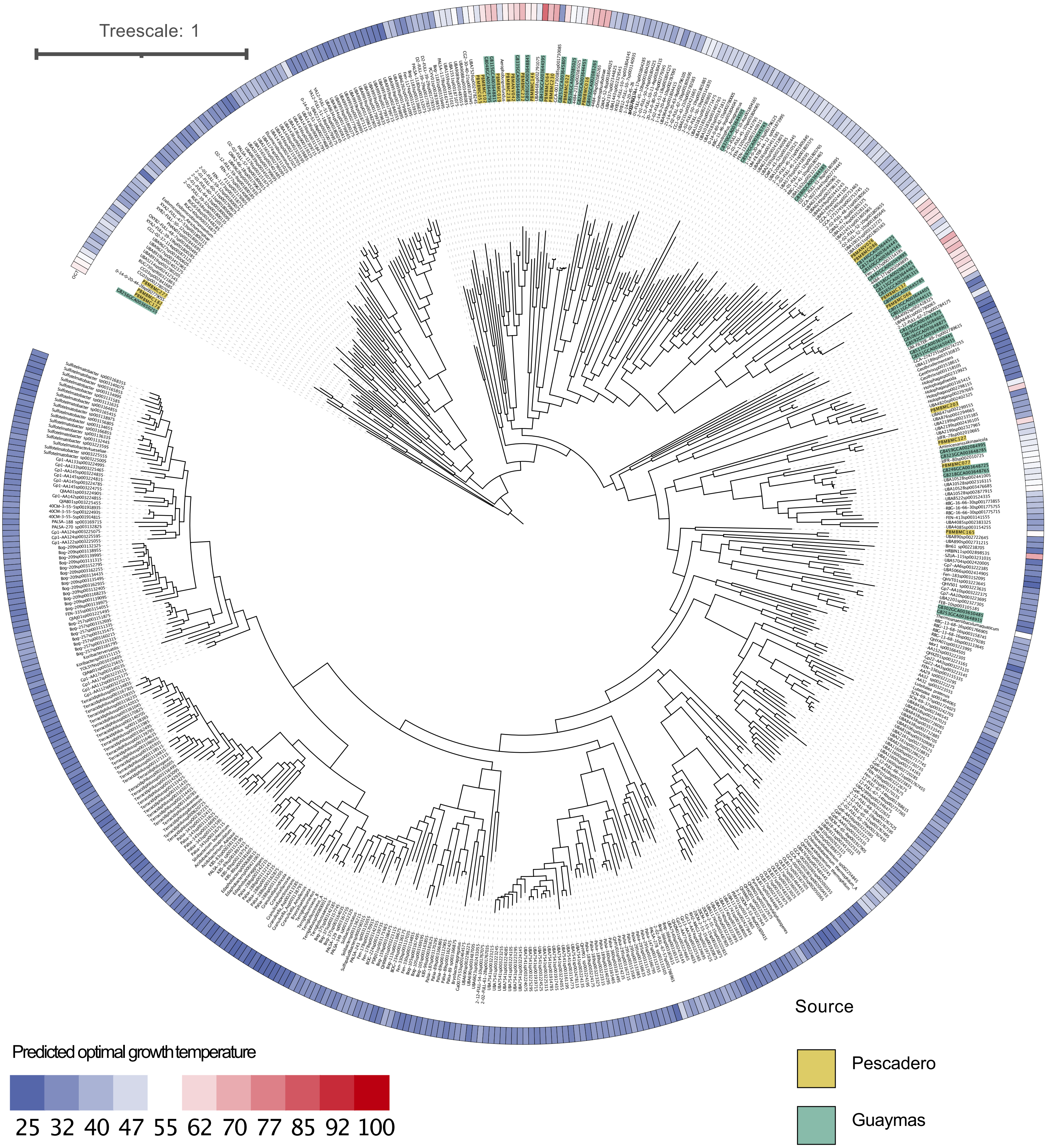

Supplemental Figure S39. Acidobacteriota phylogeny and predicted optimal growth temperature

Concatenated marker gene phylogeny of all Acidobacteriota, Aerophobota, CG03, Desantisbacteria, Elusimicrobiota, FCPU426, Firestonebacteria, GCA-001730085, Goldbacteria, Lindowbacteria, Omnitrophota, Ratteibacteria, RUG730, UBA6262, UBA8481, UBA9089, UBP18, and UBP4 genomes from the genome taxonomy database (GTDB, v89), Guaymas basin (PRJNA362212) and Pescadero Basin (Auka, this study). The phylogeny was calculated using FastTree, on a concatenated alignment based on 71 Bacterial marker genes retrieved from the genomes using Anvi'o and aligned using Muscle. Optimal growth temperature was predicted using the OGT prediction by Sauer and Wang (https://doi.org/10.1093/bioinformatics/btz059).

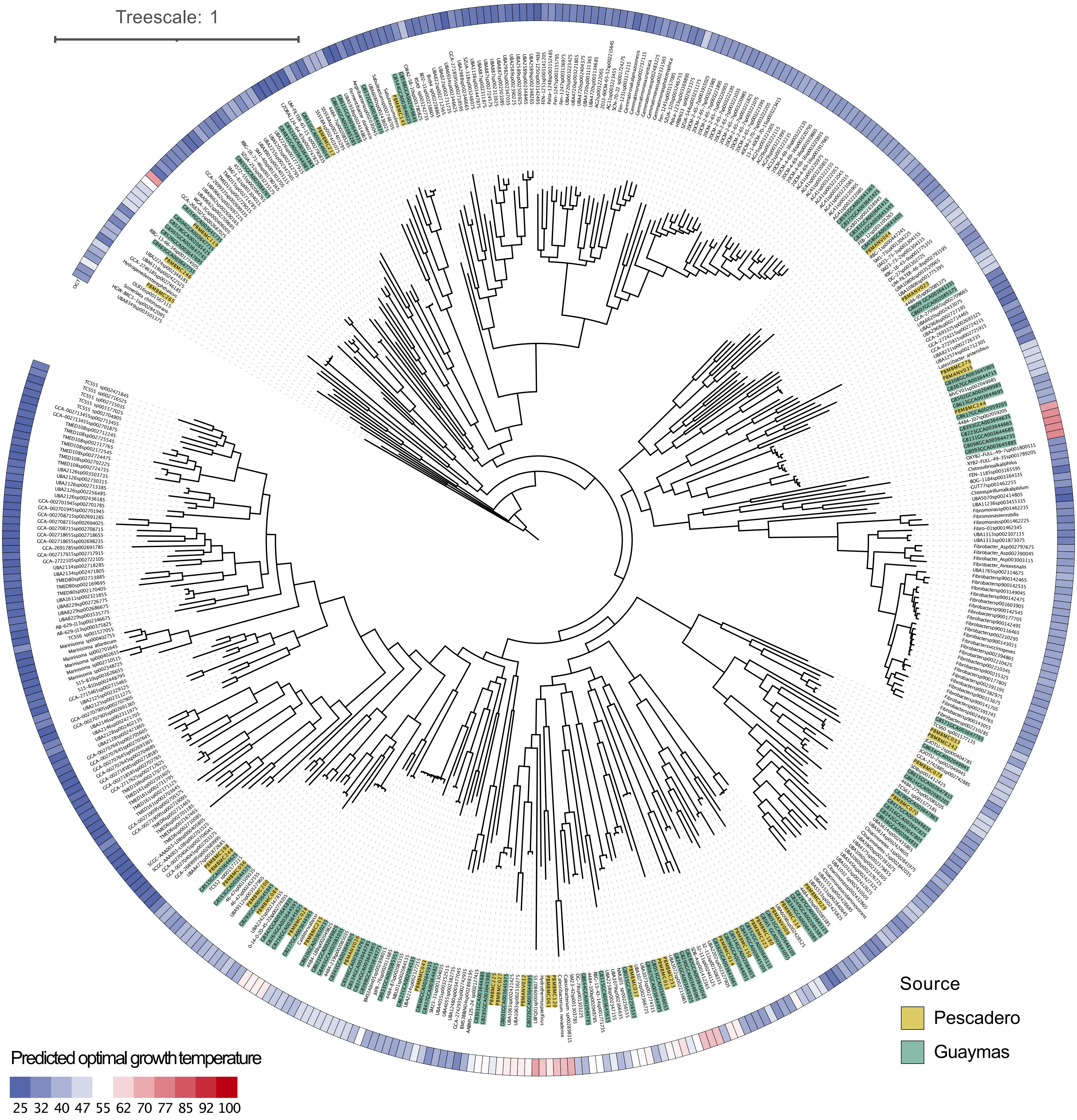

Supplemental Figure S40. Phylogeny and predicted optimal growth temperature of phyla basal to the Bacteroidota

Concatenated marker gene phylogeny of all 4572_22, AABM5-125-24, Calditrichota, Calescibacterota, Cloacimonadota, Delongbacteria, Edwardsbacteria, Eisenbacteria, Fermentibacterota, Fibrobacterota, Gemmatimonadota, Hydrogenedentota, Krumholzibacteriota, KSB1, Latescibacterota, Marinisomatota, OLB16, Poribacteria, RBG-13-66-14, SM23-31, Sumerlaeota, TA06_A, TA06, UBP14, WOR-3_A, WOR-3_B, WOR-3, and Zixibacteria genomes from the genome taxonomy database (GTDB, v89), Guaymas basin (PRJNA362212) and Pescadero Basin (Auka, this study). The phylogeny was calculated using FastTree, on a concatenated alignment based on 71 Bacterial marker genes retrieved from the genomes using Anvi'o and aligned using Muscle. Optimal growth temperature was predicted using the OGT prediction by Sauer and Wang (https://doi.org/10.1093/bioinformatics/btz059).

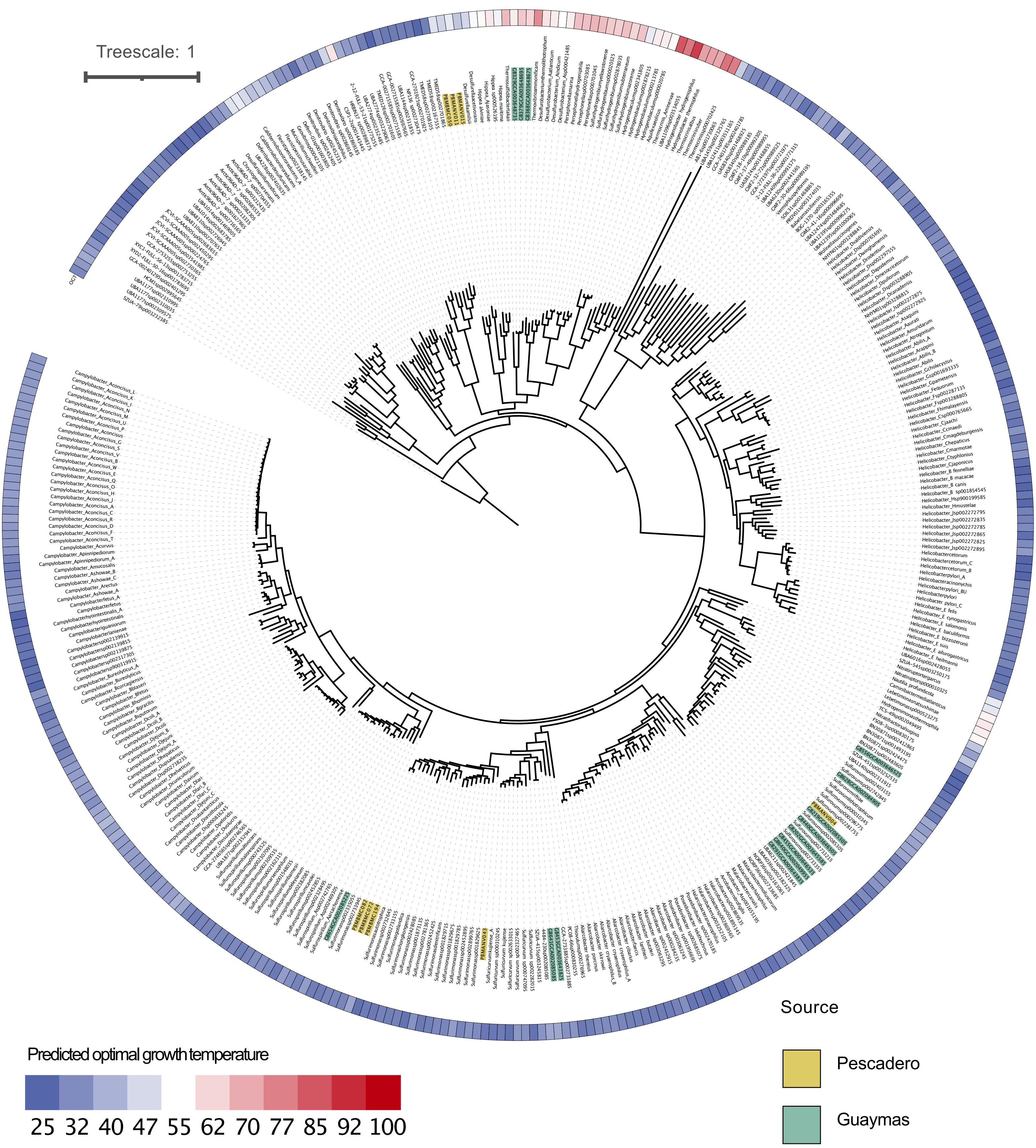

Supplemental Figure S41. Campylobacterota phylogeny and predicted optimal growth temperature

Concatenated marker gene phylogeny of all AB1-6, Aquificota, Campylobacterota, Chrysiogenetota, Dadabacteria, Deferribacterota, Dependentiae, SAR324, SZUA-79, Thermosulfidibacterota, and UBP6 genomes from the genome taxonomy database (GTDB, v89), Guaymas basin (PRJNA362212) and Pescadero Basin (Auka, this study). The phylogeny was calculated using FastTree, on a concatenated alignment based on 71 Bacterial marker genes retrieved from the genomes using Anvi'o and aligned using Muscle. Optimal growth temperature was predicted using the OGT prediction by Sauer and Wang (https://doi.org/10.1093/bioinformatics/btz059).

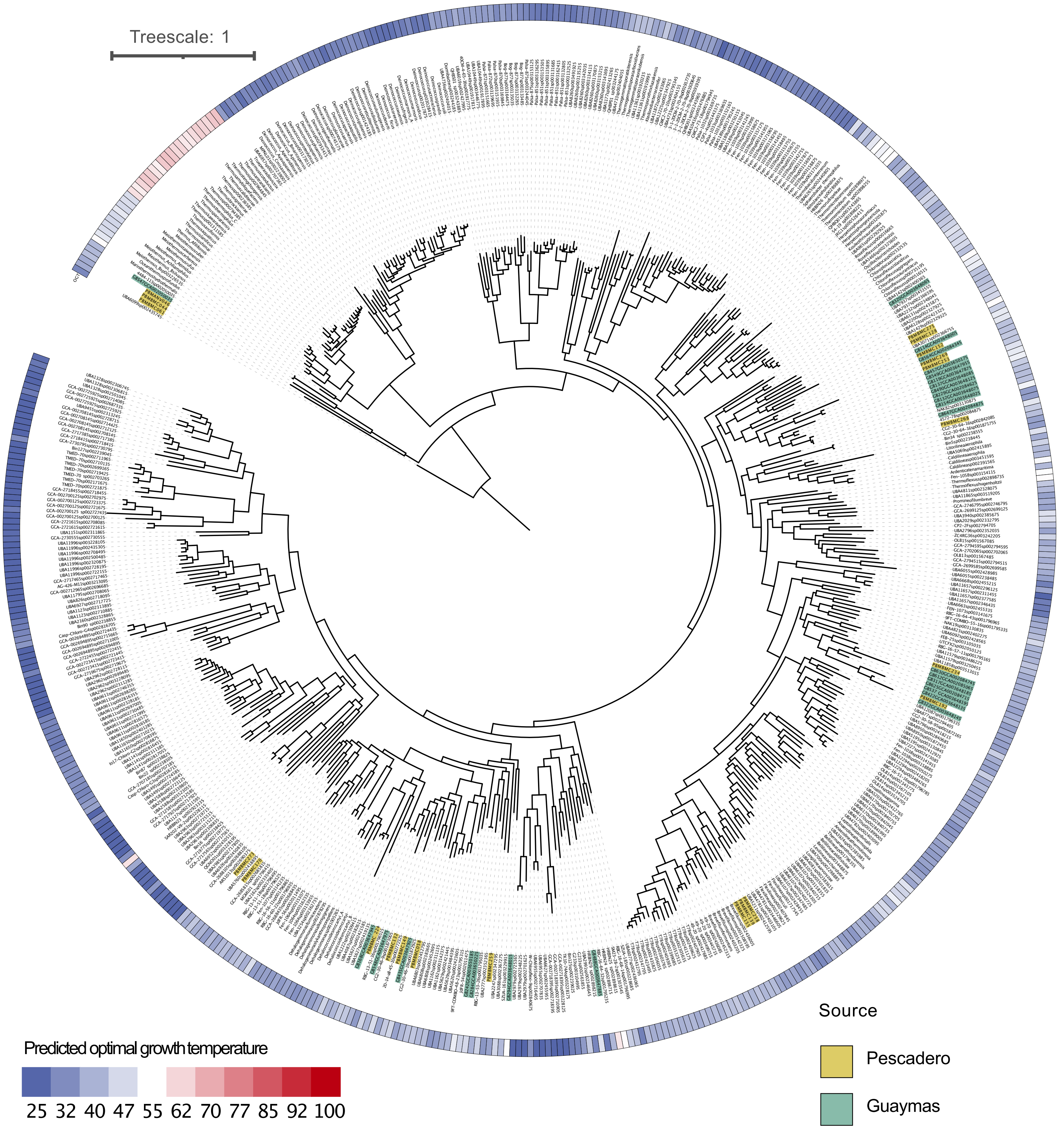

Supplemental Figure S42. Chloroflexota phylogeny and predicted optimal growth temperature

Concatenated marker gene phylogeny of all Chloroflexota_A, Chloroflexota_B, Chloroflexota, Deinococcota, Dormibacterota, UBP15, and UBP7_A genomes from the genome taxonomy database (GTDB, v89), Guaymas basin (PRJNA362212) and Pescadero Basin (Auka, this study). The phylogeny was calculated using FastTree, on a concatenated alignment based on 71 Bacterial marker genes retrieved from the genomes using Anvi'o and aligned using Muscle. Optimal growth temperature was predicted using the OGT prediction by Sauer and Wang (https://doi.org/10.1093/bioinformatics/btz059).

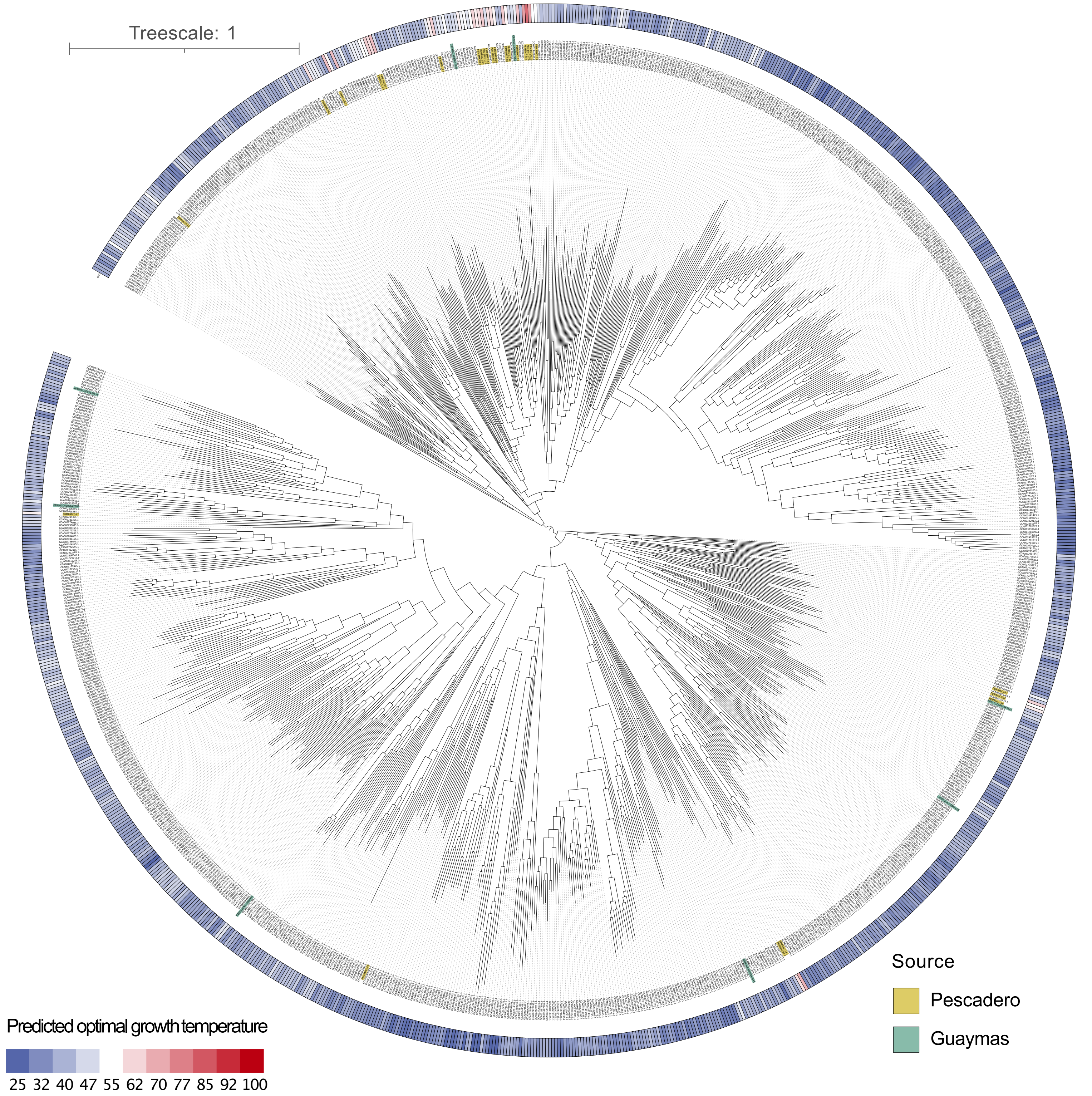

Supplemental Figure S43. Patescibacteria (CPR) phylogeny and predicted optimal growth temperature

Concatenated marker gene phylogeny of all Patescibacteria (CPR) genomes from the genome taxonomy database (GTDB, v89), Guaymas basin (PRJNA362212) and Pescadero Basin (Auka, this study). The phylogeny was calculated using FastTree, on a concatenated alignment based on 71 Bacterial marker genes retrieved from the genomes using Anvi'o and aligned using Muscle. Optimal growth temperature was predicted using the OGT prediction by Sauer and Wang (https://doi.org/10.1093/bioinformatics/btz059).

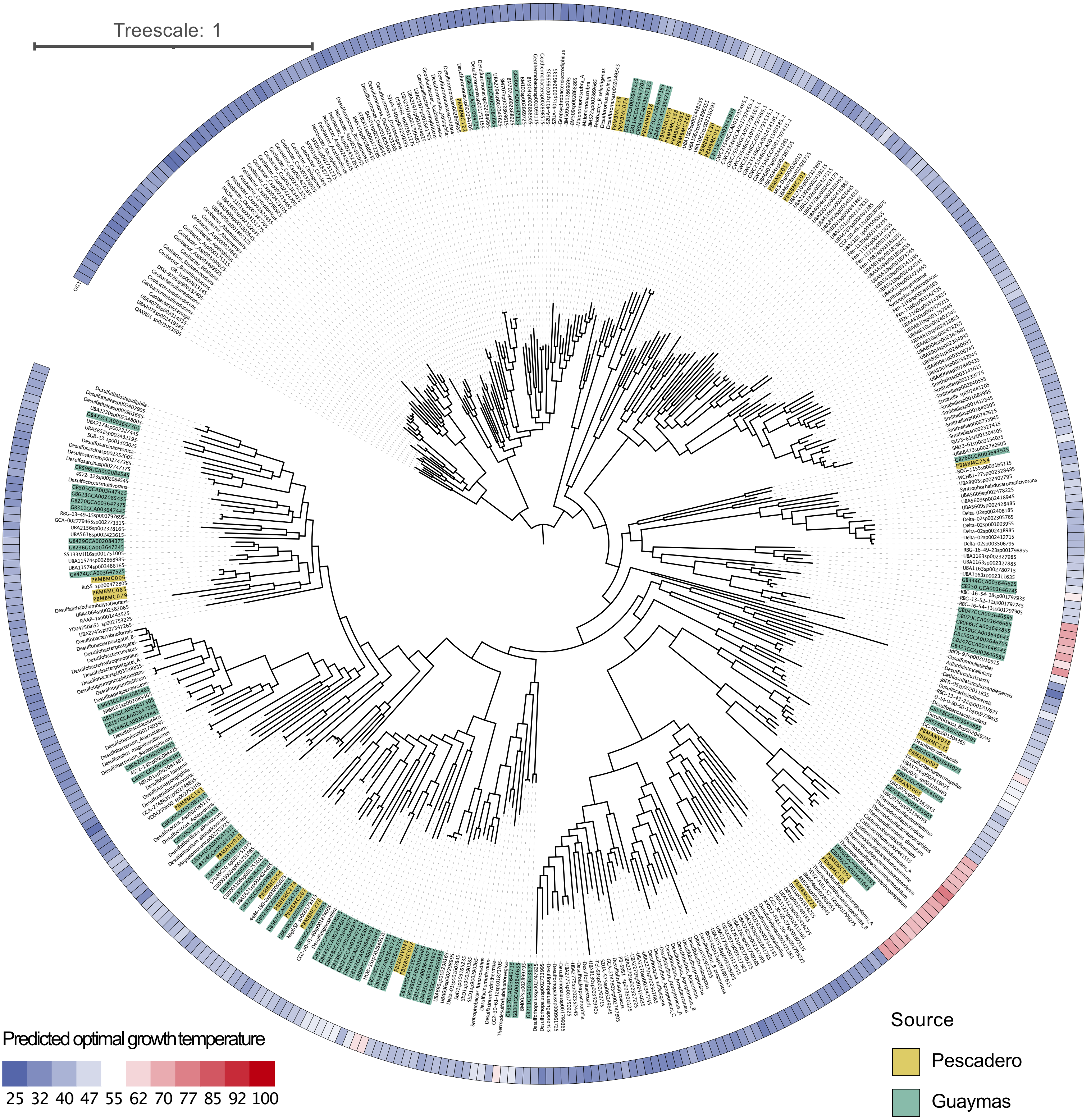

Supplemental Figure S44. Desulfobacterota phylogeny and predicted optimal growth temperature

Concatenated marker gene phylogeny of all Desulfobacterota_A, Desulfobacterota, Desulfuromonadota, and GWC2-55-46 genomes from the genome taxonomy database (GTDB, v89), Guaymas basin (PRJNA362212) and Pescadero Basin (Auka, this study). The phylogenywas calculated using FastTree, on aconcatenatedalignmentbasedon71Bacterialmarkergenesretrievedfromthegenomesusing Anvi'oand aligned using Muscle. Optimal growth temperature was predicted using the OGT prediction by Sauer and Wang (https://doi.org/10.1093/bioinformatics/btz059).

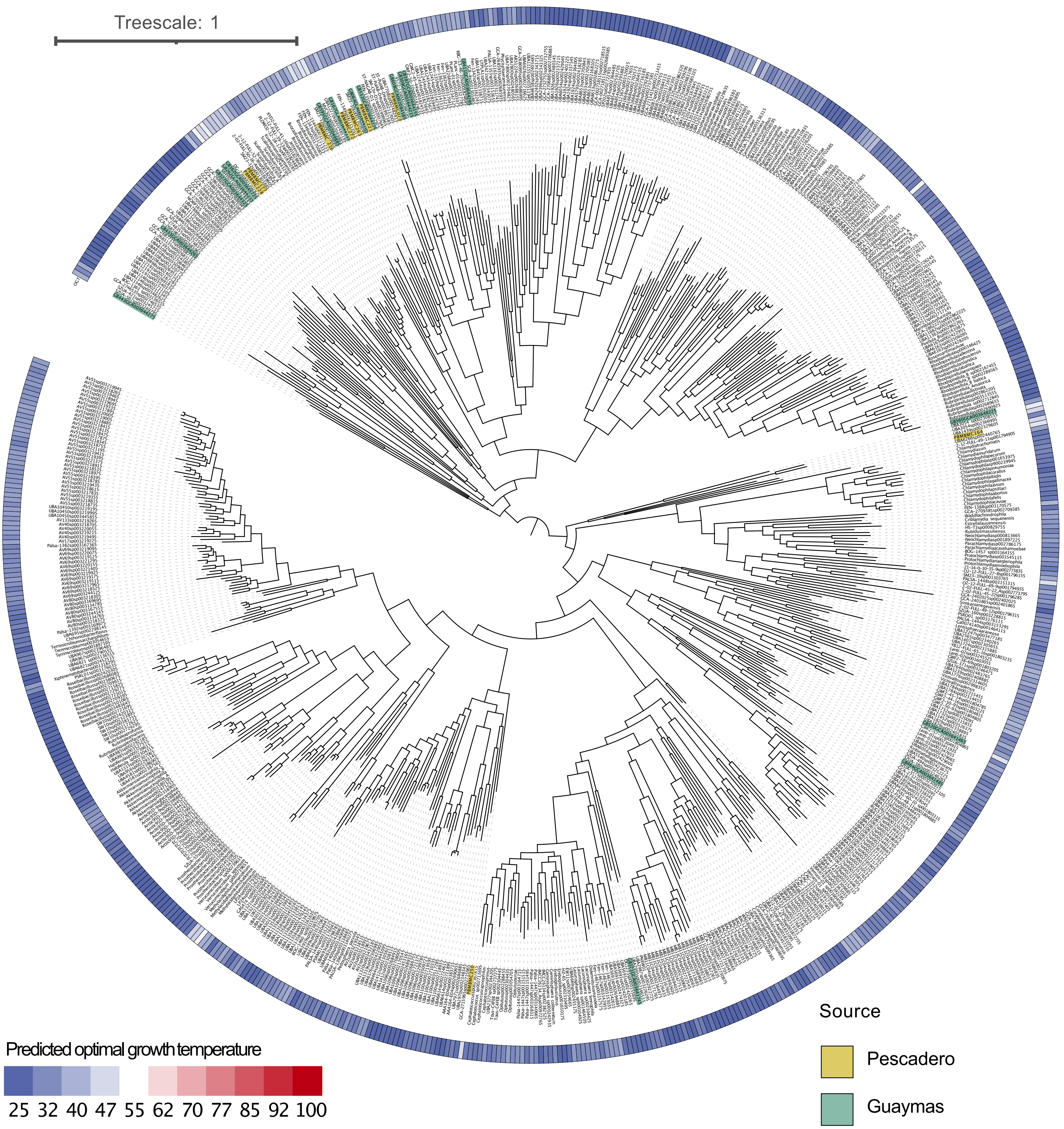

Supplemental Figure S45. PVC phylogeny and predicted optimal growth temperature

Concatenated marker gene phylogeny of all Planctomycetota, UBP3, Verrucomicrobiota_A, and Verrucomicrobiota genomes from the genome taxonomy database (GTDB, v89), Guaymas basin (PRJNA362212) and Pescadero Basin (Auka, this study). The phylogenywas calculated using FastTree, on aconcatenatedalignmentbasedon71Bacterialmarkergenesretrievedfromthegenomesusing Anvi'oand aligned using Muscle. Optimal growth temperature was predicted using the OGT prediction by Sauer and Wang (https://doi.org/10.1093/bioinformatics/btz059).

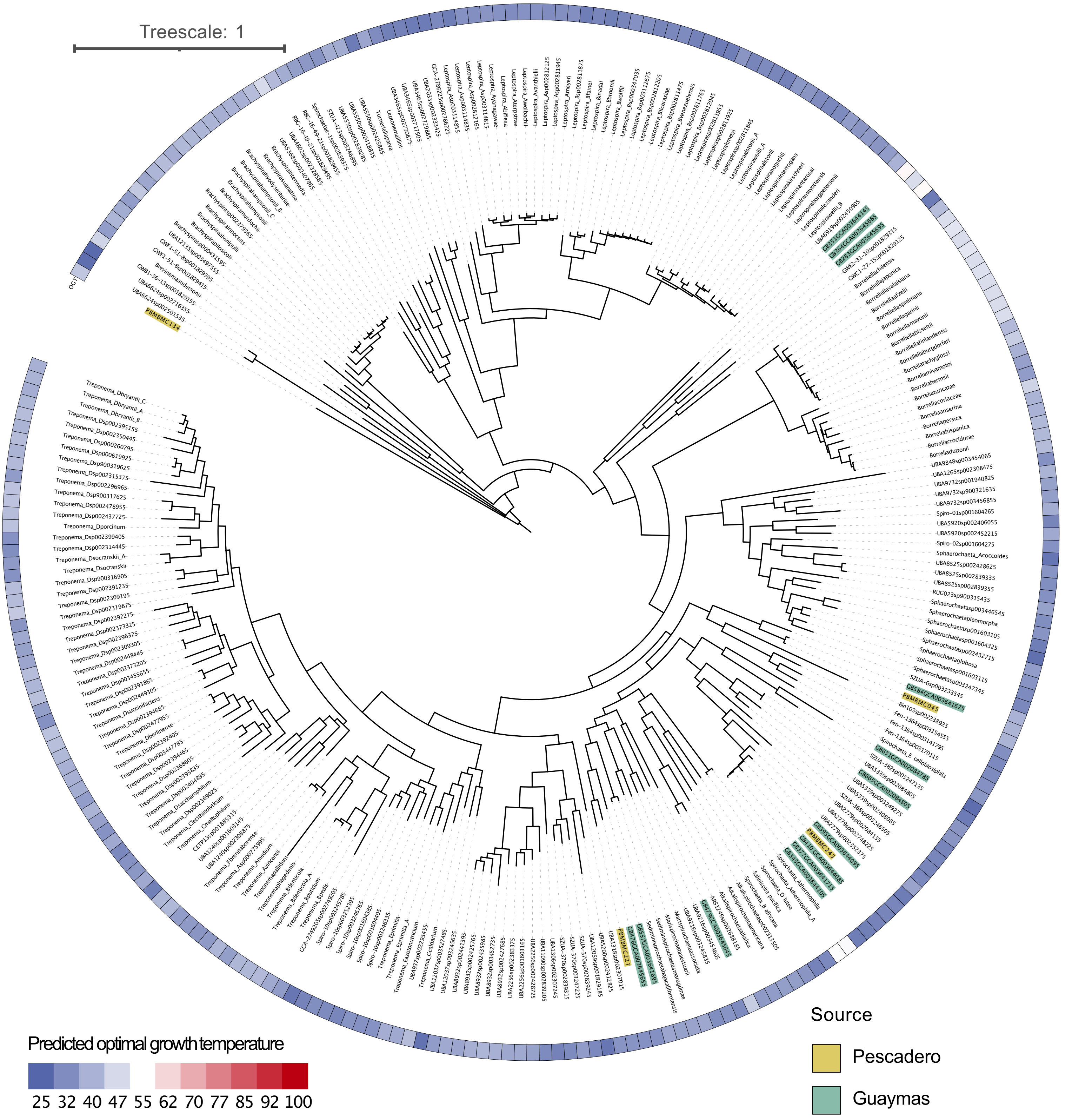

Supplemental Figure S46. Spirochaetota phylogeny and predicted optimal growth temperature

Concatenated marker gene phylogeny of all Spirochaetota and UBP7 genomes from the genome taxonomy database (GTDB, v89), Guaymas basin (PRJNA362212) and Pescadero Basin (Auka, this study). The phylogeny was calculated using FastTree, on a concatenated alignment based on 71 Bacterial marker genes retrieved from the genomes using Anvi'o and aligned using Muscle. Optimal growth temperature was predicted using the OGT prediction by Sauer and Wang (https://doi.org/10.1093/bioinformatics/btz059).

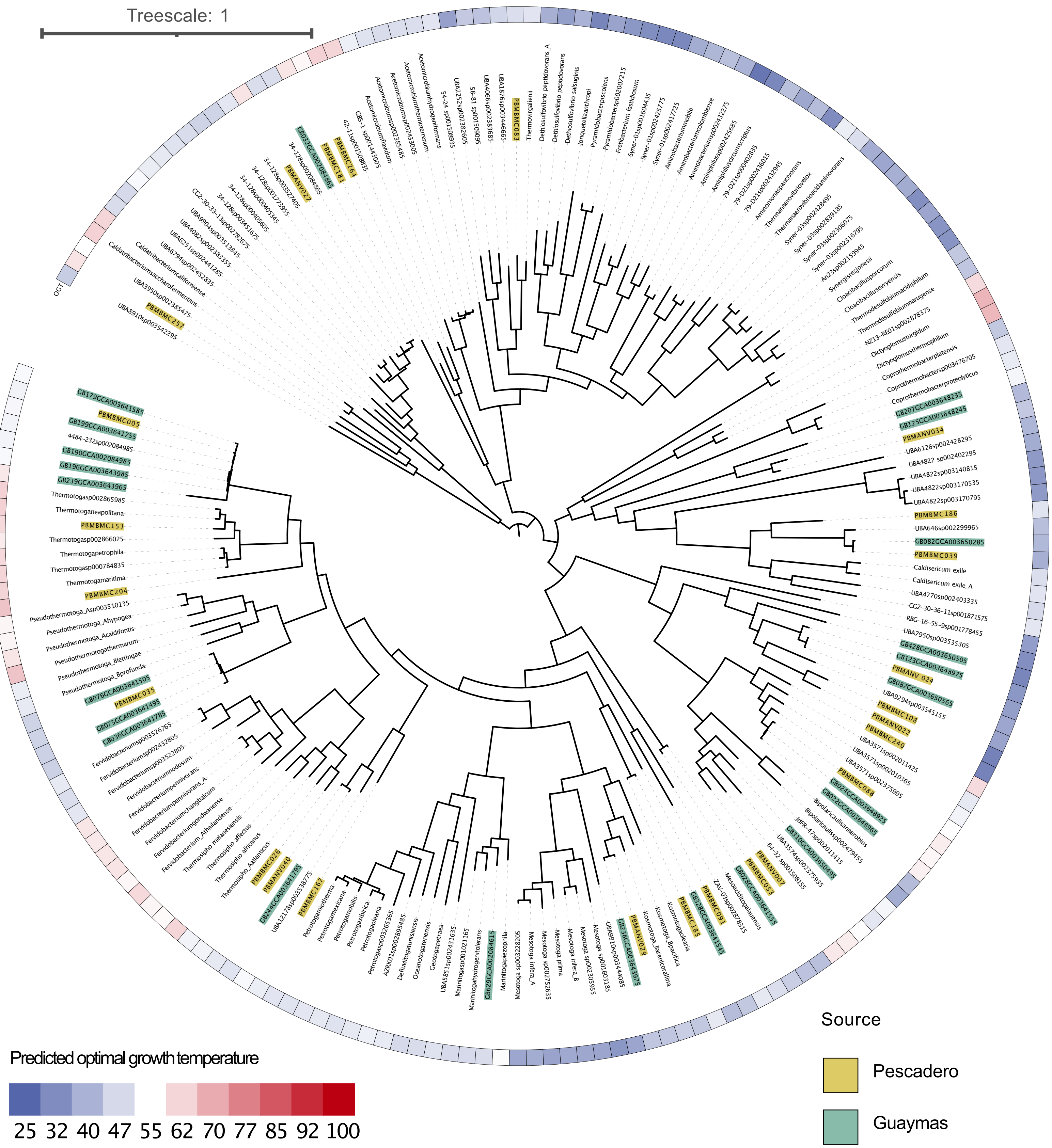

Supplemental Figure S47. Thermotogota phylogeny and predicted optimal growth temperature

Concatenated marker gene phylogeny of all Bipolaricaulota, Caldatribacteriota, Caldisericota, Coprothermobacterota, Dictyoglomota, Synergistota, Thermodesulfobiota, and Thermotogota genomes from the genome taxonomy database (GTDB, v89), Guaymas basin (PRJNA362212) and Pescadero Basin (Auka, this study). The phylogeny was calculated using FastTree, on a concatenated alignment based on 71 Bacterial marker genes retrieved from the genomes using Anvi'o and aligned using Muscle. Optimal growth temperature was predicted using the OGT prediction by Sauer and Wang (https://doi.org/10.1093/bioinformatics/btz059).

Supplemental text

Distinct sulfur oxidizing communities at Guaymas Basin and Auka

In stark contrast to the lineages shared between Auka and Guaymas Basin (see main text), a notable difference between the microbial communities of Auka vent field and Guaymas Basin are the sulfur oxidizing Bacteria (SOB) comprising the microbial mats on the sediment surface. These mats are dominated by *Beggiatoa* in Guaymas Basin (Gundersen et al. 1992; McKay et al. 2012) (~1700m depth) whereas the mat microbial community in the deeper Auka vent field (>3600m depth) is dominated by *Campylobacterota* (formerly *Epsilonproteobacteria*) related to *Sulfurimonas (Inagaki et al. 2003)* and *Sulfurovum (Inagaki et al. 2004)* (Supplemental figure S40). A similar depth trend was observed at the ultramafic hosted Mid-Cayman rise vents with *Gammaproteobacteria* (*Beggiatoa* and *Thiothrix*) dominating the shallower Von Damm community (2450m depth), and *Sulfurovum* dominant at the deeper Piccard vents (4950m depth) (Anderson et al. 2017). Yamamoto and Takai previously hypothesized *Campylobacterota* SOB have a wider ecophysiological range than *Gammaproteobacteria* SOB based on their ability to both oxidize and reduce sulfur cycle intermediates (Yamamoto and Takai 2011), suggesting differences in fluid chemistry could account for the observed depth trend. However, given the striking differences in hydrothermal fluid composition between sediment-hosted and ultramafic-hosted vents (McDermott et al. 2018), it is unclear whether fluid composition can explain the depth trend observed at both locations.

References to the Supplemental text

Anderson, Rika E., Julie Reveillaud, Emily Reddington, Tom O. Delmont, A. Murat Eren, Jill M. McDermott, Jeff S. Seewald, and Julie A. Huber. 2017. “Genomic Variation in Microbial Populations Inhabiting the Marine Subseafloor at Deep-Sea Hydrothermal Vents.” *Nature Communications* 8 (1): 1114.

Gundersen, Jens K., Bo Barker Jorgensen, Einer Larsen, and Holger W. Jannasch. 1992. “Mats of Giant Sulphur Bacteria on Deep-Sea Sediments due to Fluctuating Hydrothermal Flow.” *Nature* 360 (6403): 454–56.

Inagaki, Fumio, Ken Takai, Hideki Kobayashi, Kenneth H. Nealson, and Koki Horikoshi. 2003. “Sulfurimonas Autotrophica Gen. Nov., Sp. Nov., a Novel Sulfur-Oxidizing Epsilon-Proteobacterium Isolated from Hydrothermal Sediments in the Mid-Okinawa Trough.” *International Journal of Systematic and Evolutionary Microbiology* 53 (Pt 6): 1801–5.

Inagaki, Fumio, Ken Takai, Kenneth H. Nealson, and Koki Horikoshi. 2004. “Sulfurovum Lithotrophicum Gen. Nov., Sp. Nov., a Novel Sulfur-Oxidizing Chemolithoautotroph within the Epsilon-Proteobacteria Isolated from Okinawa Trough Hydrothermal Sediments.” *International Journal of Systematic and Evolutionary Microbiology* 54 (Pt 5): 1477–82.

McDermott, Jill M., Sean P. Sylva, Shuhei Ono, Christopher R. German, and Jeffrey S. Seewald. 2018. “Geochemistry of Fluids from Earth’s Deepest Ridge-Crest Hot-Springs: Piccard Hydrothermal Field, Mid-Cayman Rise.” *Geochimica et Cosmochimica Acta* 228 (May): 95–118.

McKay, Luke J., Barbara J. MacGregor, Jennifer F. Biddle, Daniel B. Albert, Howard P. Mendlovitz, Daniel R. Hoer, Julius S. Lipp, Karen G. Lloyd, and Andreas P. Teske. 2012.

“Spatial Heterogeneity and Underlying Geochemistry of Phylogenetically Diverse Orange and White Beggiatoa Mats in Guaymas Basin Hydrothermal Sediments.” *Deep Sea Research Part I: Oceanographic Research Papers* 67 (September): 21–31.

Yamamoto, Masahiro, and Ken Takai. 2011. “Sulfur Metabolisms in Epsilon- and Gamma-Proteobacteria in Deep-Sea Hydrothermal Fields.” *Frontiers in Microbiology* 2 (September): 192.
